# New Zampanolide Mimics: Design, Synthesis, and Antiproliferative Evaluation

**DOI:** 10.3390/molecules25020362

**Published:** 2020-01-15

**Authors:** Guanglin Chen, Ziran Jiang, Qiang Zhang, Guangdi Wang, Qiao-Hong Chen

**Affiliations:** 1 Department of Chemistry, California State University, Fresno, CA 93740, USA; chen.bmc@gmail.com (G.C.); ziranjiang@mail.fresnostate.edu (Z.J.); 2 Department of Chemistry and RCMI Cancer Research Center, Xavier University of Louisiana, New Orleans, LA 70125, USA; qzhang1@xula.edu (Q.Z.); gwang@xula.edu (G.W.)

**Keywords:** natural product, anticancer agent, synthesis, zampanolide

## Abstract

Zampanolide is a promising microtubule-stabilizing agent (MSA) with a unique chemical structure. It is superior to the current clinically used MSAs due to the covalent nature of its binding to *β*-tubulin and high cytotoxic potency toward multidrug-resistant cancer cells. However, its further development as a viable drug candidate is hindered by its limited availability. More importantly, conversion of its chemically fragile side chain into a stabilized bioisostere is envisioned to enable zampanolide to possess more drug-like properties. As part of our ongoing project aiming to develop its mimics with a stable side chain using straightforward synthetic approaches, 2-fluorobenzyl alcohol was designed as a bioisosteric surrogate for the side chain based on its binding conformation as confirmed by the X-ray structure of tubulin complexed with zampanolide. Two new zampanolide mimics with the newly designed side chain have been successfully synthesized through a 25-step chemical transformation for each. Yamaguchi esterification and intramolecular Horner–Wadsworth–Emmons condensation were used as key reactions to construct the lactone core. The chiral centers at C17 and C18 were introduced by the Sharpless asymmetric dihydroxylation. Our WST-1 cell proliferation assay data in both docetaxel-resistant and docetaxel-naive prostate cancer cell lines revealed that compound **6** is the optimal mimic and the newly designed side chain can serve as a bioisostere for the chemically fragile *N*-acetyl hemiaminal side chain in zampanolide.

## 1. Introduction

Prostate cancer is still one of the big health concerns, as evidenced by its high incidence and mortality rate in American men. In 2019, 174,650 new prostate cancer cases that account for nearly 20% of all cancer cases and 31,620 prostate cancer deaths are projected to occur in the United States [1]. Prostate cancer is driven by the androgen receptor (AR)-regulated gene expression that is initiated by the binding of androgen to the AR [2]. Androgen deprivation therapy (ADT) has thus been the main treatment for prostate cancer over 70 years. However, the median duration of ADT response is merely 18–24 months due to inevitable progression to castration-resistant prostate cancer (CRPC) [3]. Consequently, approximately 30,000 prostate cancer deaths in the United State each year are caused by CRPC as well as resistance to the current treatments. The critical driving force for the continued progression of lethal CRPC is the reactivation of AR transcriptional activity [4,5]. The current first-line and second-line chemotherapeutics for CRPC are two microtubule-stabilizing cytotoxic agents (MSA), docetaxel and cabazitaxel [6,7,8]. Promotion of mitotic arrest is the initially well-established mechanism underlying the action of MSAs as anticancer agents [9]. The MSAs were recently revealed to impair AR transcriptional activity by suppressing nuclear importation of AR as downstream of microtubule stabilization [10]. This alternative mechanism is independent of MSA-induced mitotic arrest and can explain in part the efficacy of MSA in AR-driven CRPC.

**Figure 1:**
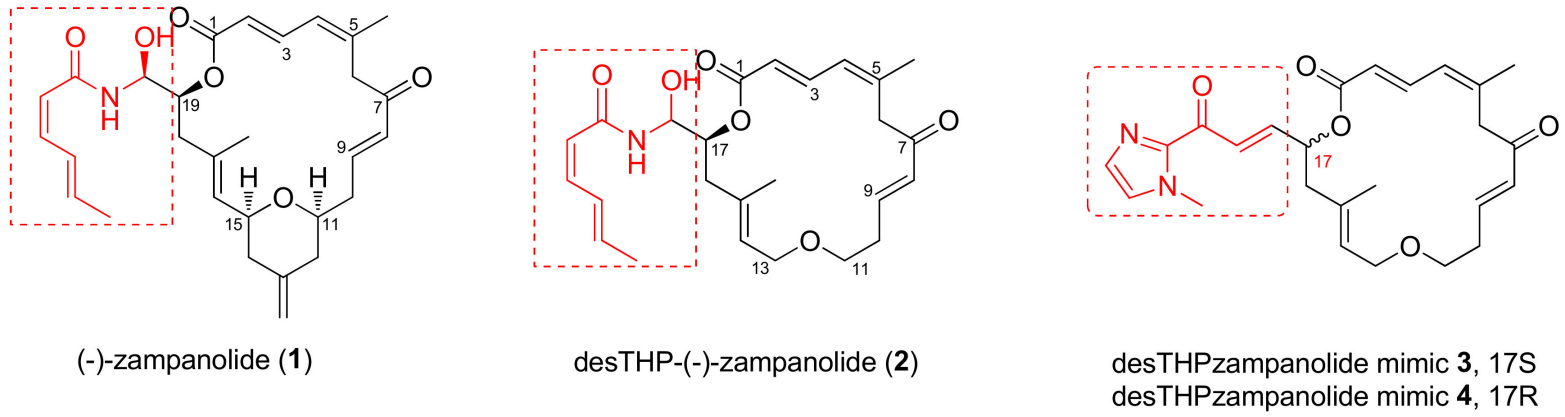
Zampanolide, desTHP-(−)-zampanolide, desTHPzampanolide mimics **3** and **4**.

The marine macrolide (−)-zampanolide (**1**, Figure 1) [11] has been demonstrated as a unique and promising MSA, which distinguishes it from the current clinically used MSAs due to (1) the covalent nature of its binding to the taxane pocket in *β*-tubulin as confirmed by a high-resolution X-ray structure of zampanolide in complex with *α*,*β*-tubulin, and (2) the very high cytotoxic potency as evidenced by low nanomolar IC_50_ values in suppressing multidrug-resistant cancer cell proliferation [12].

Although (−)-zampanolide has shown great potential in becoming a more effective chemotherapeutic for CRPC, no in vivo assessment of its antitumor efficacy, pharmacokinetics, and toxicity in animal models has so far been reported yet mainly because of its limited supply. Unfortunately, the supply issue of (−)-zampanolide cannot be addressed either by isolation from marine sponge sources or by current reported total syntheses. The best yield for isolation of (−)-zampanolide from marine sponge sources is merely 0.001% [12,13], and the highest yield for its syntheses is only 0.9% [14,15,16,17,18]. More importantly, the chemically fragile *N*-acyl hemiaminal side chain of zampanolide can compromise its pharmacokinetic profiles as well as the feasibility as a viable drug candidate. The easy cleavage of the *N*-acyl hemiaminal side chain has been confirmed by a thermolysis reaction carried out by Professor Smith and his coworkers [16], and the instability of zampanolide in CDCl_3_ has been reported by Higa and coworkers when they first identified its structure [13]. The *N*-acyl hemiaminal side chain of zampanolide is, however, very vital to retain its excellent cytotoxic potency, and the derivative without the side chain has 10- to 1000-fold less potency in certain cell models [12,19,20]. Our previous study has confirmed that a suitably designed side chain (e.g., the side chain in desTHPzampanoide mimics **3** and **4**) can function as a bioisostere for the chemically unstable *N*-acyl hemiaminal side chain in desTHPzampanolide (**2**) [21]. However, the bioisosteric side chains that we reported previously do not contain a hydroxyl substituent, corresponding to the C20 alcohol of zampanolide that is hydrogen-bonded to the carbonyl oxygen of Thr276 in β-tubulin. As part of our ongoing project to develop simplified and stabilized zampanolide mimics, this paper presents the design, total synthesis, and antiproliferative evaluation of zampanolide mimics in which the *N*-acyl hemiaminal side chain is substituted by more stable 2-fluorobenzyl alcohol appendages.

## 2. Results and Discussion

### 2.1. Design of New Zampanolide Mimics

**Figure 2:**
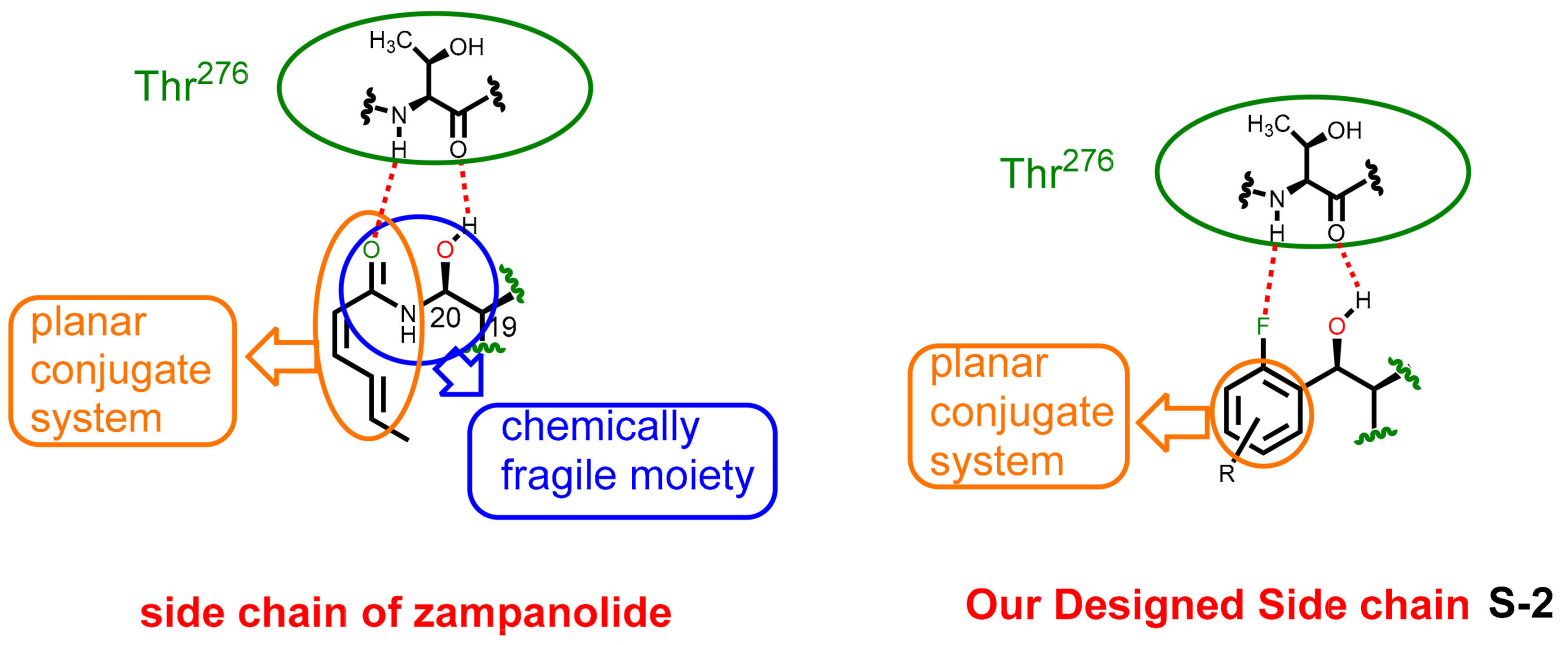
Design of a new stabilized side chain for zampanolide mimics.

The first group of side chains in the zampanolide mimics (e.g., **3**–**4**) previously published by us [21] do not contain a hydroxyl group in order to render a more simplified synthesis. However, the binding conformation confirmed by the high-resolution crystal structure of zampanolide tubulin complex [22] indicates the hydroxyl group at the C20 position in zampanolide is hydrogen-bonded to the carbonyl oxygen of Thr276 in β-tubulin. With this point in mind, a new side chain (S-2) was designed as a bioisosteric substitution for the N-acyl hemiaminal side chain, an unstable moiety in zampanolide but important for its potent cytotoxic properties. The stabilized side chain (S-2) was designed to replace the chemically fragile N-acylhemiaminal moiety (blue circle in Figure 2) with a benzyl alcohol. The 20OH group is retained in the target side chain so that it can be hydrogen-bonded to the main-chain carbonyl oxygen of Thr^276^ in β-tubulin. The 1′O atom of zampanolide’s side chain is replaced with fluorine atom in the designed side chain so that it can be hydrogen-bonded to the NH group of Thr^276^ in β-tubulin. The E,Z-hexadienoyl amide in zampanolide side chain is replaced with a planar conjugate phenyl ring to preserve the hydrophobic contacts between the proposed side chain and residues of β-tubulin. The Almann’s desTHP-dactylolide and its enantiomer [23] were further adopted as the core structure of the new zampanolide mimics (**5** and **6**) in the current study because of the acceptable in vitro potency of desTHP-(−)-zampanolide (**2**) and the practical synthesis of the core. Consequently, we set **5** and **6** as our new target mimics of zampanolide in this study.

### 2.2. Synthesis

**Scheme 1:**
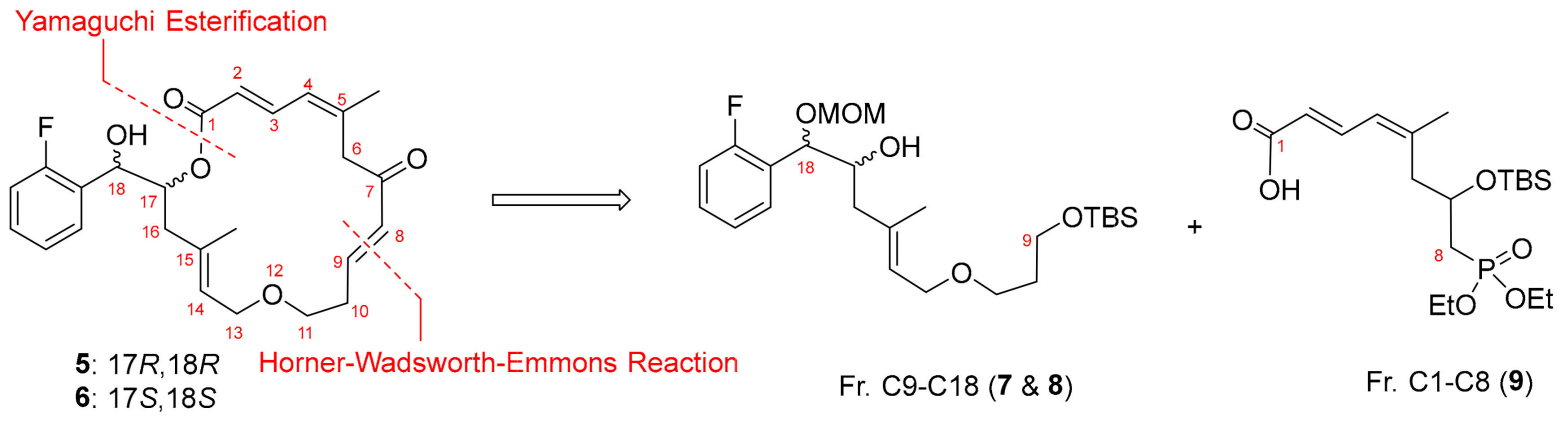
Retrosynthetic analysis of zampanolide mimics **5** and **6.**

Our retrosynthetic analysis of the zampanolide mimics **5** and **6**, using Yamaguchi esterification and intramolecular Horner–Wadsworth–Emmons condensation as key reactions, is illustrated in Scheme 1. The Yamaguchi esterification was employed to connect Fragment C1–C8 (carboxylic acid **9**) with Fragment C9–C18 (alcohol **7** or **8**) via the ester bond between C1 and C17. The intramolecular Horner–Wadsworth–Emmons condensation was used to close the macrolactone ring at C8–C9. These two key reactions were successfully applied to our syntheses of desTHPdactylolide and the mimics of desTHPzampanolides [21,23]. The known Fragment C1–C8 (**9**) was readily synthesized via 10 steps from commercially available 2-butyn-1-ol according to the reported procedure [24].

**Scheme 2:**
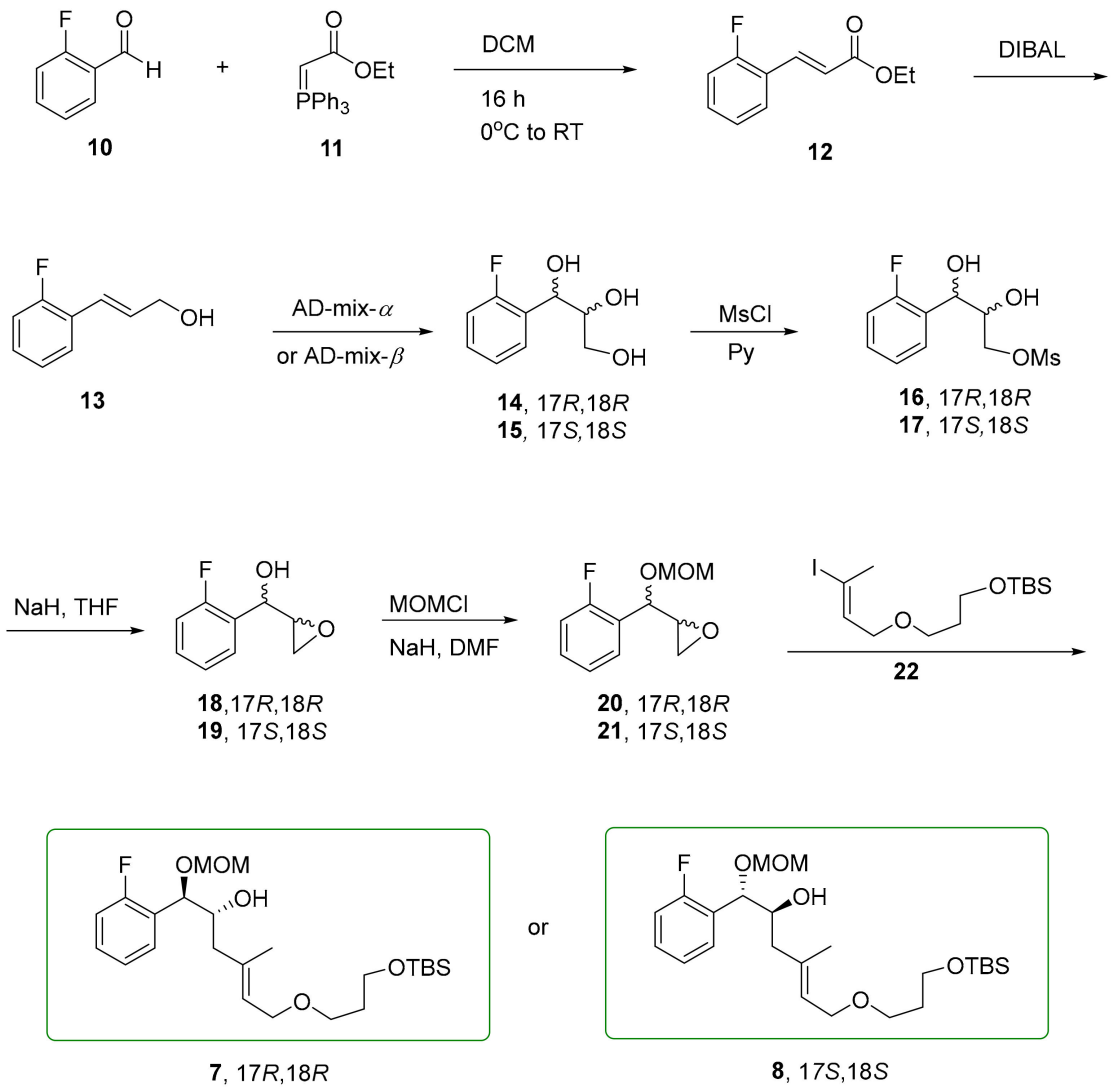
Synthesis of Fragment C9–C18 (**7** and **8**).

The preparation of Fragment C9–C18 (**7** and **8**) with the desired side chain is shown in Scheme 2. The chiral centers at C17 and C18 in the Fragment C9–C18 (**7** and **8**) were introduced by the Sharpless asymmetric dihydroxylation using the commercially available AD-mix formulations (AD-mix-*α* and AD-mix-*β*, respectively) with the ligand and the osmium salt as the critical trace component (0.6% by wt) and ferricyanide and carbonate as the bulk ingredient (99.4% by wt.) [25]. Compound (*E*)-ethyl 3-(2-fluorophenyl)acrylate was synthesized by the Wittig reaction of the commercially available 2-fluorobenzaldehyde (**10**) with ethyl 2-(triphenyl- phosphoranylidene)acetate (**11**) according to the procedure described in the literature [26]. (*E*)-3-(2-Fluorophenyl)prop-2-en-1-ol (**13**) was synthesized by reducing (*E*)-ethyl 3-(2-fluorophenyl)acrylate (**12**) with diisobutylaluminium hydride (DIBAL) based on the procedure reported in the literature [27]. The Sharpless asymmetric dihydroxylation of the allylic alcohol (**13**) with AD-mix-*α* or AD-mix-*β* was performed following the standard experimental procedure reported in the literature and sulfonamide was used to accelerate the hydrolysis of osmate ester [25]. Epoxy alcohols (**18** with *R*,*R* configuration, **19** with *S*,*S* configuration) have been synthesized by sequential selective mesylation of primary alcohol (**14** or **15**) and intermolecular Williamson ether synthesis [28]. The hydroxy group in epoxy alcohols (**18** with *R*,*R* configuration, **19** with *S*,*S* configuration) was then protected as the corresponding methoxymethyl (MOM) ethers (**20** with *R*,*R* configuration, **21** with *S*,*S* configuration). Iodo ether **22** was prepared using the optimized protocol previously developed in our laboratory [23]. Its lithiation with *tert*-butyllithium followed by nucleophilic reaction with epoxy MOM ether (**20** with *R*,*R* configuration, **21** with *S*,*S* configuration) in toluene at −90 °C mediated by boron trifluoride etherate furnished Fragment C9–C18 (**7** with *R*,*R* configuration, **8** with *S*,*S* configuration) in 22–23% overall yield from **22**.

**Scheme 3:**
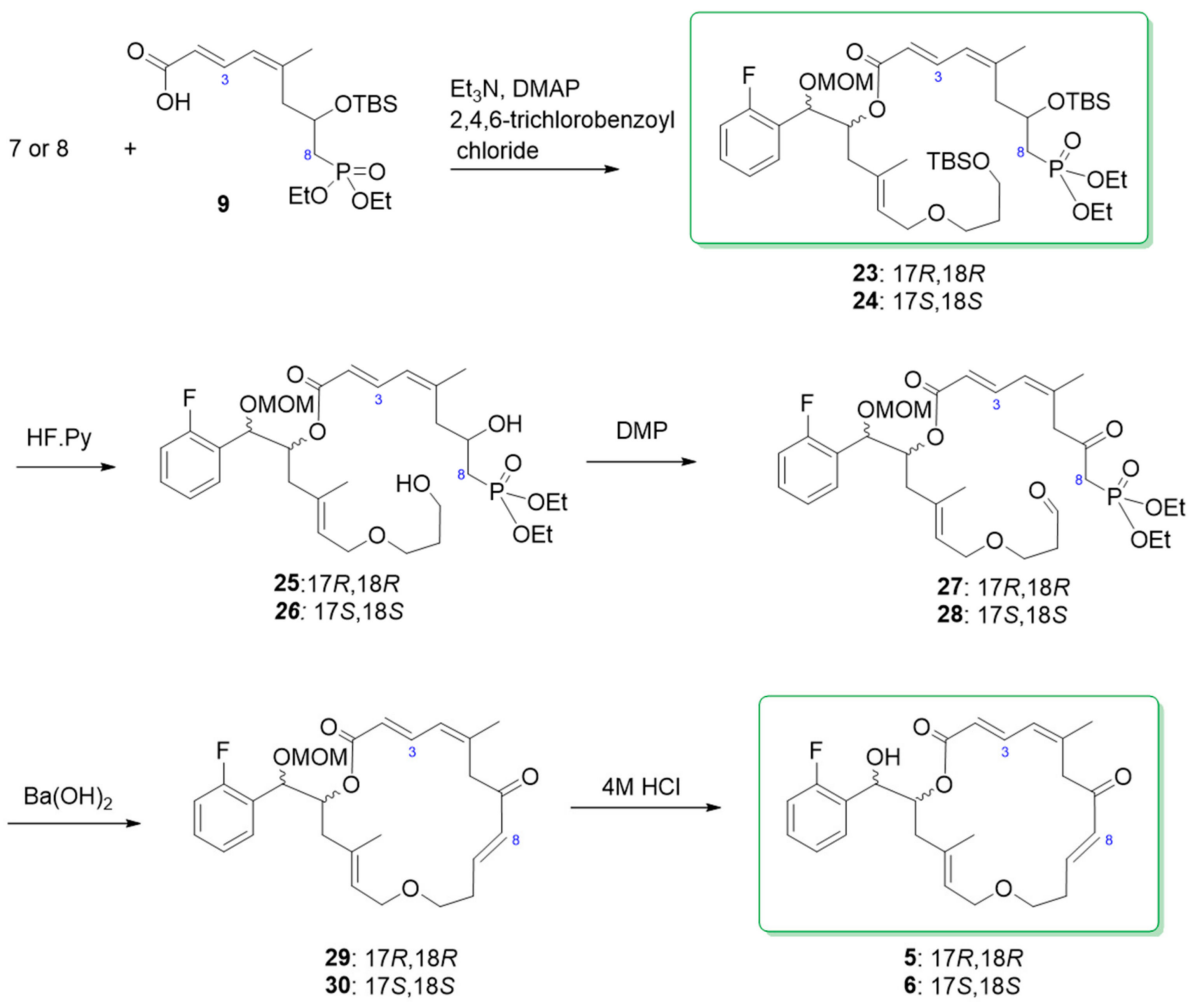
Synthesis of zampanolide mimics 5 and 6.

**Scheme 4:**
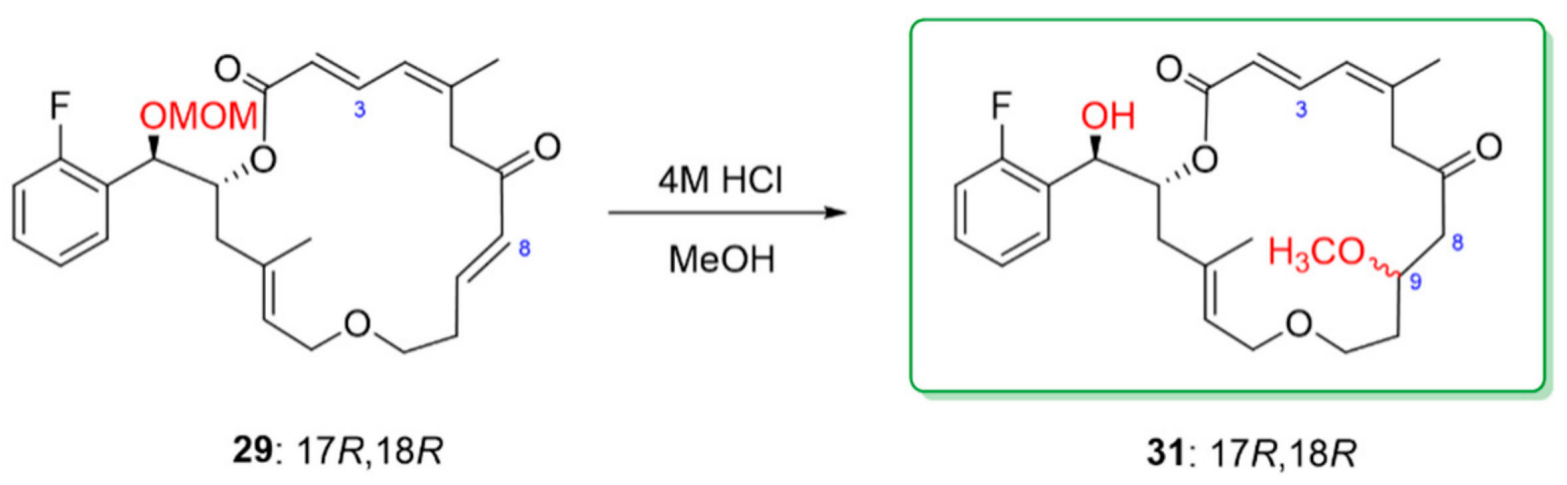
Deprotection of MOM ether 29 with methanolic HCl.

As illustrated in Scheme 3, the union of Fragment C1–C8 (alcohol **9**) and Fragment C9–C18 (carboxylic acid, **7** with *R*,*R* configuration, **8** with *S*,*S* configuration) has been accomplished by the Yamaguchi esterification to yield bis-TBS ethers (**23** with *R*,*R* configuration, **24** with *S*,*S* configuration). Removal of the protecting TBS groups at C7 and C9 with HF-pyridine complex followed by double oxidation of the corresponding diol (**25** with *R*,*R* configuration and **26** with *S*,*S* configuration) with Dess–Martin periodinane afforded the keto aldehydes (**27** with *R*,*R* configuration, **28** with *S*,*S* configuration). Without further chromatographic purification, the crude keto aldehydes were directly subjected to the synthesis of zampanolide mimics (**29** with *R*,*R* configuration, **30** with *S*,*S* configuration) through the intramolecular HWE condensation catalyzed by Ba(OH)_2_. Since extractive work-up resulted in significant hydrolysis of the ester [21], the reaction was quenched by filtration of the reaction mixture through a pad of silica gel and the crude product was subjected to preparative TLC purification. The MOM group in **29** was initially planned to be removed using methanolic HCl, but the methanol participated in a Michael addition to the enone, which resulted in the byproduct **31** (Scheme 4). The removal of the MOM with HCl was, therefore, carried out using THF rather than methanol as solvent, leading to the desired zampanolide mimics (**5** with *R*,*R* configuration at C17 and C18, **6** with *S*,*S* configuration at C17 and C18).

### 2.3. Antiproliferative Activity toward Prostate Cancer Cell Lines

**Figure 3:**
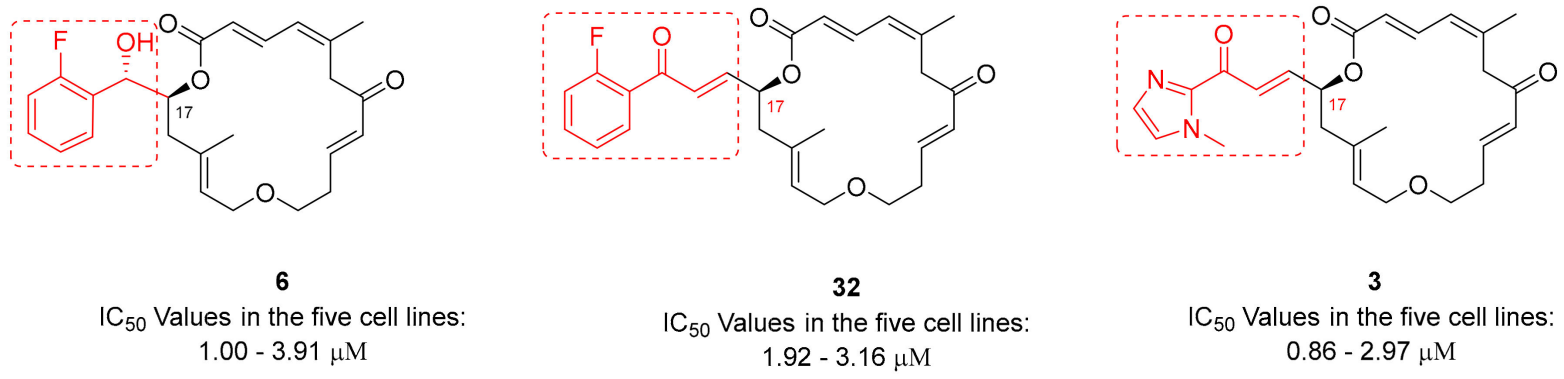
Comparison of antiproliferative potency among different zampanolide mimics.

The in vitro antiproliferative activity of two pairs of enantiomeric macrolides, **5** and **6**, **29** and **30**, has been assessed against a panel of prostate cancer cell lines, including three docetaxel-sensitive prostate cancer cell models (PC-3 DU145, and LNCaP) and two docetaxel-resistant prostate cancer cell lines (PC-3/DTX and DU145/DTX). WST-1 cell proliferation assay was used for the in vitro evaluation according to the procedure described in the Experimental Section. Docetaxel was used as a positive control while DMSO as a negative control. The half-maximal inhibitory concentrations (IC_50_ values) were measured by WST-1 cell proliferation assay after 3 days of exposure, calculated from the dose–response curves, and presented as the mean ± standard deviation of the mean in Table 1. The IC_50_ values for these four new zampanolide mimics are in the range of 1.00–9.36 µM. Mimic **6** has a set of smallest IC_50_ values (1.00–3.91 µM) among the four test macrolides. As summarized in Figure 3, the IC_50_ values of new mimic **6** are very close to those for mimic **3** (0.86–2.97 µM) and mimic **32** (1.92–3.16 µM). The mimic **3** has been evidenced to have similar potency as desTHPzampanolide **2** with the *N*-acetyl hemiaminal side chain of zampanolide against prostate cancer cells [21]. The side chain in mimic **6** was thus identified as a new bioisostere for the chemically unstable *N*-acetyl hemiaminal side chain of zampanolide. The relative resistance for Mimic **6** of PC-3/DTX over PC-3 and DU145/DTX over DU145 is 0.59 and 0.44, respectively. 

## 3. Materials and Methods

### 3.1. General Procedures

Optical rotations were measured on a RUDOLPH Research Analytical Autopol III Automatic Polarimeter (RUDOLPH Research Analytical, Hackettstown, NJ, USA). IR spectra were recorded on a Nicolet Nexus 470 FTIR spectrophotometer (Thermo Fisher Scientific, Waltham, MA, USA). High-Resolution MS were obtained on an Orbitrap mass spectrometer (Thermo Fisher Scientific, Waltham, MA, USA) with electrospray ionization (ESI). NMR spectra (see Supplementary Materials) were obtained on a Bruker Fourier 300 spectrometer (Billerica, MA, USA) in CDCl_3_. The chemical shifts are given in ppm referenced to the respective solvent peak, and coupling constants are reported in Hz. Anhydrous THF and dichloromethane were purified by PureSolv MD 7 Solvent Purification System from Innovative Technologies (MB-SPS-800) (Herndon, VA, USA). All other reagents and solvents were purchased from commercial sources (Fisher Scientific, Portland, OR, USA) and were used without further purification. Silica gel column chromatography was performed using silica gel (32–63 µM). Preparative thin-layer chromatography (PTLC) separations were carried out on thin-layer chromatography plates loaded with silica gel 60 GF254 (EMD Millipore Corporation, Berlington, MA, USA). Starting materials **22** and **9** were synthesized using the procedure previously described by us [23]. (*E*)-ethyl 3-(2-fluorophenyl)acrylate (**12**) was prepared from commercially available 2-fluorobenzaldehyde (**10**, CAS 446-52-6) according to the reported procedure [26]. (*E*)-3-(2-Fluorophenyl)prop-2-en-1-ol (**13**) was synthesized by reducing (*E*)-ethyl 3-(2-fluorophenyl)acrylate (**12**) with DIBAL based on the procedure reported in the literature [27]. Its structure was confirmed by ^1^H NMR and high resolution mass spectrometry (HRMS) data (calculated for C_9_H_10_FO (M + H): 153.0716; Found: 153.0710).

### 3.2. Synthesis of (1R,2R)-1-(2-Fluorophenyl)propane-1,2,3-triol [(17R,18R) triol ***14***]

AD-mix-*α* (5.9 g) was dissolved in a mixture of *tert*-butyl alcohol (21 mL) and water (21 mL). The solution was stirred vigorously at room temperature until two clear phases were observed. At this point, the lower aqueous phase emerged as bright yellow. Methanesulfonamide (399 mg, 4.2 mmol, 1 equiv) was added and the reaction mixture was cooled down to 0 °C. A solution of 13 (640 mg, 4.2 mmol, 1 equiv) in 1 mL of *tert*-butyl alcohol was then added. The resulting mixture was stirred vigorously at 0 °C overnight and the reaction progress was monitored with TLC. The reaction was quenched by adding solid sodium sulfate (6.3 g) at 0 °C. The mixture was warmed to room temperature and stirred for 30–60 min. The *tert*-butyl alcohol was removed under vacuum, and saturated sodium bicarbonate (30 mL) and water (300 mL) were added to the residue. The mixture was stirred for additional 30 min and then extracted with ethyl acetate (150 mL × 3). The combined extracts were rinsed with brine, dried over anhydrous sodium sulfate, and concentrated. The crude product was purified through column chromatography eluting with ethyl acetate. The product after purification, with quantitative yield, still contained a trace amount of methanesulfonamide, which is good enough to be used for the next-step reaction. ^1^H NMR (300 MHz, CDCl_3_) *δ*. 7.38 (t, *J* = 7.2 Hz, 1H, aromatic H), 7.21 (dd, *J* = 13.5, 5.7 Hz, 1H, aromatic H), 7.06 (t, *J* = 7.5 Hz, 1H, aromatic H), 6.95 (t, *J* = 8.4 Hz, 1H, aromatic H), 4.88 (d, *J* = 6.3 Hz, 1H, H-18), 4.23 (br.s, 3H, 3 × OH), 3.77 (q, *J* = 5.1 Hz, 1H, H-17), 3.45 (d, *J* = 4.8 Hz, 2H, H_2_-16). ^13^C NMR (75 MHz, CDCl_3_) *δ* 159.9 (d, *J*_CF_ = 243.8 Hz), 129.6, 128.5, 127.9 (d, *J*_CF_ = 12.8 Hz), 124.6, 115.4 (d, *J*_CF_ = 21.8 Hz), 75.3, 68.8, 63.5. IR (film) *ν*_max_: 3384, 2988, 1617, 1490, 1455, 1320, 1222, 1054 cm^−1^. HRMS (ESI): *m/z* calculated for C_9_H_12_FO_3_ [M + H]^+^: 187.0770. Found: 187.0766.

### 3.3. Synthesis of (2R,3R)-3-(2-Fluorophenyl)-2,3-dihydroxypropyl methanesulfonate [(17R,18R) mesylate ***16***]

To a solution of triol **14** (1.37 g, 7.39 mmol) in pyridine (15 mL, 0.5 M), methane sulfonyl chloride (0.57 mL, 7.39 mmol) was added dropwise at 0 °C under argon. The reaction mixture was stirred at room temperature for 20 h and the reaction progress was monitored by TLC (Hexane/EtOAc, 1:1, *v*/*v*). The reaction was quenched by diluting with ethyl acetate (800 mL), and the resulting solution was rinsed with brine (50 mL × 3) and dried over anhydrous sodium sulfate. After removing the organic solvent, the residue was subjected to column chromatography, using 40% ethyl acetate in hexane as eluent, to give the desired product. Colorless oil; 81% yield. ^1^H NMR (300 MHz, CDCl_3_) *δ* 7.47 (dt, *J* = 7.5, 1.8 Hz, 1H, aromatic H), 7.34–7.27 (m, 1H, aromatic H), 7.17 (dt, *J* = 7.5, 0.9 Hz, 1H, aromatic H), 7.04 (ddd, *J* = 10.5, 8.1, 0.9 Hz, 1H, aromatic H), 5.00 (d, *J* = 5.7 Hz, 1H, H-18), 4.27–4.16 (m, 2H, H_2_-16), 4.06–4.01 (m, 1H, H-17), 3.18 (br.s, 2H, 2 × OH), 3.03 (s, 3H, SO_2_*CH_3_*). ^13^C NMR (75 MHz, CDCl_3_) *δ* 159.9 (d, *J*_CF_ = 244.3 Hz), 130.1 (d, *J*_CF_ = 8.3 Hz), 128.4 (d, *J*_CF_ = 3.8 Hz), 127.0 (d, *J*_CF_ = 12.8 Hz), 124.8 (d, *J*_CF_ = 3.8 Hz), 115.7 (d, *J*_CF_ = 21.8 Hz), 72.9, 70.4, 67.9 (d, *J*_CF_ = 2.3 Hz), 37.6. IR (film) *ν*_max_: 3502, 3029, 2940, 1617, 1587, 1490, 1456, 1346, 1171 cm^−1^. HRMS (ESI): *m/z* calculated for C_10_H_14_FO_5_S [M + H]^+^: 265.0546. Found: 265.0547.

### 3.4. Synthesis of (R)-(2-Fluorophenyl)((R)-oxiran-2-yl)methanol [(17R,18R) epoxide ***18***]

To a suspension of sodium hydride (275 mg, 60%, 6.87 mmol, 1.5 equiv) in anhydrous THF (36 mL), a solution of **16** (1.21 g, 4.58 mmol, 1 equiv) in anhydrous THF was added at about −30 °C under argon. The reaction mixture was stirred at 0 °C overnight and the reaction progress was monitored with TLC (hexane:EtOAc, 3:1). The mixture was filtered through a silica gel pad eluting with ethyl acetate. After concentration to remove the solvent, the residue was subjected to PTLC purification eluting with toluene:EtOAc (3:1, *v*/*v*) to furnish the desired epoxide. Colorless oil; 57% yield. ^1^H NMR (300 MHz, CDCl_3_) *δ* 7.57 (dd, *J* = 7.8, 1.8 Hz, 1H, aromatic H), 7.35–7.28 (m, 1H, aromatic H), 7.20 (dt, *J* = 7.5, 1.2 Hz, 1H, aromatic H), 7.06 (ddd, *J* = 10.5, 8.1, 1.2 Hz, 1H, aromatic H), 4.84 (t, *J* = 6.6 Hz, 1H, H-18), 3.26–3.22 (m, 1H, H-17), 2.92–2.85 (overlapped, 2H, H_2_-16), 2.47 (d, *J* = 5.7 Hz, 1H, OH). ^13^C NMR (75 MHz, CDCl_3_) *δ* 159.9 (d, *J*_CF_ = 244.5 Hz), 129.6 (d, *J*_CF_ = 8.3 Hz), 127.9 (d, *J*_CF_ = 4.5 Hz), 127.3 (d, *J*_CF_ = 13.5 Hz), 124.5 (d, *J*_CF_ = 3.8 Hz), 115.4 (d, *J*_CF_ = 21.8 Hz), 68.7 (d, *J*_CF_ = 2.3 Hz), 55.6, 45.5 (d, *J* = 2.3 Hz). IR (film) *ν*_max_: 3420, 3067, 3002, 2928, 1617, 1586 1488, 1456, 1224 cm^−1^. HRMS (ESI): *m/z* calculated for C_9_H_10_FO_2_ [M + H]^+^: 169.0665. Found: 169.0657. 

### 3.5. Synthesis of (R)-2-((R)-(2-Fluorophenyl)(methoxymethoxy)methyl)oxirane [(17R,18R)MOM ether ***20***]

To a solution of alcohol **18** (716 mg, 4.3 mmol) in DMF (4.2 mL), sodium hydride (204 mg, 60%, 5.1 mmol) was added at 4 °C, and the mixture was stirred at 4 °C for 30 min. Methoxymethyl chloride (0.39 mL, 5.1 mmol) followed by tetrabutylammonium iodide (157 mg, 0.43 mmol) was added to the reaction mixture. The reaction was proceeded with stirring at room temperature for 20 h. The reaction mixture was diluted with ethyl acetate (300 mL) and diethyl ether (300 mL) and then rinsed with brine (50 mL × 3). The organic layer was dried over anhydrous sodium sulfate and concentrated to give a crude mass, which was purified by column chromatography eluting with hexane/ethyl acetate (3:1, *v*/*v*) to furnish the MOM ether **20** as a pale-yellow oil in 67% yield. ^1^H NMR (300 MHz, CDCl_3_) *δ* 7.51 (dt, *J* = 7.2, 1.8 Hz, 1H, aromatic H), 7.33–7.25 (m, 1H, aromatic H), 7.17 (dt, *J* = 7.5, 1.2 Hz, 1H, aromatic H), 7.04 (ddd, *J* = 9.9, 8.1, 1.2 Hz, 1H, aromatic H), 4.75 (d, *J* = 6.9 Hz, 1H, O*CH_2_*OCH_3_), 4.67 (d, *J* = 6.9 Hz, 1H, O*CH_2_*OCH_3_), 4.62 (d, *J* = 6.6 Hz, 1H, H-18), 3.36 (s, 3H, O*CH_3_*), 3.26–3.22 (m, 1H, H-17), 2.73–2.72 (overlapped, 2H, H_2_-16). ^13^C NMR (75 MHz, CDCl_3_) *δ* 160.3 (d, *J* = 244.5 Hz), 129.8 (d, *J* = 8.3 Hz), 128.6 (d, *J* = 4.5 Hz), 125.2 (d, *J* = 14.3 Hz), 124.4 (d, *J* = 3.0 Hz), 115.5 (d, *J* = 21.8 Hz), 94.6, 73.0, 55.6, 54.4, 44.2 (d, *J* = 2.3 Hz). IR (film) *ν*_max_: 2891, 1615, 1587, 1488, 1455, 1022 cm^−1^. HRMS (ESI): *m/z* calculated for C_11_H_14_FO_3_ [M + H]^+^: 213.0927. Found: 213.0921.

### 3.6. Synthesis of (5R,6R,E)-5-(2-Fluorophenyl)-8,16,16,17,17-pentamethyl-2,4,11,15-tetraoxa-16- silaoctadec-8-en-6-ol [(17R,18R) Fragment C9–C18 (***7***)]

To a solution of vinyliodide **22** (164 mg, 0.44 mmol; co-evaporated twice with pentane) in toluene (3 mL) at −78 °C, *tert*-butyllithium (0.46 mL, 1.9 M, 0.89 mmol) was added, and the mixture was stirred at −78 °C for 45 min prior to being cooled down to −90 °C. Epoxide **20** (188 mg, 0.89 mmol; co-evaporated twice with pentane) in toluene (1.5 mL) was added dropwise to make sure that interior temperature lower than −78 °C. The reaction solution was re-cooled to −90 °C before BF_3_•OEt_2_ (0.11 mL, 0.89 mmoL) was added dropwise. The resulting solution was then stirred at −78 °C overnight prior to being quenched with ethyl acetate (15 mL) and saturated aqueous sodium bicarbonate (35 mL) was added. The organic layer was separated and the aqueous layer was extracted with ethyl acetate (60 mL × 3). The combined organic layers were dried over anhydrous sodium sulfate and concentrated *in Vacuum* to remove ethyl acetate. The crude mass was subjected to PTLC purification over silica gel using hexane/ethyl acetate (4:1, *v*/*v*) as eluent to yield secondary alcohol **7** as a pale-yellow oil in 23% yield. ^1^H NMR (300 MHz, CDCl_3_) *δ*. 7.41 (dt, *J* = 7.2, 1.5 Hz, 1H, aromatic H), 7.32–7.24 (m, 1H, aromatic H), 7.15 (dt, *J* = 7.5, 0.9 Hz, 1H, aromatic H), 7.53 (ddd, *J* = 9.9, 8.1, 0.9 Hz, 1H, aromatic H), 5.41 (t, *J* = 6.6 Hz, 1H, H-14), 4.81 (d, *J* = 6.3 Hz, 1H, H-17), 4.62 (d, *J* = 6.9 Hz, 1H, O*CH_2_*OCH_3_), 4.58 (d, *J* = 6.6 Hz, 1H, O*CH_2_*OCH_3_), 3.94 (d, *J* = 6.6 Hz, 2H, H_2_-13), 3.67 (t, *J* = 6.3 Hz, 2H, H_2_-11), 3.46 (t, *J* = 6.3 Hz, 2H, H_2_-9), 3.36 (s, 3H), O*CH_3_*), 1.75 (quin, *J* = 6.3 Hz, 2H, H_2_-10), 1.64 (s, 3H, 15-*CH_3_*), 0.87 (s, 9H, TBS), 0.03 (s, 6H, TBS). ^13^C NMR (75 MHz, CDCl_3_) *δ* 160.9 (d, *J*_CF_ = 244.8 Hz), 136.4, 129.8, 129.1, 128.7, 126.1 (d, *J*_CF_ = 13.5 Hz), 124.5 (d, *J*_CF_ = 3.8 Hz), 115.6 (d, *J*_CF_ = 21.8 Hz), 95.3, 75.6, 72.5 (d, *J*_CF_ = 12.1 Hz), 67.3, 67.0, 60.2, 56.2, 43.0, 33.2, 26.1, 26.07, 18.5, 16.6, 16.5. IR (film) *ν*_max_: 3420, 2929, 1715, 1647, 1615, 1488, 1029 cm^−1^. HRMS (ESI): *m/z* calculated for C_24_H_42_FO_5_Si [M + H]^+^: 457.2786. Found: 457.2780.

### 3.7. Synthesis of (2E,4Z)-(5R,6R,E)-5-(2-Fluorophenyl)-8,16,16,17,17-pentamethyl-2,4,11,15-tetraoxa-16-silaoctadec-8-en-6-yl 7-((tert-butyldimethylsilyl)oxy)-8-(diethoxyphosphoryl)-5- methylocta-2,4-dienoate [(17R,18R) ester ***23***]

To a solution of Fragment C1–C8 (**9**, 37 mg, 0.09 mmol; co-evaporated with pentane twice) in toluene (0.5 mL) at room temperature, trimethylamine (28 µL, 0.20 mmol) and 2,4,6-trichlorobenzoyl chloride (18 µL, 0.12 mmol) was sequentially added, and the reaction mixture was stirred at room temperature for 1.5 h. A solution of alcohol **7** (35 mg, 0.08 mmol) and DMAP (9.3 mg, 0.08 mmol) in toluene (0.5 mL) was then added to the reaction mixture. The reaction was allowed to proceed with stirring at room temperature for 19 h prior to being quenched with water (5 mL) and saturated aqueous sodium bicarbonate (5 mL). The mixture was extracted with ethyl acetate (5 mL × 3), the combined organic extracts were dried over anhydrous sodium sulfate and concentrated *in vacuum*. PTLC purification of the crude product over silica gel eluting with hexane/ethyl acetate (60:40, *v*/*v*) yielded the ester **23** as a mixture of diastereoisomers in a 1:1 ratio as a colorless oil in 77% yield. ^1^H NMR (300 MHz, CDCl_3_) *δ* 7.50 (dd, *J* = 15.0, 11.7 Hz, H-3), 7.42 (t, *J* = 7.5 Hz, 1H, aromatic H), 7.25–7.20 (m, 1H, aromatic H), 7.10 (t, *J* = 7.5 Hz, 1H, aromatic H), 7.00 (t, *J* = 8.4 Hz, 1H, aromatic H), 6.03 (d, *J* = 11.4 Hz, H-4), 5.75 (d, *J* = 15.3 Hz, 1H, H-2), 5.43–5.37 (m, 1H, H-17), 5.33 (t, *J* = 6.6 Hz, 1H, H-14), 5.08 (d, *J* = 5.4 Hz, 1H, H-18), 4.58 (d, *J* = 6.6 Hz, 1H, -O*CH_2_*OCH_3_), 4.51 (d, *J* = 6.6 Hz, 1H, O*CH_2_*OCH_3_), 4.18–4.04 (overlapped, 5H, 2 x O*CH*_2_CH_3_, H-7), 3.93–3.79 (m, 2H, H_2_-13), 3.64 (t, *J* = 6.3 Hz, 2H, H_2_-11), 3.40 (t, *J* = 6.3 Hz, 2H, H_2_-9), 3.32 (s, 3H, -OCH_2_O*CH_3_*), 2.58–2.48 (m, 2H, H_2_-16), 2.33–2.24 (m, 2H, H_2_-8), 2.00–1.92 (m, 2H, H_2_-6), 1.87 (s, 3H, 5-*CH_3_*), 1.72 (quin, *J* = 6.3 Hz, 2H, H_2_-10), 1.64 (1.63) (s, 3H, 15-*CH_3_*), 1.31 (t, *J* = 7.2 Hz, 6H, 2 × *O*CH_2_*CH_3_*), 0.86 (s, 9H, TBS), 0.81 (0.80) (s, 9H, TBS), 0.04 (0.03) (s, 3H, TBS), 0.01 (s, 6H, TBS), −0.04(−0.05) (s, 3H, TBS). ^13^C NMR (75 MHz, CDCl_3_) *δ* 166.8 (166.7), 160.8 (d, *J* = 244.5 Hz), 146.4 (d, *J* = 9.8 Hz), 141.6, 135.2 (d, *J* = 3.8 Hz), 129.7, 129.0, 126.8, 125.7 (d, *J* = 13.5 Hz), 125.1, 124.3, 119.3, 115.6 (115.3), 95.0, 72.9, 72.3, 67.3, 66.8, 61.7(61.6), 60.5, 60.1, 56.1, 41.8 (41.2), 36.1, 34.3, 33.1, 27.0, 26.11, 26.05, 25.90, 25.87, 25.2, 25.0, 18.5, 18.0, 16.6 (16.5), −4.6, −5.2. IR (film) *ν*_max_: 2929, 2856, 1716, 1489, 1251, 1020 cm^−1^. HRMS (ESI): *m/z* calculated for C_43_H_77_FO_10_PSi_2_ [M + H]^+^: 859.4777. Found: 859.4756. 

### 3.8. Synthesis of (2E,4Z)-(1R,2R,E)-1-(2-Fluorophenyl)-6-(3-hydroxypropoxy)-1-(methoxymethoxy) -4-methylhex-4-en-2-yl 8-(diethoxyphosphoryl)-7-hydroxy-5-methylocta-2,4-dienoate [(17R,18R) diol ***25***]

To a solution of di-TBS ether **23** (90 mg, 0.105 mmol) in THF (4.2 mL) at 0 °C in a plastic bottle, hydrogen fluoride-pyridine complex (70%, 1.0 mL) was added dropwise. The solution was stirred at 0 °C for 5 min and then at room temperature for 16 h. Saturated sodium bicarbonate (30 mL) was added to quench the reaction and the suspension was extracted with ethyl acetate (30 mL × 3). The combined organic extracts were dried over anhydrous sodium sulfate and the dried extract was concentrated under vacuum. The crude product was purified by PTLC, eluting with dichloromethane/methanol (93:7. *v*/*v*), and the pure diol was washed out from the PTLC silica gel with acetone as a pale-yellow oil in 92% yield. ^1^H NMR (300 MHz, CDCl_3_) *δ* 7.50 (7.49) (dd, *J* = 15.3, 11.7 Hz, 1H, H-3), 7.43 (t, *J* = 9.0 Hz, 1H, aromatic H), 7.30–7.23 (m, 1H, aromatic H), 7.13 (7.12) (t, *J* = 7.5 Hz, 1H, aromatic H), 7.031 (7.028) (dd, *J* = 10.2, 9.0 Hz, 1H, aromatic H), 6.11 (d, *J* = 11.4 Hz, 1H, H-4), 5.80 (d, *J* = 15.3 Hz, 1H, H-2), 5.47–5.40 (m, 1H, H-17), 5.32 (t, *J* = 5.7 Hz, 1H, H-14), 5.09 (d, *J* = 5.7 Hz, 1H, H-18), 4.58 (d, *J* = 6.9 Hz, 1H, -O*CH_2_*OCH_3_), 4.51 (d, *J* = 6.9 Hz, 1H, O*CH_2_*OCH_3_), 4.18–4.06 (overlapped, 5H, 2 × O*CH_2_*CH_3_, H-7), 3.90 (t, *J* = 5.7 Hz, 2H, H_2_-13), 3.69 (3.68) (t, *J* = 5.7 Hz, 2H, H_2_-11), 3.48 (3.47) (t, *J* = 5.7 Hz, 2H, H_2_-9), 3.33 (3.32) (s, 3H, -OCH_2_O*CH_3_*), 3.03 (s, 2H, 2 × OH), 2.66–2.53 (m, 1H, H-16), 2.46–2.19 (m, 3H, H_2_-8 & H-16), 1.92 (S, 3H, 5-*CH_3_*), 1.96–1.88 (m, 2H, H_2_-6), 1.75 (quin, *J* = 5.7 Hz, 2H, H_2_-10), 1.65 (s, 3H, 15-*CH_3_*), 1.33 (t, *J* = 7.2 Hz, 6H, 2 × OCH_2_*CH_3_*). ^13^C NMR (75 MHz, CDCl_3_) *δ* 167.0 (166.8), 160.9 (d, *J* = 247.5 Hz), 146.0, 140.8 (d, *J* = 17.3 Hz), 135.7 (d, *J* = 9.0 Hz), 129.8, 129.0, 126.8, 125.6 (d, *J* = 12.8 Hz), 124.8 (d, *J* = 11.3 Hz), 124.4, 119.7 (d, *J* = 11.3 Hz), 115.7 (115.4), 94.9, 73.0, 72.4, 68.8, 67.4, 65.3, 62.2, 61.8, 56.1, 41.1, 34.6 (34.5), 32.7, 32.3, 25.3, 25.0, 16.6 (16.5). IR (film) *ν*_max_: 3366, 2929, 1711, 1633, 1488, 1222, 1019 cm^−1^. HRMS (ESI): *m/z* calculated for C_31_H_49_FO_10_P [M + H]^+^: 631.3048. Found: 631.3041.

### 3.9. Synthesis of (R,3E,9E,11Z,15E)-6-((R)-(2-Fluorophenyl)(methoxymethoxy)methyl)-4,12- dimethyl-1,7-dioxacyclooctadeca-3,9,11,15-tetraene-8,14-dione [(17R,18R) macrolactone ***29***]

To a solution of diol **25** (65 mg, 0.10 mmol) in dichloromethane (6.2 mL) at room temperature, Dess–Martin periodinane (DMP, 131 mg, 0.30 mmol) was added. The reaction mixture was stirred for 30 min before a second portion of DMP (131 mg, 0.30 mmol) was added. The reaction was allowed to proceed with stirring at room temperature for an additional 1 h. The reaction mixture was poured into a stirred mixture of saturated sodium bicarbonate (30 mL) and saturated sodium thiosulfate (30 mL), and the resulting suspension was stirred for 30 min before being extracted with dichloromethane (30 mL × 3). The combined organic extracts were dried over anhydrous sodium sulfate and the dried extract was concentrated under vacuum. The crude product was used for the next reaction without further purification. To a solution of the crude ketoaldehyde (0.10 mmol) obtained above in THF (103 mL) at 0 °C was added water (2.2 mL) followed by activated barium hydroxide (15 mg, 0.08 mmol). The reaction mixture was stirred at 0 °C for 30 min and at room temperature for 2.5 h, then was filtered through a pad of sodium sulfate and a pad of silica gel that was rinsed with ethyl acetate. After evaporation of the solvent, the crude product was subjected to PTLC purification, using hexane/ethyl acetate (73:37) as eluent, to yield the pure macrolactone **29**. Colorless oil, 46% yield for two steps. [α]: −15.6 (*c* = 0.23, MeOH). ^1^H NMR (300 MHz, CDCl_3_) *δ* 7.60 (dd, *J* = 15.0, 11.4 Hz, 1H, H-3), 7.46 (dt, *J* = 7.5, 1.8 Hz, 1H, aromatic H), 7.33–7.25 (m, 1H, aromatic H), 7.16 (dt, *J* = 7.5, 1.2 Hz, 1H, aromatic H), 7.05 (ddd, *J* = 9.9, 8.4, 1.2 Hz, 1H, aromatic H), 6.82 (dt, *J* = 16.2, 6.6 Hz, 1H, H-9), 6.12 (d, *J* = 11.4 Hz, 1H, H-4), 6.01 (d, *J* = 16.2 Hz, 1H, H-8), 5.91 (d, *J* = 15.0 Hz, 1H, H-2), 5.51 (ddd, *J* = 11.4, 6.3, 1.2 Hz, 1H, H-17), 5.27 (dd, *J* = 7.8, 4.8 Hz, 1H, H-14), 5.12 (d, *J* = 6.3 Hz, 1H, H-18), 4.60 (d, *J* = 6.9 Hz, 1H, *O*-*CH_2_*OCH_3_), 4.53 (d, *J* = 6.9 Hz, 1H, O*CH_2_*OCH_3_), 3.99 (dd, *J* = 12.0, 8.1 Hz, 1H, H-16), 3.88–3.81 (m, 2H, H_2_-13), 3.47–3.28 (m, 2H, H_2_-11), 3.34 (s, 3H, OCH_2_O*CH_3_*), 3.14 (d, *J* = 12.6 Hz, 1H, H-16), 2.43–2.36 (m, 2H, H_2_-10), 2.33 (d, *J* = 12.0 Hz, 1H, H-6), 2.05 (d, *J* = 12.1 Hz, 1H, H-6), 1.83 (s, 3H, 5-CH_3_), 1.63 (S, 3H, 15-CH_3_). ^13^C NMR (75 MHz, CDCl_3_) *δ* 197.2, 166.6, 160.9 (d, *J* = 244.5 Hz), 146.8, 142.5, 139.7, 138.9, 134.3, 130.3, 128.9 125.9 (d, *J* = 7.5 Hz), 125.6 (d, *J* = 13.5 Hz), 125.2 (d, *J* = 5.6 Hz), 124.5, 120.9 (d, *J* = 5.3 Hz), 115.6 (d, *J* = 21.8 Hz), 94.9, 72.9, 72.6, 67.9, 67.8, 56.0, 46.0, 41.4, 33.1, 24.09, 16.7. IR (film) *ν*_max_: 2931, 1713, 1633, 1489, 1358, 1226, 1030 cm^−1^. HRMS (ESI): *m/z* calculated for C_27_H_34_FO_6_ [M + H]^+^: 473.2339. Found: 473.2333.

### 3.10. Synthesis of (R,3E,9E,11Z,15E)-6-((R)-(2-Fluorophenyl)(hydroxy)methyl)-4,12-dimethyl- 1,7-dioxacyclooctadeca-3,9,11,15-tetraene-8,14-dione [(17R,18R) macrolactone ***5***]

To a solution of **29** (30 mg, 0.064 mmol) in a mixture of tetrahydrofuran/water (0.92 mL, 1:1, *v*/*v*), concentrated hydrochloric acid (130 µL) was added. The two more portions of concentrated hydrochloric acid (130 µL × 2) were added sequentially after each one-hour stirring at room temperature. The reaction mixture was stirred at room temperature overnight before the reaction was quenched by adding saturated ammonium chloride (60 mL). The subsequent mixture was extracted with ethyl acetate (40 mL × 3). The combined extracts were dried over anhydrous sodium sulfate and the solvents were evaporated *in vacuum*. The crude product was purified over preparative thin layer chromatography eluting with toluene/ethyl acetate (3:1, *v*/*v*) to furnish the desired product (11 mg). Colorless syrup, 41% yield. ^1^H NMR (300 MHz, CDCl_3_) *δ* 7.62 (dd, *J* = 15.0, 11.7 Hz, 1H, H-3), 7.49 (dt, *J* = 7.5, 1.8 Hz, 1H, aromatic H), 7.31–7.27 (m, 1H, aromatic H), 7.17 (dt, *J* = 7.5, 1.2 Hz, 1H), 7.05 (ddd, *J* = 10.2, 8.1, 0.9 Hz, 1H), 6.83 (dt, *J* = 15.9, 6.9 Hz, 1H, H-9), 6.12 (d, *J* = 11.4 Hz, 1H, H-4), 6.02 (d, *J* = 16.2 Hz, 1H, H-8), 5.91 (d, *J* = 15.0 Hz, 1H, H-2), 5.48–5.38 (m, 1H, H-17), 5.27 (t, *J* = 6.0 Hz, 1H, H-14), 5.10 (d, *J* = 6.3 Hz, 1H, H-18), 3.99 (dd, *J* = 12.0, 8.1 Hz, 1H, H-16), 3.86 (br.s, 1H, H-13), 3.82 (br.s, 1H, H-13), 3.51–3.36 (m, 2H, H_2_-11), 3.18 (d, *J* = 12.9 Hz, 1H, H-16), 2.44–2.34 (m, 3H, H_2_-10, H-6), 2.06 (d, *J* = 14.4 Hz, 1H, H-6), 1.83 (s, 3H, 5-*CH_3_*), 1.62 (s, 3H, 15-*CH_3_*). ^13^C NMR (75 MHz, CDCl_3_) *δ* 197.2, 167.0, 160.1 (d, *J*_CF_ = 244.5 Hz), 146.9, 143.0, 140.1 (d, *J*_CF_ = 12.8 Hz), 134.2, 130.2, 129.9, 128.8, 127.6 (d, *J*_CF_ = 12.8 Hz), 126.0, 125.2, 124.6, 120.7, 115.6 (d, *J*_CF_ = 21.8 Hz), 79.4, 74.4, 70.3, 68.0, 45.9, 41.4, 33.0, 24.1, 16.7. [α]: −15.2 (*c* = 0.16, MeOH). IR (film) *ν*_max_: 3428, 2924, 2854, 1706, 1633, 1558, 1488, 1360, 1221, 1036 cm^−1^. HRMS (ESI): *m/z* calculated for C_25_H_30_FO_5_ [M + H]^+^: 429.2077. Found: 429.2074.

### 3.11. Synthesis of (1S,2S)-1-(2-Fluorophenyl)propane-1,2,3-triol [(17S,18S) triol ***15***]

Triol **15** was prepared in 68% yield from 13 catalyzed by AD-mix-*β* using a similar procedure used to synthesize its antipode **14**. ^1^H NMR (300 MHz, CDCl_3_) *δ* 7.39 (t, *J* = 7.5 Hz, 1H, aromatic H), 7.22 (dd, *J* = 13.8, 7.2 Hz, 1H, aromatic H), 7.07 (t, *J* = 7.5 Hz, 1H, aromatic H), 6.96 (t, *J* = 9.3 Hz, 1H, aromatic H), 4.90 (d, *J* = 6.3 Hz, 1H, H-18), 4.21 (br.s, 3H, 3 × OH), 3.78 (dd, *J* = 10.2, 4.8 Hz, 1H, H-17), 3.48 (d, *J* = 4.8 Hz, 2H, H_2_-16). ^13^C NMR (75 MHz, CDCl_3_) *δ* 160.0 (d, *J*_CF_ = 244.5 Hz), 129.6, 128.5 (d, *J*_CF_ = 16.5 Hz), 127.8 (d, *J*_CF_ = 12.8 Hz), 124.6, 115.5 (d, *J*_CF_ = 21.8 Hz), 75.4, 68.8, 63.4. IR (film) *ν*_max_: 3375, 2930, 1781, 1489, 1456, 1319 cm^−1^. HRMS (ESI): *m/z* calculated for C_9_H_12_FO_3_ [M + H]^+^: 187.0770. Found: 187.0765.

### 3.12. Synthesis of (2S,3S)-3-(2-Fluorophenyl)-2,3-dihydroxypropyl methanesulfonate [(17S,18S) mesylate ***17***]

Mesylate **17** was prepared from triol **15** as a colorless oil in 72% yield, employing a procedure similar to that used for the mesylation of triol **14**. ^1^H NMR (300 MHz, CDCl_3_) *δ* 7.48 (dt, *J* = 7.5, 1.8 Hz, 1H, Aromatic H), 7.32–7.29 (m, 1H, aromatic H), 7.18 (dt, *J* = 8.4, 0.9 Hz, 1H, aromatic H), 7.05 (ddd, *J* = 9.3, 8.1, 0.9 Hz, 1H, aromatic H), 5.01 (d, *J* = 5.7 Hz, 1H, H-18), 4.28–4.15 (m, 2H, H_2_-16), 4.08–4.03 (m, 1H, H-17), 3.04 (s, 3H, SO_2_*CH_3_*). ^13^C NMR (75 MHz, CDCl_3_) *δ* 159.9 (d, *J*_CF_ = 244.5 Hz, 1H), 130.1, 128.4, 127.0 (d, *J*_CF_ = 12.8 Hz, 1H), 124.8, 115.7 (d, *J*_CF_ = 21.8 Hz, 1H), 72.9, 70.3, 67.9, 37.6. IR (film) *ν*_max_: 3482, 3029, 2939, 1616, 1587, 1489, 1456, 1332, 1168 cm^−1^. HRMS (ESI): *m/z* calculated for C_10_H_14_FO_5_S [M + H]^+^: 265.0546. Found: 265.0542.

### 3.13. Synthesis of (S)-(2-Fluorophenyl)((S)-oxiran-2-yl)methanol [(17S,18S) epoxide ***19***]

Epoxide **19** (52%, pale yellow oil) was obtained from mesylate **17** according to the internal Williamson ether synthesis procedure employed for the conversion of mesylate **16** to epoxide **18**. ^1^H NMR (300 MHz, CDCl_3_) *δ* 7.57 (dt, *J* = 7.5, 1.8 Hz, 1H, aromatic H), 7.35–7.25 (m, 1H, aromatic H), 7.20 (dt, *J* = 7.5, 1.2 Hz, 1H, aromatic H), 7.06 (ddd, *J* = 10.5, 8.1, 1.2 Hz, 1H, aromatic H), 4.83 (d, *J* = 5.1 Hz, 1H, H-18), 3.26–3.21 (m, 1H, H-17), 2.92–2.84 (m, 2H, H_2_-16). ^13^C NMR (75 MHz, CDCl_3_) *δ* 160.0 (d, *J*_CF_ = 244.5 Hz), 129.8, 127.6 (d, *J*_CF_ = 9.0 Hz), 127.4 (d, *J*_CF_ = 13.5 Hz), 124.7, 115.6 (d, *J*_CF_ = 21.0 Hz), 68.6, 55.4, 45.6. IR (film) *ν*_max_: 3482, 3029, 2939, 1617, 1587, 1489, 1456, 1331, 1168 cm^−1^. HRMS (ESI): *m/z* calculated for C_9_H_10_FO_2_ [M + H]^+^: 169.0665. Found: 169.0659.

### 3.14. Synthesis of (S)-2-((S)-(2-Fluorophenyl)(methoxymethoxy)methyl)oxirane [(17S,18S) MOM ether ***21***]

MOM ether **21** was prepared from epoxide **19** as a pale-yellow oil in 73% yield employing a procedure similar to that used for conversion of **18**. ^1^H NMR (300 MHz, CDCl_3_) *δ* 7.44 (dt, *J* = 7.5, 1.8 Hz, 1H, aromatic H), 7.32–7.27 (m, 1H, aromatic H), 7.17 (dt, *J* = 7.5, 0.9 Hz, 1H, aromatic H), 7.05 (ddd, *J* = 10.2, 8.4, 1.2 Hz, 1H, aromatic H), 4.76 (d, *J* = 6.6 Hz, 1H, -O*CH_2_*OCH_3_), 4.68 (d, *J* = 6.9 Hz, 1H, H-18), 4.63 (d, *J* = 6.6 Hz, 1H, -O*CH_2_*OCH_3_), 3.37 (s, 3H, -OCH_2_O*CH_3_*), 3.27 – 3.23 (m, 1H, H-17), 2.75–2.73 (m, 2H, H_2_-16). ^13^C NMR (75 MHz, CDCl_3_) *δ* 160 (d, *J*_CF_ = 245.3 Hz), 129.9 (d, *J*_CF_ = 8.3 Hz), 128.7 (d, *J*_CF_ = 3.8 Hz), 125.3 (d, *J*_CF_ = 14.3 Hz), 124.5 (d, *J*_CF_ = 3.8 Hz), 115.7 (d, *J*_CF_ = 21.8 Hz), 94.8, 73.1, 55.8, 54.6, 44.4. IR (film) *ν*_max_: 2891, 2825, 1616, 1587, 1488, 1455, 1149, 1022 cm^−1^. HRMS (ESI): *m/z* calculated for C_11_H_14_FO_3_ [M + H]^+^: 213.0927. Found: 213.0920.

### 3.15. Synthesis of (5S,6S,E)-5-(2-Fluorophenyl)-8,16,16,17,17-pentamethyl-2,4,11,15-tetraoxa-16- silaoctadec-8-en-6-ol [(17S, 18S) Fragment C9–C18 (***8***)]

(17*S*,18*S*) Fragment C9–C18 (**8**) was synthesized as a colorless oil in 22% yield from MOM ether **21** in a similar way to the synthesis of its antipode (17*R*,18*R*) Fragment C9–C18 (7). ^1^H NMR (300 MHz, CDCl_3_) *δ* 7.41 (dt, *J* = 7.5, 1.8 Hz, 1H, aromatic H), 7.32–7.24 (m, 1H, aromatic H), 7.15 (dt, *J* = 7.5, 1.2 Hz, 1H, aromatic H), 7.03 (ddd, *J* = 10.2, 8.4, 1.2 Hz, 1H, aromatic H), 5.41 (t, *J* = 6.6 Hz, 1H, H-14), 4.81 (d, *J* = 6.6 Hz, 1H, H-18), 4.63 (d, *J* = 6.6 Hz, 1H, O*CH_2_*OCH_3_), 4.58 (d, *J* = 6.6 Hz, 1H, O*CH_2_*OCH_3_), 3.95 (d, *J* = 6.6 Hz, 2H, H_2_-13), 3.82 (dt, *J* = 10.2, 5.7 Hz, 1H, H-17), 3.67 (t, *J* = 6.0 Hz, 2H, H_2_-11), 3.46 (t, *J* = 6.6 Hz, 2H, H_2_-9), 3.36 (s, 3H, OCH_3_), 2.23 (dd, *J* = 12.6, 9.6 Hz, 1H, H-16), 2.05 (dd, *J* = 12.6, 2.7 Hz, 1H, H-16), 1.76 (quin, *J* = 6.3 Hz, 2H, H_2_-10), 1.64 (s, 3H, 15-CH_3_), 0.87 (s, 9H, TBS), 0.03 (s, 6H, TBS). ^13^C NMR (75 MHz, CDCl_3_) *δ* 160.9 (d, *J*_CF_ = 244.7 Hz), 136.4, 129.2, 128.9, 128.6, 126.1 (d, *J*_CF_ = 12.8 Hz), 124.5, 115.5, 95.3, 75.6, 72.7, 67.3, 67.0, 60.2, 56.2, 43.0, 33.2, 26.0, 18.5, 16.6, 16.5. IR (film) *ν*_max_: 3461, 2928, 2855, 1616, 1586, 1252, 1090, 1031 cm^−1^. HRMS (ESI): *m/z* calculated for C_24_H_42_FO_5_Si [M + H]^+^: 457.2786. Found: 457.2782.

### 3.16. Synthesis of (2E,4Z)-(5S,6S,E)-5-(2-Fluorophenyl)-8,16,16,17,17-pentamethyl-2,4,11,15- tetraoxa-16-silaoctadec-8-en-6-yl 7-((tert-butyldimethylsilyl)oxy)-8-(diethoxyphosphoryl)- 5-methylocta-2,4-dienoate [(17S,18S) ester ***24***]

(17*S*,18*S*) Ester **24** was obtained as a pale-yellow oil in 91% yield from (17*S*,18*S*) Fragment C9–C18 (**8**) and Fragment C1–C8 (**9**), according to the esterification procedure employed for the preparation of ester **23**. ^1^H NMR (300 MHz, CDCl_3_) *δ* 7.50 (dd, *J* = 14.4, 12.0 Hz, 1H, H-3), 7.42 (t, *J* = 7.2 Hz, 1H, aromatic H), 7.34–7.21 (m, 1H, aromatic H), 7.10 (t, *J* = 7.5 Hz, 1H, aromatic H), 7.01 (t, *J* = 9.3 Hz, 1H, aromatic H), 6.03 (d, *J* = 11.7 Hz, 1H, H-4), 5.76 (d, *J* = 15.0 Hz, 1H, H-2), 5.45–5.37 (m, 1H, H-17), 5.33 (t, *J* = 6.0 Hz, 1H, H-14), 5.08 (d, *J* = 5.1 Hz, 1H, H-18), 4.58 (d, *J* = 6.9 Hz, 1H, O*CH_2_*OCH_3_), 4.51 (d, *J* = 6.6 Hz, 1H, O*CH_2_*OCH_3_), 4.23–4.04 (m, 5H, 2 × O*CH_2_*CH_3_, H-7), 3.93–3.80 (m, 2H, H_2_-13), 3.65 (t, *J* = 6.6 Hz, 2H, H_2_-11), 3.41 (t, *J* = 6.3 Hz, H_2_-9), 3.32 (s, 3H, OCH_2_O*CH_3_*), 2.58–2.54 (m, 2H, H_2_-16), 2.33–2.28 (m, 2H, H_2_-8), 2.03–1.93 (m, 2H, H_2_-6), 1.87 (s, 3H, 5-*CH_3_*), 1.77–1.70 (m, 2H. H_2_-10), 1.64 (s, 3H, 15-CH_3_), 1.31 (t, *J* = 6.9 Hz, 6H, 2 × OCH_2_*CH_3_*), 0.90 (0.88) (s, 3H, TBS), 0.86 (s, 6H, TBS), 0.81 (0.80) (s, 9H, TBS), 0.08 (0.04) (s, 6H, TBS), 0.02 (-0.04) (s, 6H, TBS). ^13^C NMR (75 MHz, CDCl_3_) *δ* 166.7, 160.8 (d, *J*_CF_ = 245.3 Hz), 146.5 (146.4), 146.3 (146.2), 135.7 (135.2), 129.8, 129.0, 126.8, 125.8 (125.6),125.1 (124.7), 124.3 119.2, 115.5 (d, *J*_CF_ = 27.5 Hz), 95.0, 72.9, 72.3, 68.9, 66.8, 61.8, 61.7, 60.1, 56.1, 41.7 (41.2), 36.0 (34.2), 33.1, 32.3, 29.8, 27.0, 26.0, 25.9, 25.8, 24.9, 18.5, 18.1, 18.0, 16.6, 16.5. IR (film) *ν*_max_: 2928, 2856, 1714, 1636, 1250, 1020 cm^−1^. HRMS (ESI): *m/z* calculated for C_43_H_77_FO_10_PSi_2_ [M + H]^+^: 859.4777. Found: 859.4774.

### 3.17. Synthesis of (2E,4Z)-(1S,2S,E)-1-(2-Fluorophenyl)-6-(3-hydroxypropoxy)-1-(methoxymethoxy)- 4-methylhex-4-en-2-yl 8-(diethoxyphosphoryl)-7-hydroxy-5-methylocta-2,4-dienoate [(17S,18S) diol ***26***]

(17*S*,18*S*) Diol **26** was obtained as a colorless syrup in 65% yield according to the TBS deprotection procedure used for the conversion of (17*R*,18*R*) ester **23** to (17*R*,18*R*) diol **25**. ^1^H NMR (300 MHz, CD_3_COCD_3_) *δ* 7.63–7.55 (m, 1H, H-3), 7.52 (t, *J* = 7.8 Hz, 1H, aromatic H), 7.41–7.33 (m, 1H, aromatic H), 7.23 (t, *J* = 7.2 Hz, 1H, aromatic H), 7.14 (t, *J* = 9.0 Hz, 1H, aromatic H), 6.16 (d, *J* = 11.4 Hz, 1H, H-4), 5.80 (d, *J* = 15.0 Hz, 1H, H-2), 5.46 (quin, *J* = 5.1 Hz, 1H, H-17), 5.31 (t, *J* = 6.6 Hz, 1H, H-14), 5.10 (d, *J* = 5.7 Hz, 1H, H-18), 4.61 (d, *J* = 6.6 Hz, 1H, -O*CH_2_*OCH_3_), 4.51 (d, *J* = 6.6 Hz, 1H, -O*CH_2_*OCH_3_), 4.22–4.04 (overlapped, 5H, 2 × –O*CH_2_*CH_3_, H-7), 3.88 (d, *J* = 6.0 Hz, 2H, H_2_-13), 3.57 (t, *J* = 4.8 Hz, 2H, H_2_-11), 3.40 (t, *J* = 6.3 Hz, 2H, H_2_-9), 3.30 (s, 3H, -OCH_2_O*CH_3_*), 2.92 (br.s, 2H, 2 × OH), 2.60–2.57(overlapped, 2H, H_2_-16), 2.29 (d, *J* = 9.3 Hz, 2H, H_2_-8), 2.07–2.04 (m, 2H, H_2_-6), 1.95 (s, 3H, 5-CH_3_), 1.68 (quin, *J* = 6.0 Hz, H_2_-10), 1.65 (s, 3H, 15-CH_3_), 1.29 (t, *J* = 7.2 Hz, 6H, 2 × –OCH_2_*CH_3_*). ^13^C NMR (75 MHz, CDCl_3_) *δ* 166.0, 160.7 (d, *J*_CF_ = 243.8 Hz), 147.0 (d, *J*_CF_ = 9.8 Hz), 140.8, 134.2, 129.8, 129.3, 125.8 (d, *J*_CF_ = 9.8 Hz), 125.6, 125.4 (d, *J*_CF_ = 4.5 Hz), 124.3, 119.1, 115.3 (115.0), 94.7, 72.3, 66.8, 66.7, 65.5, 61.4(61.3), 61.2 (61.1), 59.1, 55.1, 41.2, 40.7, 34.6, 33.02(32.98), 32.0, 24.4, 15.91(15.86), 15.7(15.6). IR (film) *ν*_max_: 3381, 2927, 1709, 1633, 1488, 1019 cm^−1^. HRMS (ESI): *m/z* calculated for C_31_H_49_FO_10_P [M + H]^+^: 631.3048. Found: 631.3045.

### 3.18. Synthesis of (2E,4Z)-(1S,2S,E)-1-(2-Fluorophenyl)-1-(methoxymethoxy)-4-methyl-6- (3-oxopropoxy)hex-4-en-2-yl 8-(diethoxyphosphoryl)-5-methyl-7-oxoocta-2,4-dienoate [ketoaldehyde (17S,18S) ***28***]

The crude product was obtained from (17*S*,18S) diol **26** employing the oxidation procedure used for the synthesis of its antipode **27**. After being confirmed by its ^1^H NMR data, the crude product was directly used for the next step reaction without further purification. ^1^H NMR (300 MHz, CDCl_3_) *δ* 9.72 (t, *J* = 1.8 Hz, 1H, CHO), 7.41 (dt, *J* = 7.5, 1.8 Hz, 1H, aromatic H), 7.31 (dd, *J* = 15.0, 11.7 Hz, 1H, H-3), 7.29–7.22 (m, 1H), 7.11 (dt, *J* = 7.5, 0.9 Hz, 1H), 7.51 (ddd, *J* = 10.2, 8.1, 0.9 Hz, 1H), 6.17 (d, *J* = 11.1 Hz, 1H, H-4), 5.83 (d, *J* = 15.0 Hz, 1H, H-2), 5.45–5.38 (m, 1H), 5.31 (t, *J* = 5.7 Hz, 1H, H-14), 5.07 (d, *J* = 5.4 Hz, 1H), 4.57 (d, *J* = 6.9 Hz, 1H, O*CH_2_*OCH_3_), 4.50 (d, *J* = 6.9 Hz, 1 H, O*CH_2_*OCH_3_), 4.20–4.11 (m, 5H, 2 × O*CH_2_*CH_3_, H-7), 3.90 (t, *J* = 7.2 Hz, 2H, H_2_-13), 3.65 (t, *J* = 6.0 Hz, 2H, H_2_-11), 3.31 (s, 3H, OCH_3_), 3.10 (d, *J* = 22.8 Hz, 2H, H_2_-8), 2.39–2.19 (m, 2H, H_2_-10), 1.87 (s, 3H, 5-CH_3_), 1.63 (s, 3H, 15-CH_3_).

### 3.19. Synthesis of (S,3E,9E,11Z,15E)-6-((S)-(2-Fluorophenyl)(methoxymethoxy)methyl)-4,12- dimethyl-1,7-dioxacyclooctadeca-3,9,11,15-tetraene-8,14-dione [(17S,18S) macrolide ***30***]

(17*S*,18*S*) Macrolide **30** was synthesized according to the HWE ring closing procedure employed for the conversion of **27** to **29**. Colorless syrup, 68% yield for two steps. [α]: +8.1 (*c* = 0.22, MeOH). ^1^H NMR (300 MHz, CDCl_3_) *δ* 7.59 (dd, *J* = 15.0, 11.4 Hz, 1H, H-3), 7.46 (dt, *J* = 7.5, 1.8 Hz, 1H, aromatic H), 7.36–7.25 (m, 1H, aromatic H), 7.15 (dt, *J* = 7.5, 1.2 Hz, 1H, aromatic H), 7.04 (ddd, *J* = 9.9, 8.1, 1.2 Hz, 1H, aromatic H), 6.81 (dt, *J* = 15.9, 6.9 Hz, 1H, H-9), 6.11 (d, *J* = 11.4 Hz, 1H, H-4), 6.00 (d, *J* = 16.2 Hz, 1H, H-8), 5.90 (d, *J* = 15.0 Hz, 1H, H-2), 5.50 (dd, *J* = 11.4, 6.3 Hz, 1H, H-17), 5.26 (dd, *J* = 7.5, 4.5 Hz, 1H, H-14), 5.11 (d, *J* = 6.3 Hz, 1H, H-18), 4.59 (d, *J* = 6.9 Hz, 1H,–O*CH_2_*OCH_3_), 4.52 (d, *J* = 6.6 Hz, 1H, –O*CH_2_*OCH_3_), 3.98 (dd, *J* = 12.0, 8.1 Hz, 1H, H-16), 3.88 – 3.81 (overlapped, 2H, H_2_-13), 3.46 – 3.31 (overlapped, 2H, H_2_-11), 3.33 (s, 3H, O*CH_3_*), 3.14 (d, *J* = 12.6 Hz, 1H, H-16), 2.42–2.35 (m, 2H, H_2_-10), 2.33 (d, *J* = 11.7 Hz, 1H, H-6), 2.04 (d, *J* = 11.7 Hz, 1H, H-6), 1.82 (s, 3H, 5-*CH_3_*), 1.63 (s, 3H, 15-*CH_3_*). ^13^C NMR (75 MHz, CDCl_3_) *δ* 197.1, 166.6, 160.9 (d, *J*_CF_ = 244.5 Hz), 146.8, 142.5, 139.8, 134.3, 130.2, 129.9, 129.0, 125.9 (d, *J*_CF_ = 7.5 Hz), 125.5 (d, *J* = 13.5 Hz), 125.2 (d, *J*_CF_ = 4.5 Hz), 124.5, 120.9 (d, *J*_CF_ = 3.8 Hz), 115.6 (d, *J*_CF_ = 21.0 Hz), 94.9, 72.9, 72.6, 67.9, 67.8, 56.0, 45.9, 41.4, 33.1, 24.1, 16.7. IR (film) *ν*_max_: 2925, 2854, 1714, 1669, 1634, 1489, 1359, 1280 cm^−1^. HRMS (ESI): *m/z* calculated for C_27_H_34_FO_6_ [M + H]^+^: 473.2339. Found: 473.2332.

### 3.20. Synthesis of (S,3E,9E,11Z,15E)-6-((S)-(2-Fluorophenyl)(hydroxy)methyl)-4,12-dimethyl- 1,7-dioxacyclooctadeca-3,9,11,15-tetraene-8,14-dione [(17S,18S) zampanolide mimic ***6***]

(17*S*,18*S*) Zampanolide mimic **6** was obtained according to the MOM deprotection procedure used for the conversion of (17*R*,18*R*) macrolide **29** to (17S,18S) zampanolide mimic **5**. Colorless syrup, 45% yield. [α]: +15.6 (*c* = 0.15, MeOH). ^1^H NMR (300 MHz, CD_3_COCD_3_) *δ* 7.61 (dd, *J* = 15.0, 11.1 Hz, 1H, H-3), 7.34 (dd, *J* = 14.4, 7.2 Hz, 1H, aromatic H), 7.21 (t, *J* = 7.5 Hz, 1H, aromatic H), 7.10 (dd, *J* = 10.5, 8.1 Hz, 1H, aromatic H), 6.83 (dt, *J* = 16.2, 6.3 Hz, 1H, H-9), 6.20 (d, *J* = 11.1 Hz, 1H, H-4), 6.06 (d, *J* = 15.9 Hz, H-8), 5.88 (d, *J* = 12.6 Hz, 1H, H-2), 5.44 (dd, *J* = 11.1, 5.4 Hz, 1H, H-17), 5.27 (t, *J* = 5.4 Hz, 1H, H-14), 5.17 (t, *J* = 5.4 Hz, 1H, H-18), 4.81 (d, *J* = 5.4 Hz, 1H, OH), 4.07–3.97 (m, 1H, H-16), 3.90 (d, *J* = 12.9 Hz, 1H, H-13), 3.83 (dd, *J* = 12.3, 4.5 Hz, 1H, H-13), 3.48– 3.32 (m, 2H, H_2_-11), 3.17 (d, *J* = 12.6 Hz, 1H, H-16), 2.42–2.33 (overlapped, 3H, H_2_-10, H-6), 2.19 (d, *J* = 13.8 Hz, 1H, H-6), 1.81 (s, 3H, 5-*CH_3_*), 1.64 (s, 3H, 15-*CH_3_*). ^13^C NMR (75 MHz, CD_3_COCD_3_) *δ* 196.5, 166.5, 160.5 (d, *J*_CF_ = 242.3 Hz), 146.6, 142.7, 139.8, 134.7, 130.6, 129.7, 129.5, 126.3, 126.2, 125.7, 124.8, 121.7, 115.6, 73.8, 69.0, 68.9, 68.2, 45.8, 41.6, 33.2, 23.8, 16.3. IR (film) *ν*_max_: 3447, 2916, 2855, 1710, 1669, 1633, 1489, 1456, 1359, 1281, 1148 cm^−1^. HRMS (ESI): *m/z* calculated for C_25_H_30_FO_5_ [M + H]^+^: 429.2077. Found: 429.2073.

### 3.21. Cell Culture

All cell lines were initially purchased from American Type Culture Collection (ATCC). The PC-3, PC-3/DTX, and LNCaP prostate cancer cell lines were routinely cultured in RPMI-1640 medium supplemented with 10% FBS and 1% penicillin/streptomycin. Cultures were maintained in a high humidity environment supplemented with 5% carbon dioxide at a temperature of 37 °C. The DU145 and DU145/DTX prostate cancer cells were routinely cultured in Eagle’s Minimum Essential Medium (EMEM) supplemented with 10% FBS and 1% penicillin/streptomycin.

The procedure illustrated in the literature [29,30] was adapted to establish docetaxel-resistant prostate cancer cell lines. Specifically, docetaxel-resistant DU145 and PC-3 cell lines (DU145/DTX and PC-3/DTX) were developed over a period of one year by stepwise increased concentrations of docetaxel. Cells were repeatedly conserved in an appropriate concentration of docetaxel, starting with IC_50_ value of the respective parent cell lines. Docetaxel-containing media will be replaced every 2–3 days. The concentration of docetaxel was increased when cells exhibited resistance to treatments.

### 3.22. WST-1 Cell Proliferation Assay

PC-3, PC-3/DTX, DU145, DU145/DTX, or LNCaP cells were placed in 96-well plates at a density of 3200 cells each well in 200 µL of culture medium. The cells were then treated with synthesized mimics, or positive reference separately at different doses for 3 days, while equal treatment volumes of DMSO were used as vehicle control. After the cells were cultured in a CO_2_ incubator at 37 °C for three days, the premixed WST-1 cell proliferation reagent (10 µL, Clontech) was added to each well. The cells were incubated for additional 3 h at 37 °C before mixing gently for one minute on an orbital shaker to ensure homogeneous distribution of color. A microplate-reader (Synergy HT, BioTek) was used to measure the absorbance of each well at a wavelength of 430 nm. The half-maximal inhibitory concentration (IC_50_ value) is the concentration of each compound that inhibits cell proliferation by 50% under the experimental conditions. Each of IC_50_ value is represented as the average from triplicate determinations that were reproducible and statistically significant. The IC_50_ values were calculated based on dose–response curves from at least five dosages for each compound.

### 3.23. Statistical Analysis

All data are represented as the mean ± standard deviation (S.D.) for the number of experiments indicated. Other differences between treated and control groups were analyzed using the Student’s t-test. A *p*-value < 0.05 was considered statistically significant.

## 4. Conclusions

In summary, two new zampanolide mimics have been designed and each of them has been achieved through a 25-step chemical transformation starting from commercially available 2-butyn-1-ol. Yamaguchi esterification and intramolecular Horner–Wadsworth–Emmons condensation were employed as crucial reactions to build up the C-17 and C-1 ester moiety and to close the lactone ring. The chiral centers at C17 and C18 in the Fragment C9–C18 (**7** and **8**) were introduced by the Sharpless asymmetric dihydroxylation using the commercially available AD-mix formulations. Our WST-1 cell proliferation assay data in both docetaxel-resistant and docetaxel-sensitive prostate cancer cell lines revealed that compound **6** is the optimal mimic and the newly designed side chain can act as a bioisostere for the chemically fragile *N*-acetyl hemiminal side chain in zampanolide.

## Figures and Tables

**Table 1:** Antiproliferative potency of zampanolide mimics against docetaxel-sensitive and docetaxel-resistant prostate cancer cells.

Compounds	IC_50_: (µM) *^a^*		R/S *^a^*	IC_50_: (µM) *^a^*		R/S *^a^*	IC_50_: (µM)
	PC-3	PC-3/DTX		DU145	DU145/DTX		LNCaP
Docetaxel	0.0019 ± 0.0006	2.34 ± 0.25	1232	0.0012 ± 0.0003	8.58 ± 0.39	7150	0.0002 ± 0.00005
**3** *^b^*	0.88 ± 0.19	2.97 ± 0.09	1.3	0.86 ± 0.25	2.78 ± 0.16	3.2	0.76 ± 0.29
**4** *^b^*	0.35 ± 0.05	0.30 ± 0.05	0.9	0.29 ± 0.03	0.46 ± 0.10	1.6	0.35 ± 0.02
**29**	9.36 ± 0.15	3.97 ± 0.24	0.42	3.98 ± 0.57	4.42 ± 0.55	1.1	5.29 ± 0.49
**5**	8.72 ± 2.21	3.63 ± 0.11	0.42	3.87 ± 0.23	5.03 ± 1.11	1.3	6.51 ± 0.60
**30**	5.80 ± 0.21	3.71 ± 0.72	0.64	2.28 ± 0.51	4.30 ± 0.79	1.9	3.15 ± 0.19
**6**	3.91 ± 0.73	2.31 ± 0.32	0.59	2.27 ± 0.39	1.00 ± 0.91	0.44	1.92 ± 0.29

*^a^* The relative resistance of the two cell lines obtained by dividing the IC_50_ value of the resistance cell line by that of the parental cell line. *^b^* These data were reported in our previous paper [21].

## Supplementary Materials

The following are available online at https://www.mdpi.com/1420-3049/25/2/362/s1, NMR spectra (^1^H and ^13^C) of the zampanolide mimics **5** and **6**, as well as the macrolides **30** and **31,** and intermediates **7–8, 14–21,** and **23–26**.
