# Supplementary material for: New Zampanolide Mimics: Design, Synthesis, and Antiproliferative Evaluation
Source: Molecules. 2020 Jan 15;25(2):362. doi: 10.3390/molecules25020362 (PMC7024368; doi:10.3390/molecules25020362)
Supplement: Supplementary file 1 [file molecules-25-00362-s001.pdf]

Supplementary Data

New Zampanolide Mimics: Design, Synthesis, and Antiproliferative Evaluation

Guanglin Chen[1], Ziran Jiang[1], Qiang Zhang[2], Guangdi Wang[2], and Qiao-Hong Chen[1,*]

[1]Department of Chemistry, California State University, Fresno, 2555 E. San Ramon Avenue, M/S SB70, Fresno, CA 93740, USA
[2]Department of Chemistry and RCMI Cancer Research Center, Xavier University of Louisiana, 1 Drexel Drive, New Orleans, LA 70125, USA

List of contents:

^{1}H NMR spectrum of **14** in $CDCl_3$……S3
^{13}C NMR spectrum of **14** in $CDCl_3$……S4
^{1}H NMR spectrum of **15** in $CDCl_3$……S5
^{13}C NMR spectrum of **15** in $CDCl_3$……S6
^{1}H NMR spectrum of **16** in $CDCl_3$……S7
^{13}C NMR spectrum of **16** in $CDCl_3$……S8
^{1}H NMR spectrum of **17** in $CDCl_3$……S9
^{13}C NMR spectrum of **17** in $CDCl_3$……S10
^{1}H NMR spectrum of **18** in $CDCl_3$……S11
^{13}C NMR spectrum of **18** in $CDCl_3$……S12
^{1}H NMR spectrum of **19** in $CDCl_3$……S13
^{13}C NMR spectrum of **19** in $CDCl_3$……S14
^{1}H NMR spectrum of **20** in $CDCl_3$……S15
^{13}C NMR spectrum of **20** in $CDCl_3$……S16
^{1}H NMR spectrum of **21** in $CDCl_3$……S17
^{13}C NMR spectrum of **21** in $CDCl_3$……S18
^{1}H NMR spectrum of **7** in $CDCl_3$……S19
^{13}C NMR spectrum of **7** in $CDCl_3$……S20
^{1}H NMR spectrum of **8** in $CDCl_3$……S21

^{13}C NMR spectrum of **8** in $CDCl_3$…………..……………………………………………...S22
^{1}H NMR spectrum of **23** in $CDCl_3$…………………………………………………………S23
^{13}C NMR spectrum of **23** in $CDCl_3$…………...…………………………………………….S24
^{1}H NMR spectrum of **24** in $CDCl_3$…………………………………………………………S25
^{13}C NMR spectrum of **24** in $CDCl_3$…………...…………………………………………….S26
^{1}H NMR spectrum of **25** in $CDCl_3$…………………………………………………………S27
^{13}C NMR spectrum of **25** in $CDCl_3$…………...…………………………………………….S28
^{1}H NMR spectrum of **26** in CD_3COCD_3…………………………………………………….S29
^{13}C NMR spectrum of **26** in CD_3**COCD$_3$**…………...……………………………………….S30
^{1}H NMR spectrum of **29** in $CDCl_3$…………………………………………………………S31
^{13}C NMR spectrum of **29** in $CDCl_3$…………...…………………………………………….S32
^{1}H NMR spectrum of **30** in $CDCl_3$…………………………………………………………S33
^{13}C NMR spectrum of **30** in $CDCl_3$…………...…………………………………………….S34
^{1}H NMR spectrum of **5** in $CDCl_3$……………………………………………………...S35
^{13}C NMR spectrum of **5** in $CDCl_3$…………...……………………………………………...S36
^{1}H NMR spectrum of **6** in CD_3COCD_3…………………………………………………...S37
^{13}C NMR spectrum of **6** in CD_3**COCD$_3$**…………...…………………………………….S38

Corresponding author.

*E-mail: qchen@csufresno.edu. Phone: (+1)5592782394. Fax: (+1)5592784402.

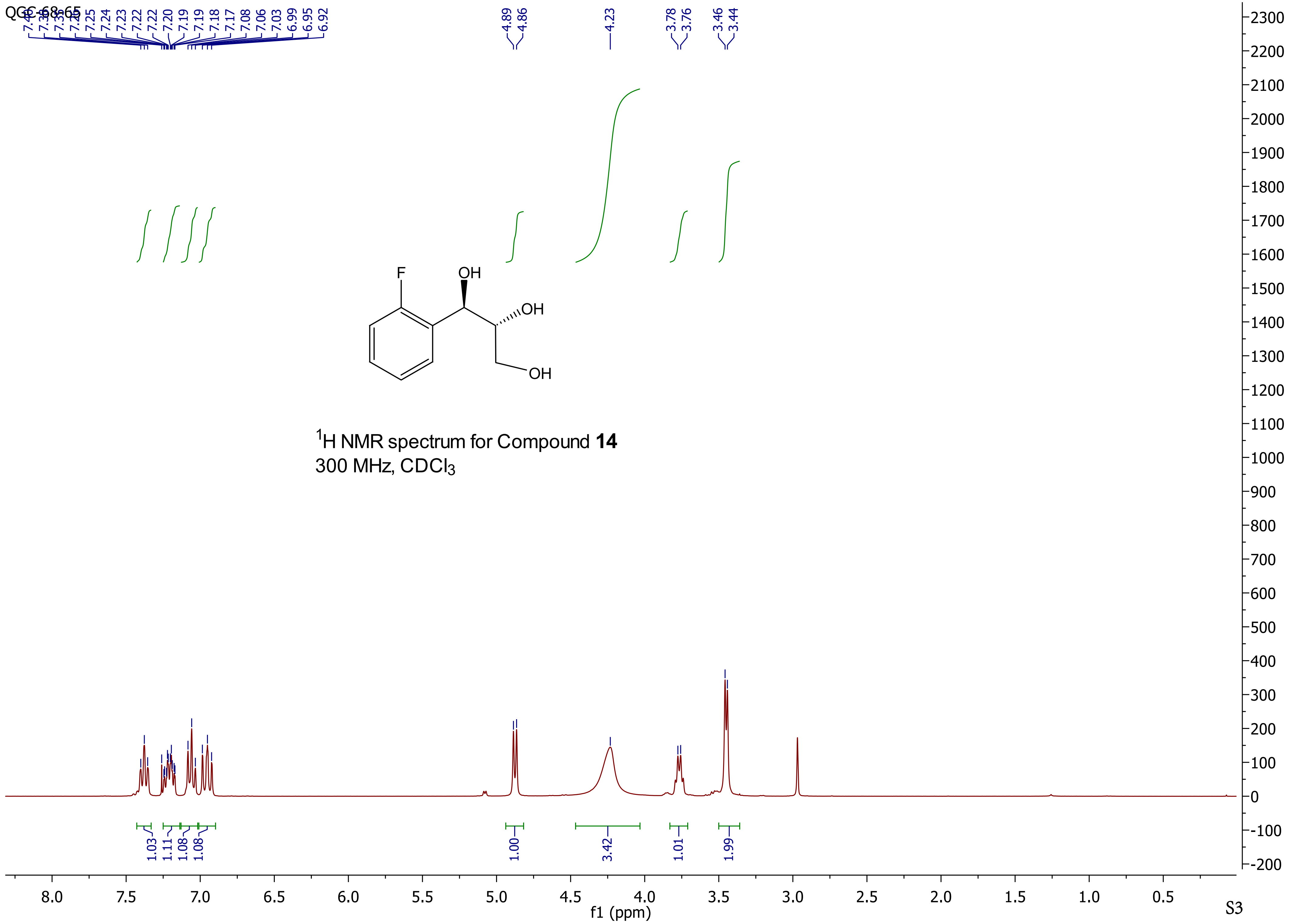

^{1}H NMR spectrum for Compound **14**
300 MHz, $CDCl_3$

QGC-68-65

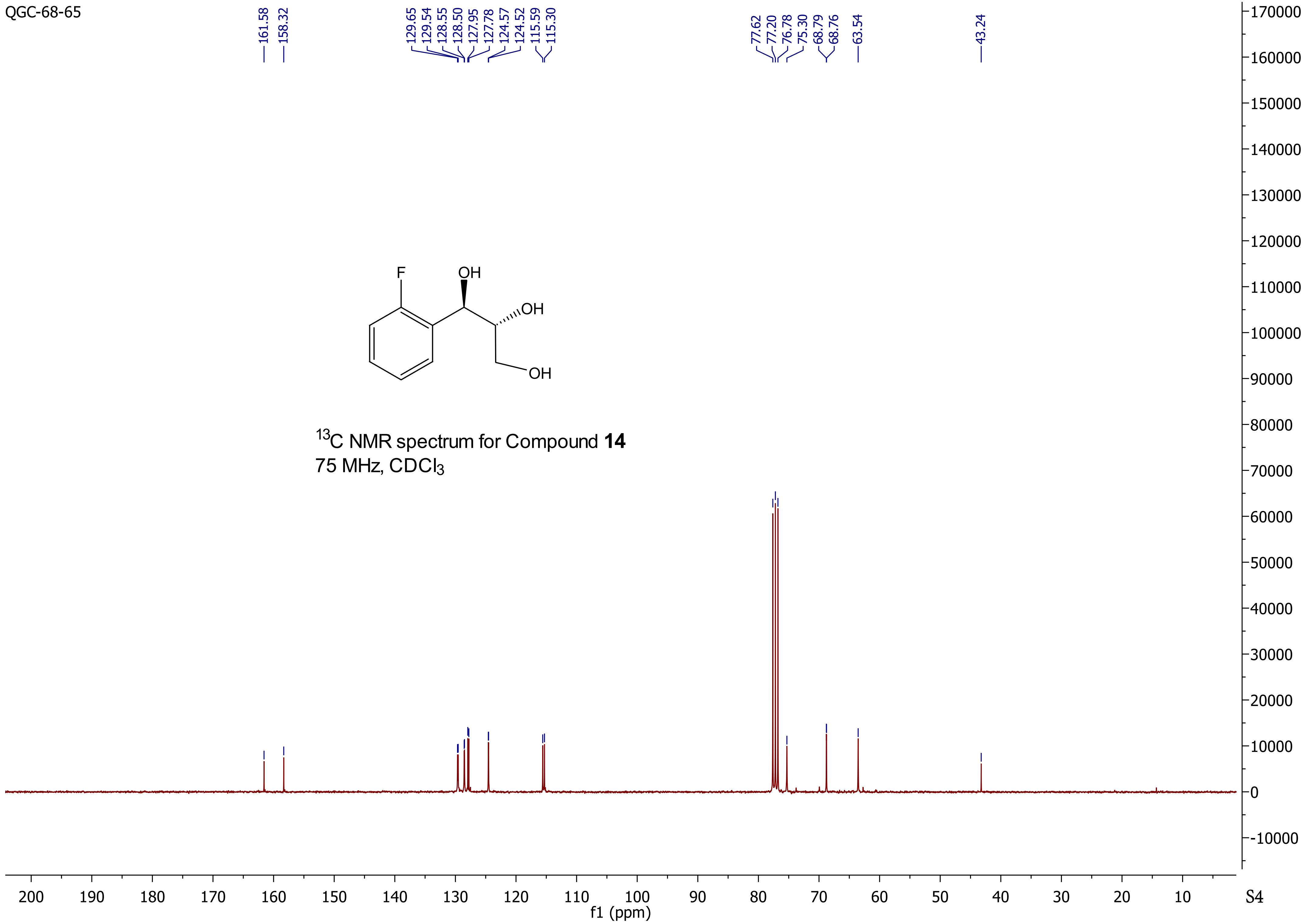

^{13}C NMR spectrum for Compound **14**
75 MHz, $CDCl_3$

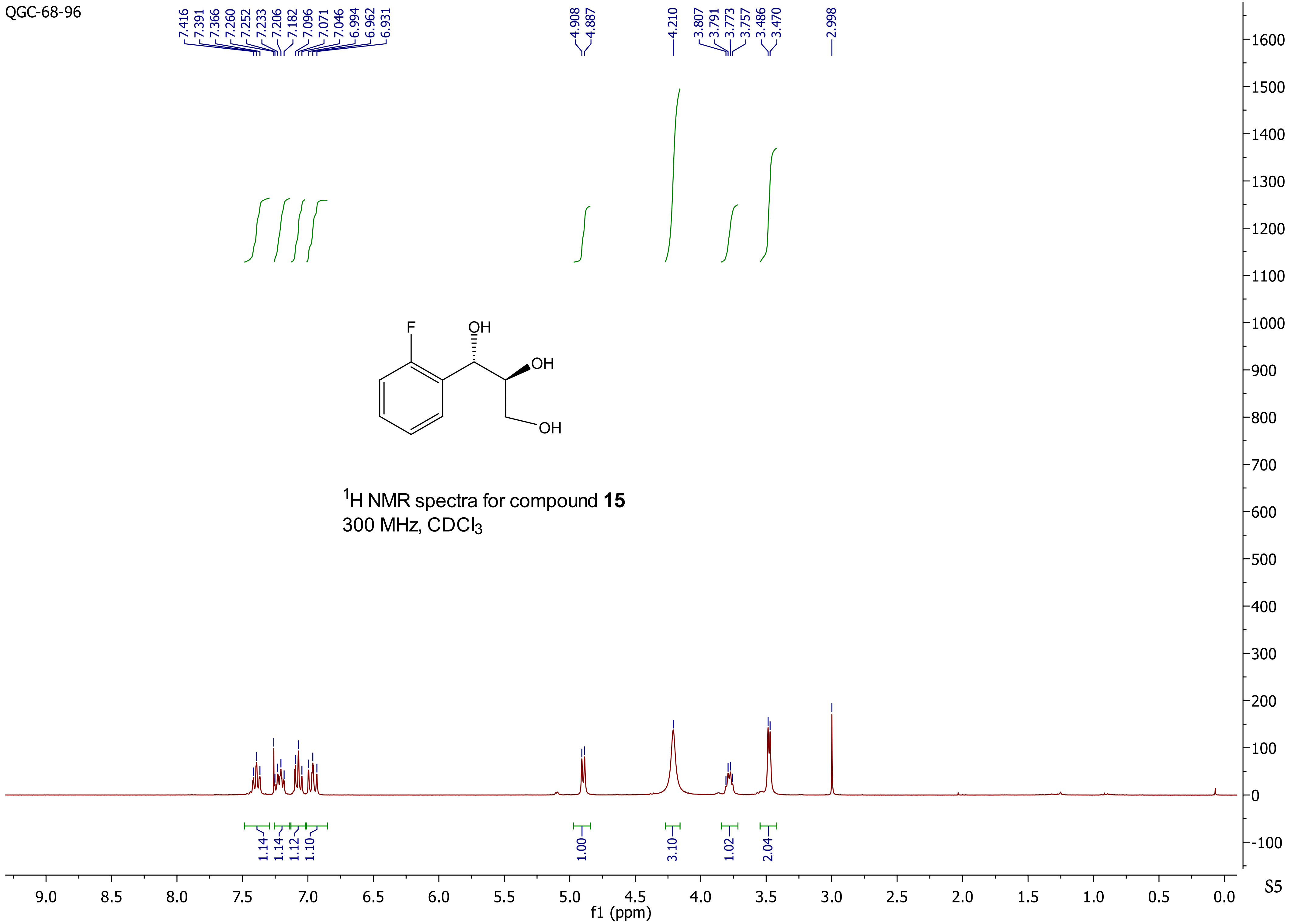

^{1}H NMR spectra for compound **15**
300 MHz, $CDCl_3$

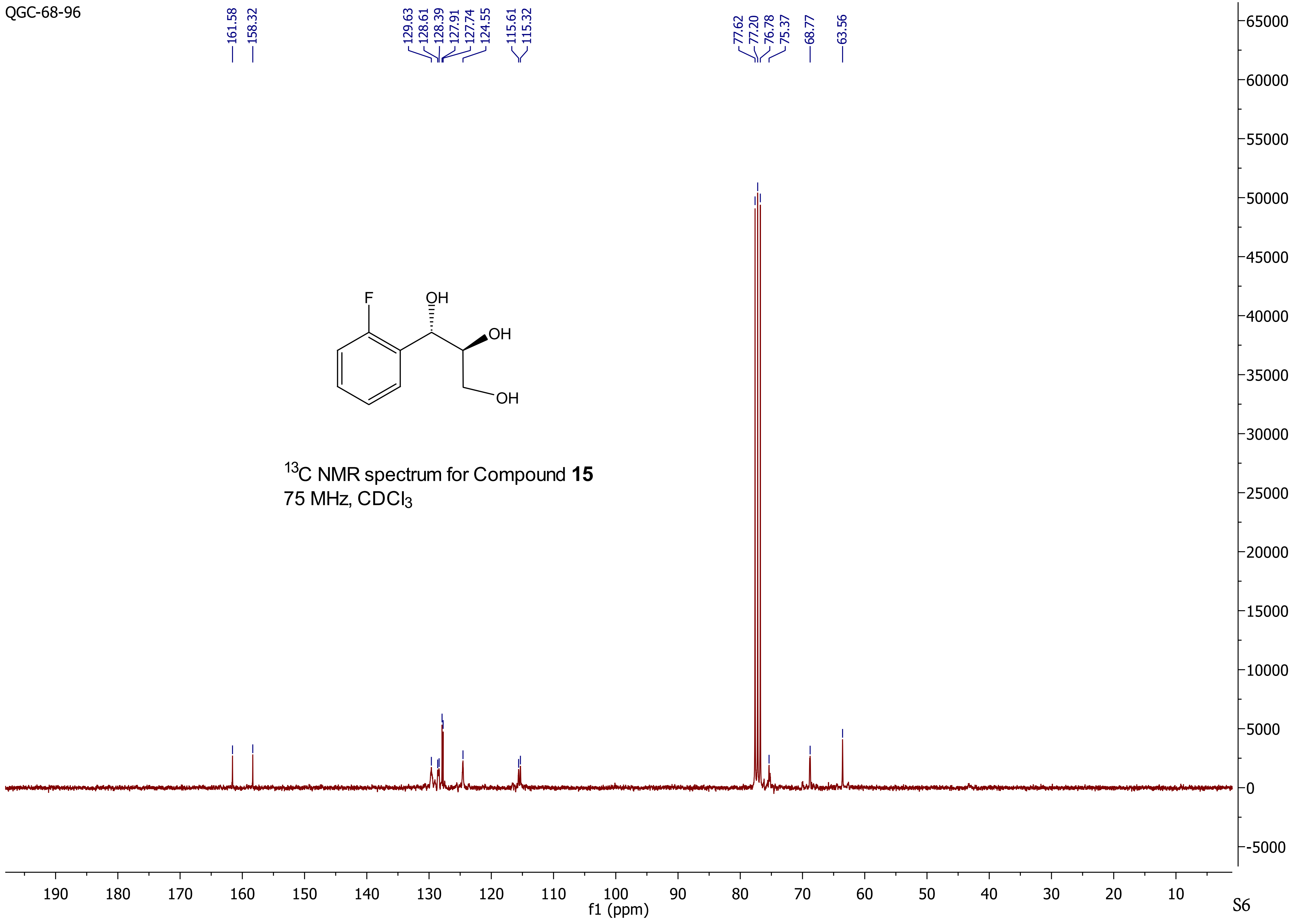
QGC-68-96
161.58
158.32
129.63
128.61
128.39
127.91
127.74
124.55
115.61
115.32
77.62
77.20
76.78
75.37
68.77
63.56
F
OH
OH
OH
^{13}C NMR spectrum for Compound **15**
75 MHz, $CDCl_3$
65000
60000
55000
50000
45000
40000
35000
30000
25000
20000
15000
10000
5000
0
-5000
190
180
170
160
150
140
130
120
110
100
90
80
70
60
50
40
30
20
10
f1 (ppm)

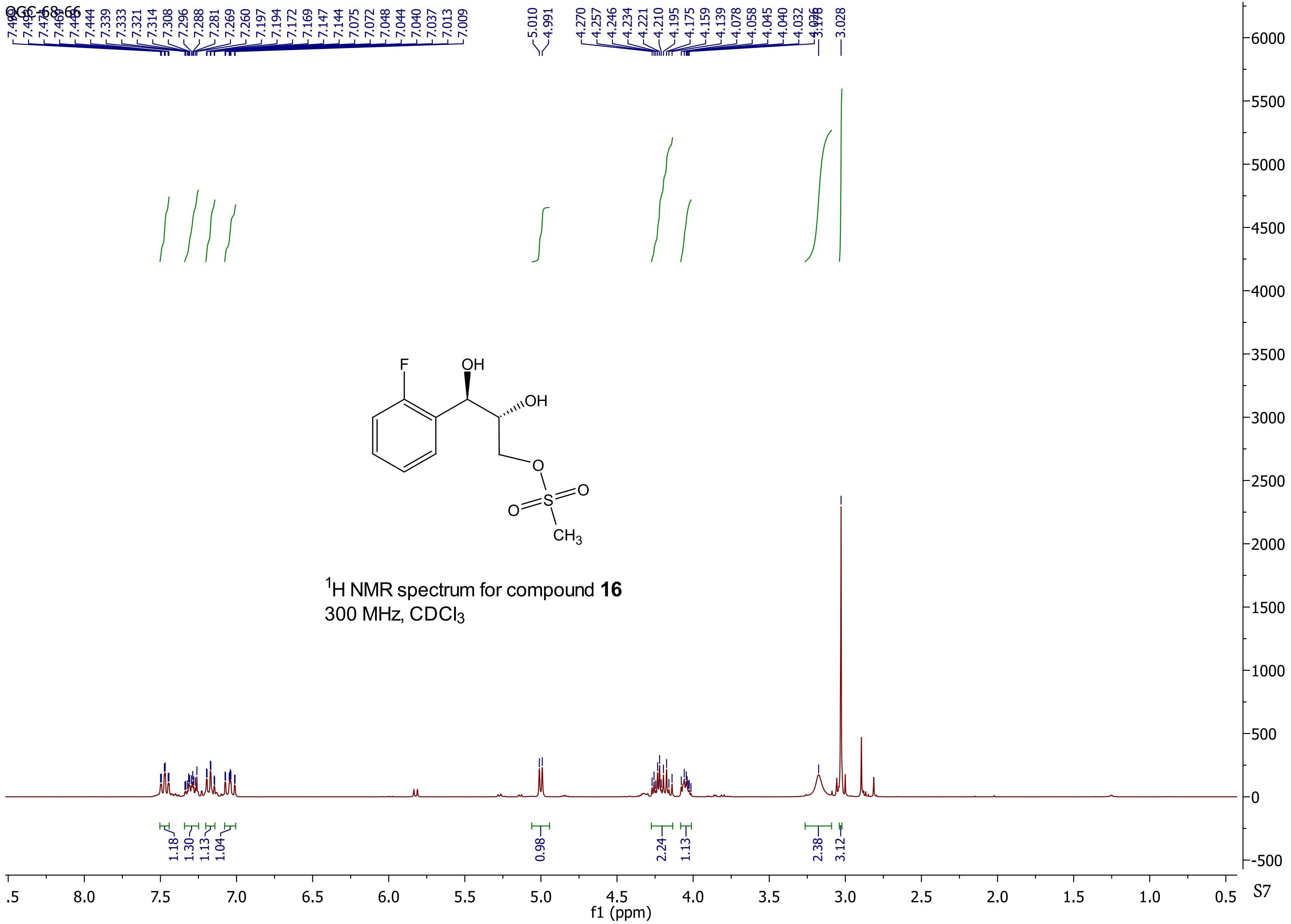

^{1}H NMR spectrum for compound **16**
300 MHz, $CDCl_3$

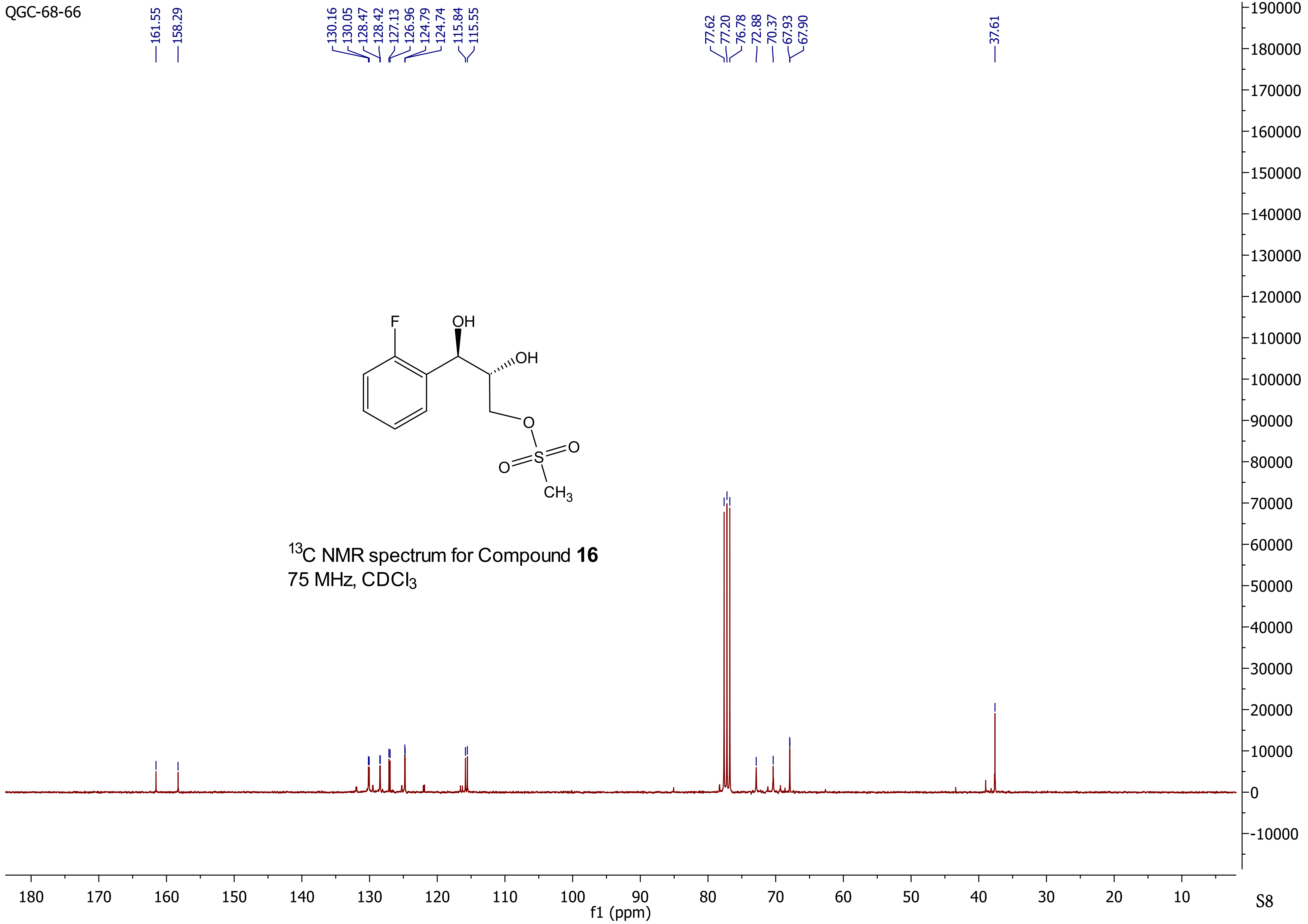

^{13}C NMR spectrum for Compound **16**
75 MHz, $CDCl_3$

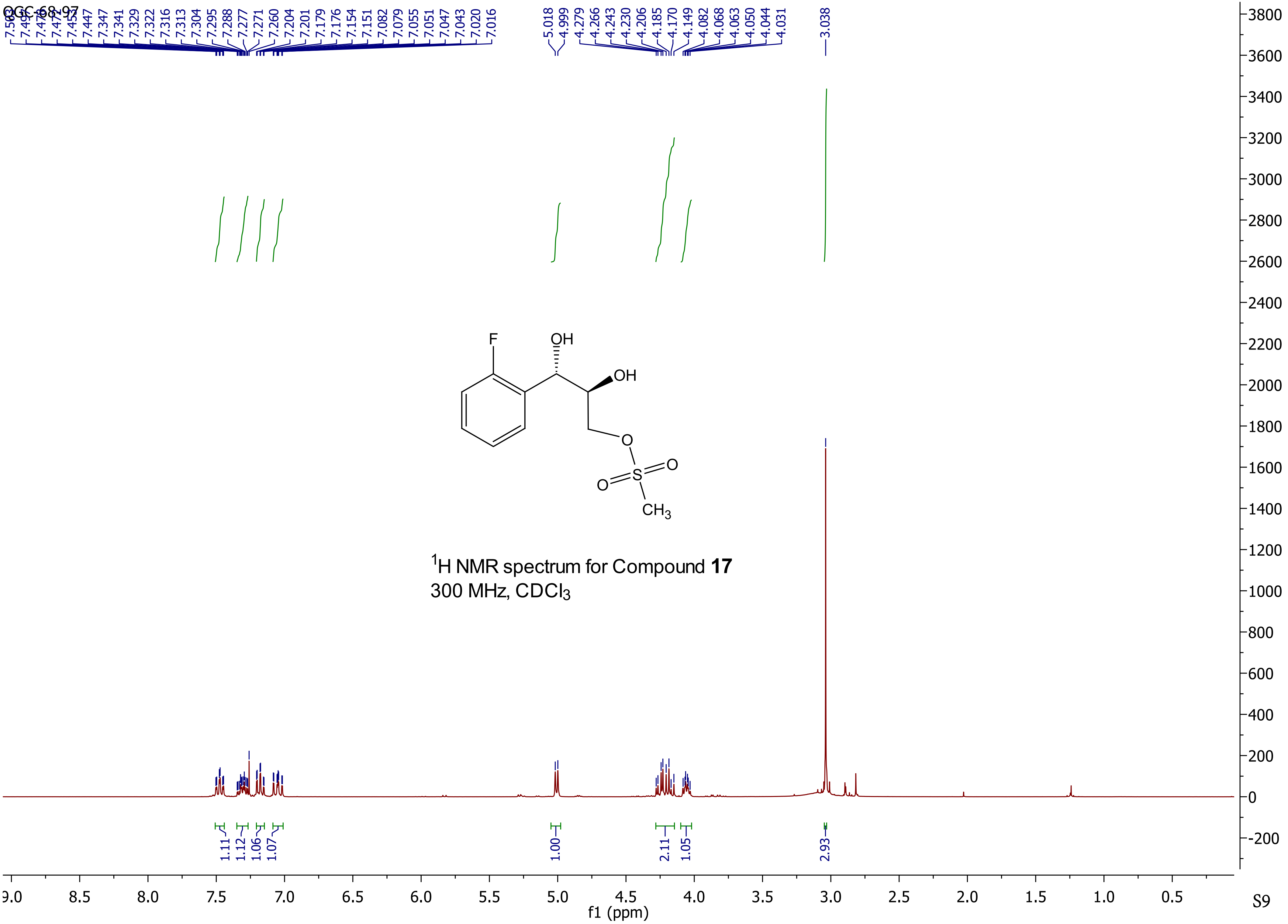
OGC-68-97
7.447
7.347
7.341
7.329
7.322
7.316
7.313
7.304
7.295
7.288
7.277
7.271
7.260
7.204
7.201
7.179
7.176
7.154
7.151
7.082
7.079
7.055
7.051
7.047
7.043
7.020
7.016
5.018
4.999
4.279
4.266
4.243
4.230
4.206
4.185
4.170
4.149
4.082
4.068
4.063
4.050
4.044
4.031
3.038
F
OH
OH
O
O
S
O
CH3
^{1}H NMR spectrum for Compound **17**
300 MHz, $CDCl_3$
1.11
1.12
1.06
1.07
1.00
2.11
1.05
2.93
f1 (ppm)

QGC-68-97

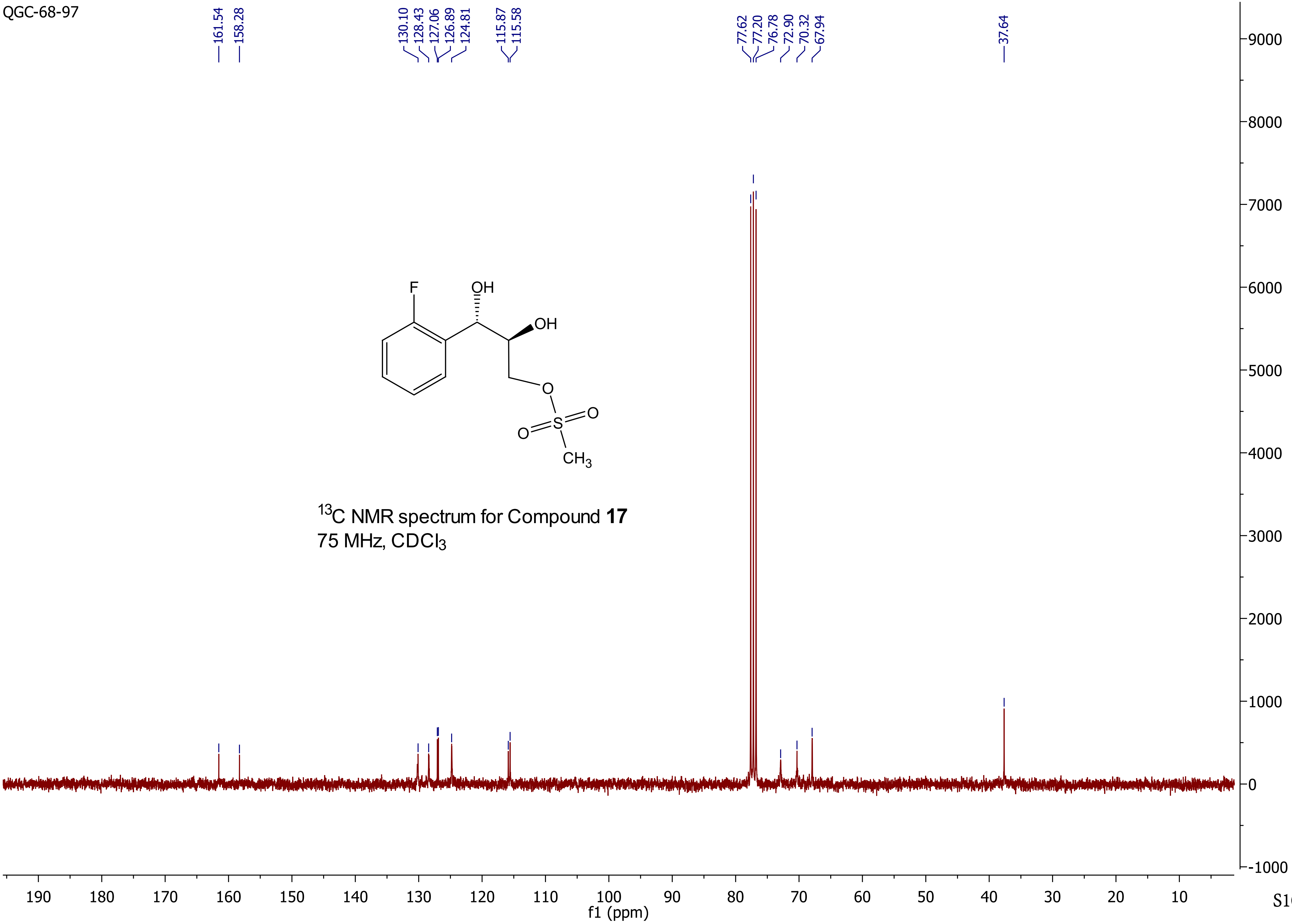

^{13}C NMR spectrum for Compound **17**
75 MHz, $CDCl_3$

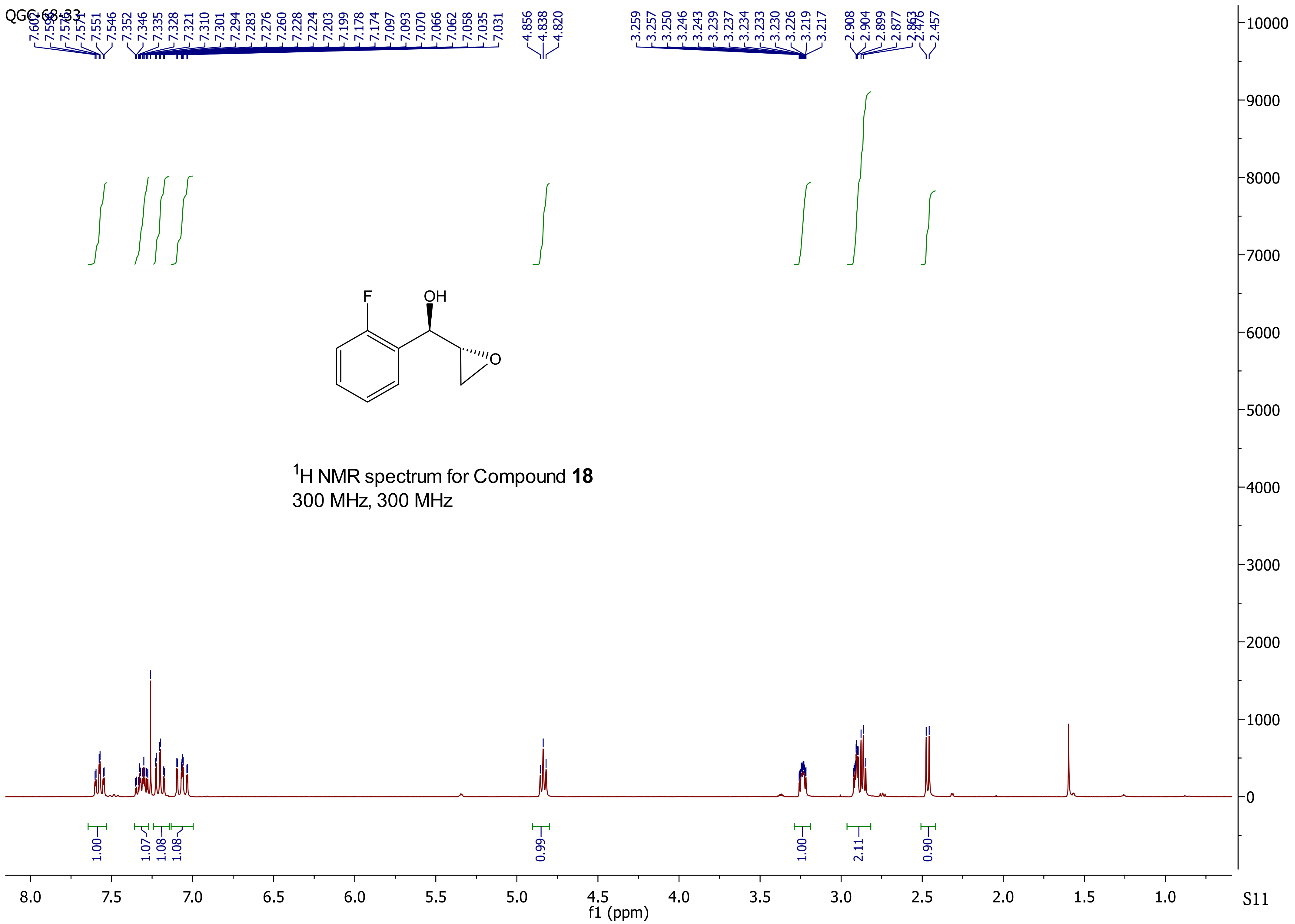
QGC-68-33
F
OH
O
^{1}H NMR spectrum for Compound **18**
300 MHz, 300 MHz
f1 (ppm)

QGC-68-33

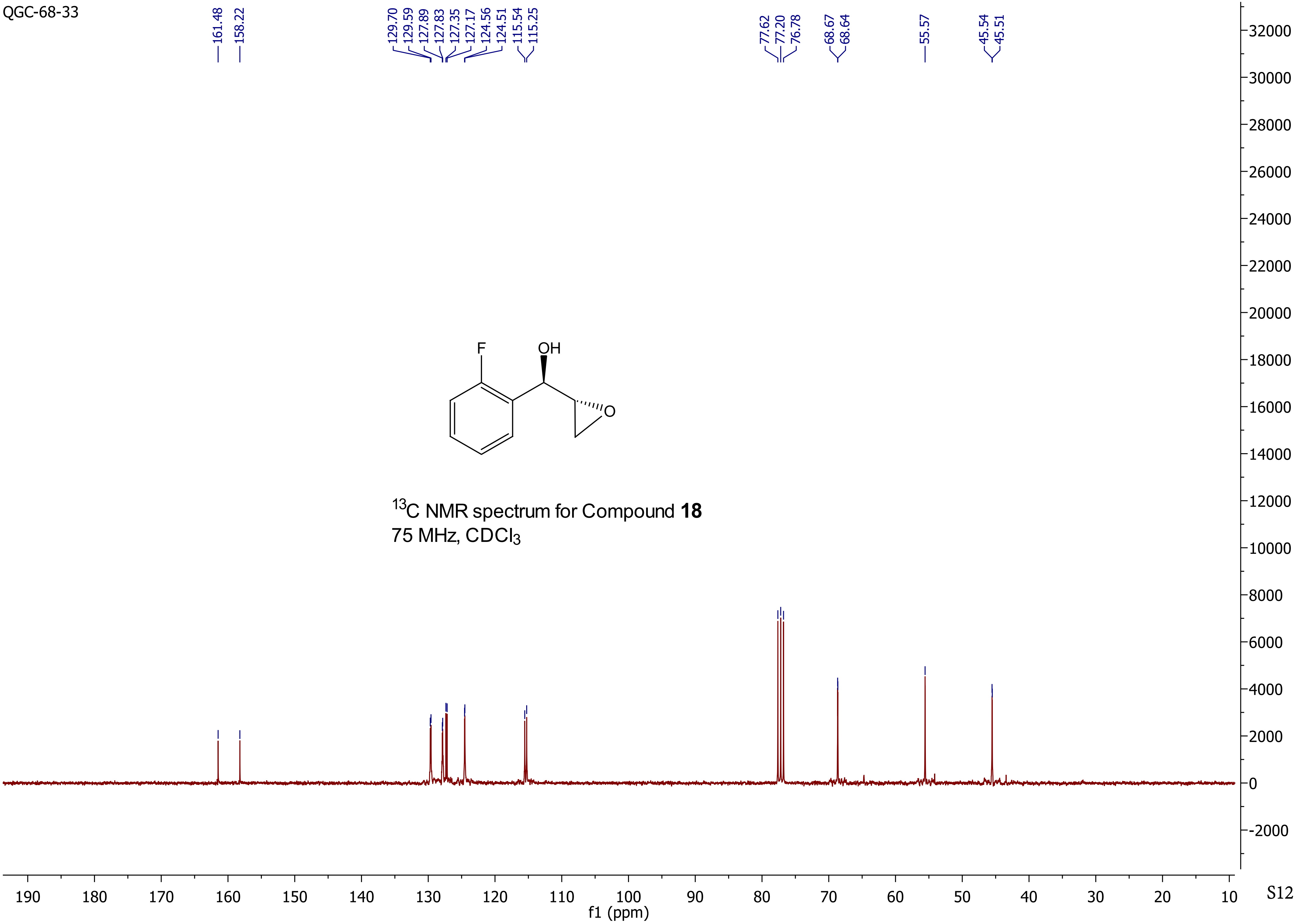

^{13}C NMR spectrum for Compound **18**

75 MHz, $CDCl_3$

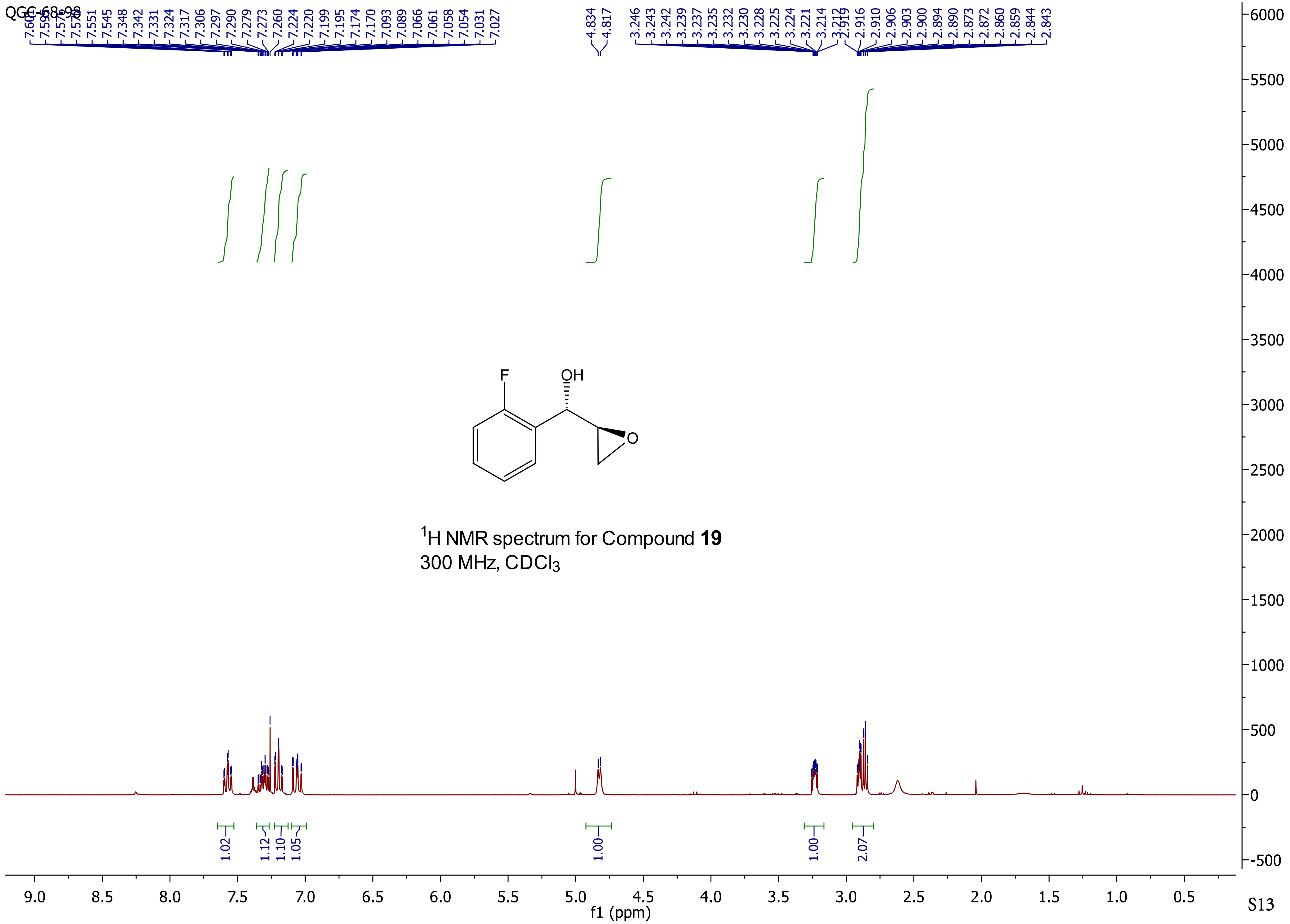
QGC-68-98
7.551
7.545
7.348
7.342
7.331
7.324
7.317
7.306
7.297
7.290
7.279
7.273
7.260
7.224
7.220
7.199
7.195
7.174
7.170
7.093
7.089
7.066
7.061
7.058
7.054
7.031
7.027
4.834
4.817
3.246
3.243
3.242
3.239
3.237
3.235
3.232
3.230
3.228
3.225
3.224
3.221
3.214
2.916
2.910
2.906
2.903
2.900
2.894
2.890
2.873
2.872
2.860
2.859
2.844
2.843
F
OH
O
^{1}H NMR spectrum for Compound **19**
300 MHz, $CDCl_3$
1.02
1.12
1.10
1.05
1.00
1.00
2.07
f1 (ppm)

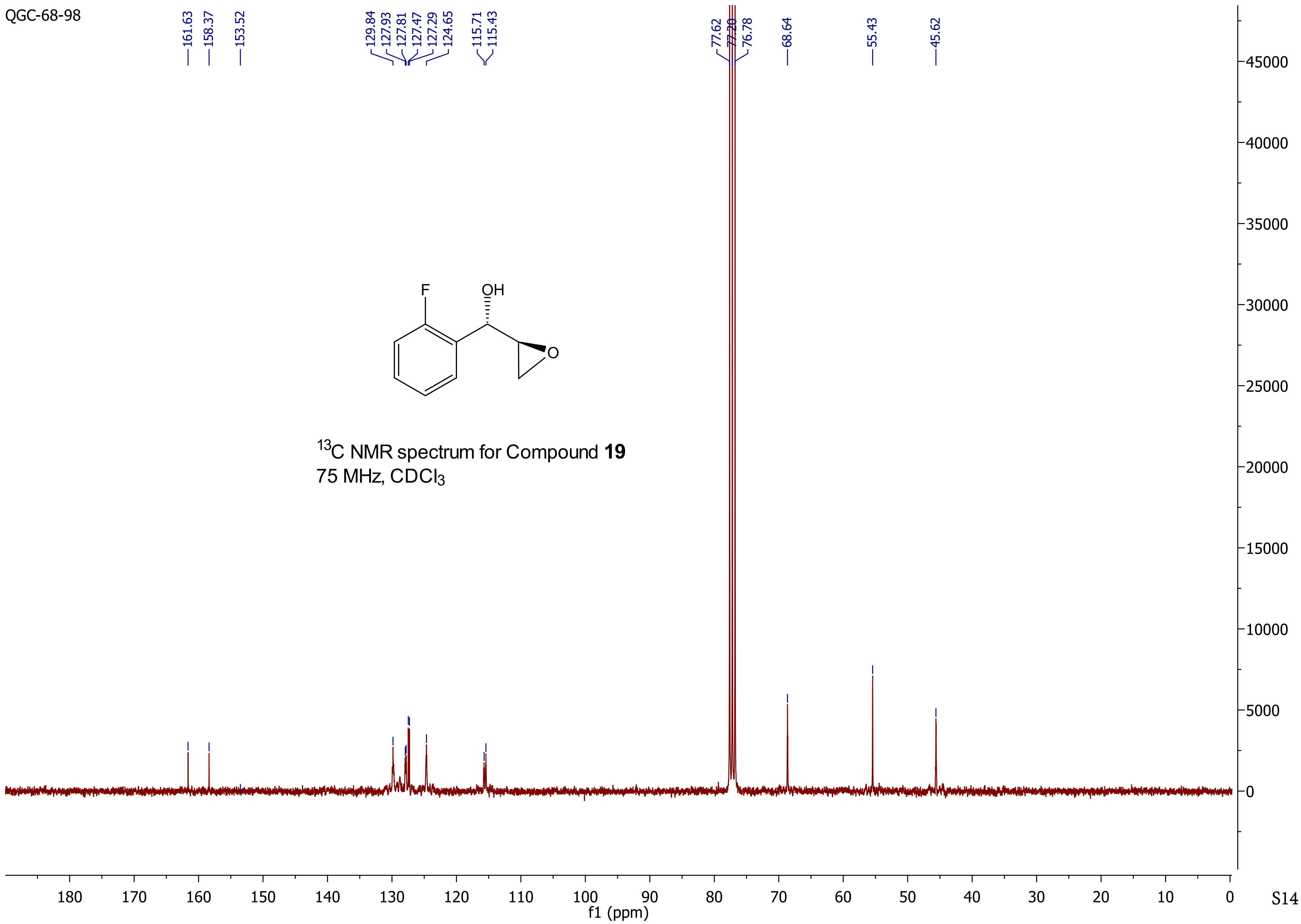
QGC-68-98
161.63
158.37
153.52
129.84
127.93
127.81
127.47
127.29
124.65
115.71
115.43
77.62
77.20
76.78
68.64
55.43
45.62
F
OH
O
^{13}C NMR spectrum for Compound **19**
75 MHz, $CDCl_3$
f1 (ppm)

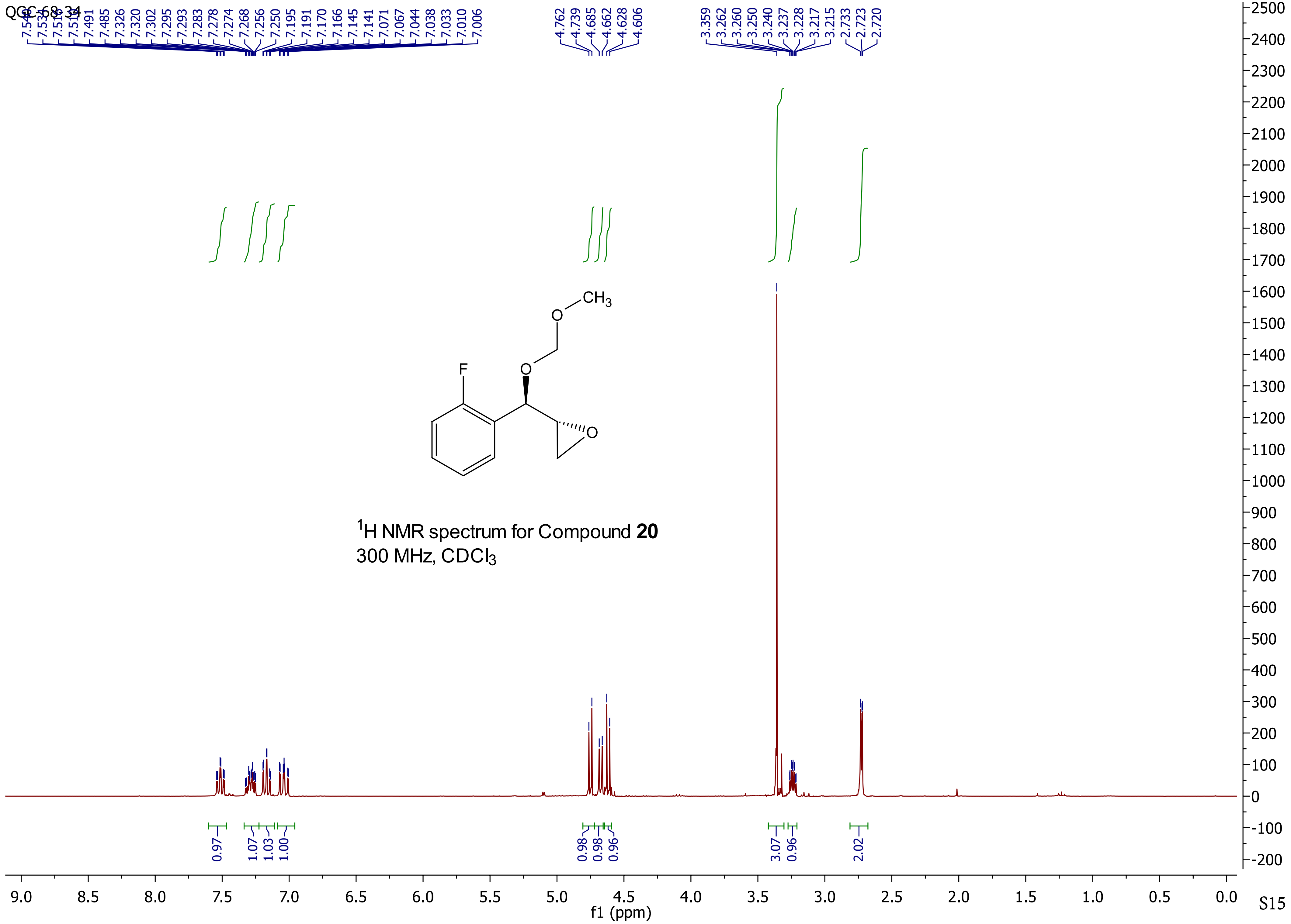
QGC-68-34
CH3
O
O
F
O
1H NMR spectrum for Compound 20
300 MHz, CDCl3
f1 (ppm)

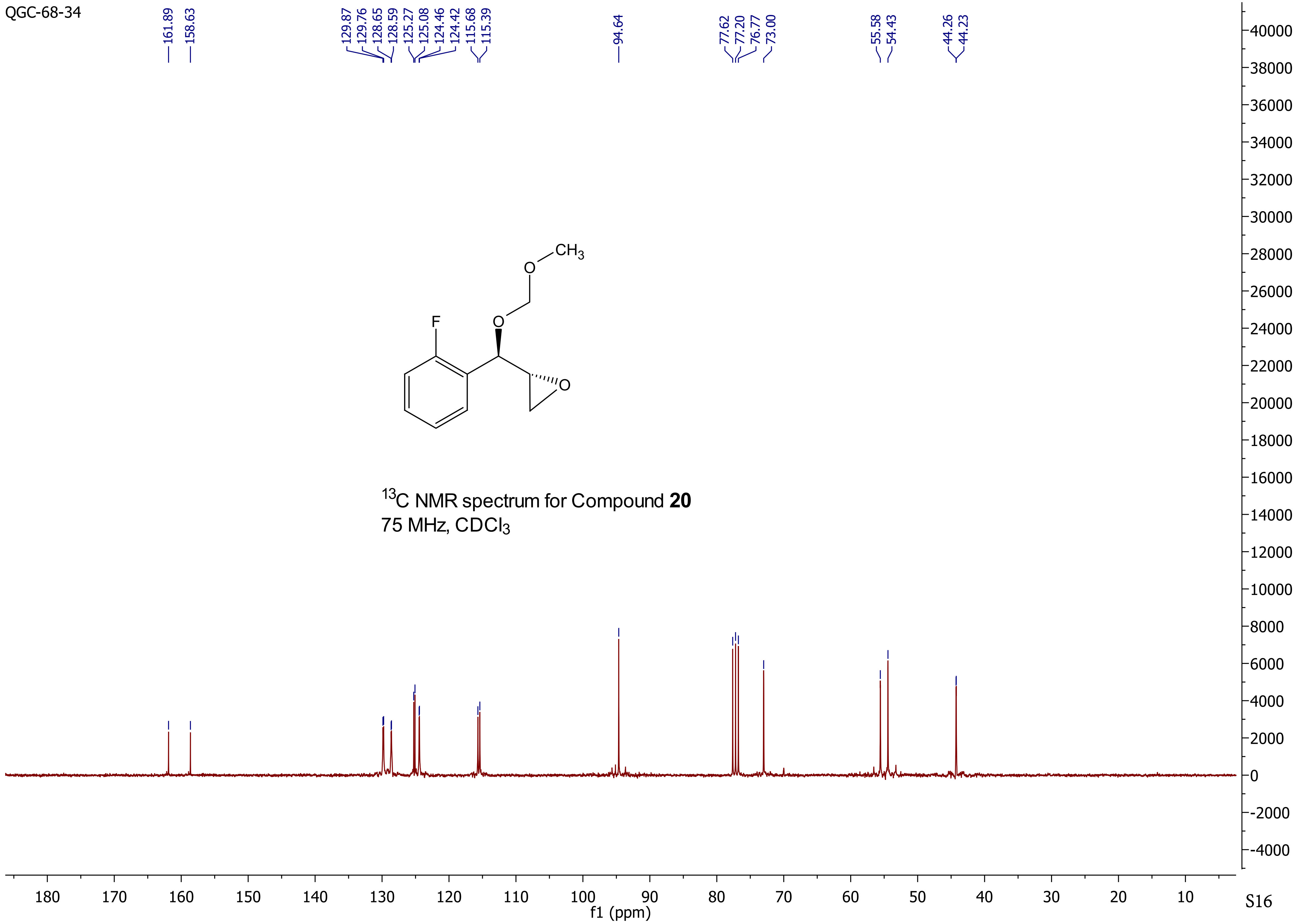

^{13}C NMR spectrum for Compound **20**
75 MHz, $CDCl_3$

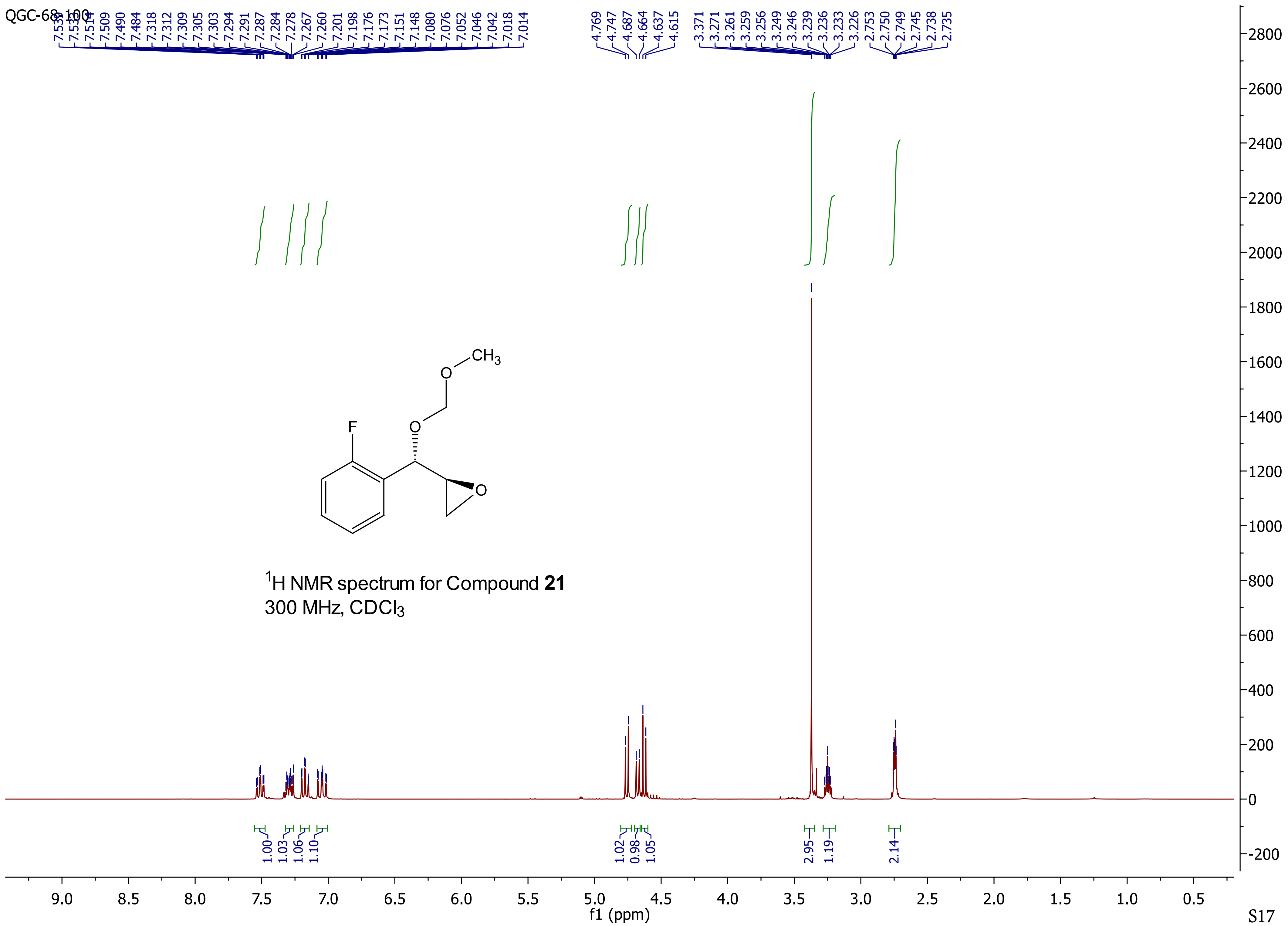

^{1}H NMR spectrum for Compound **21**
300 MHz, $CDCl_3$

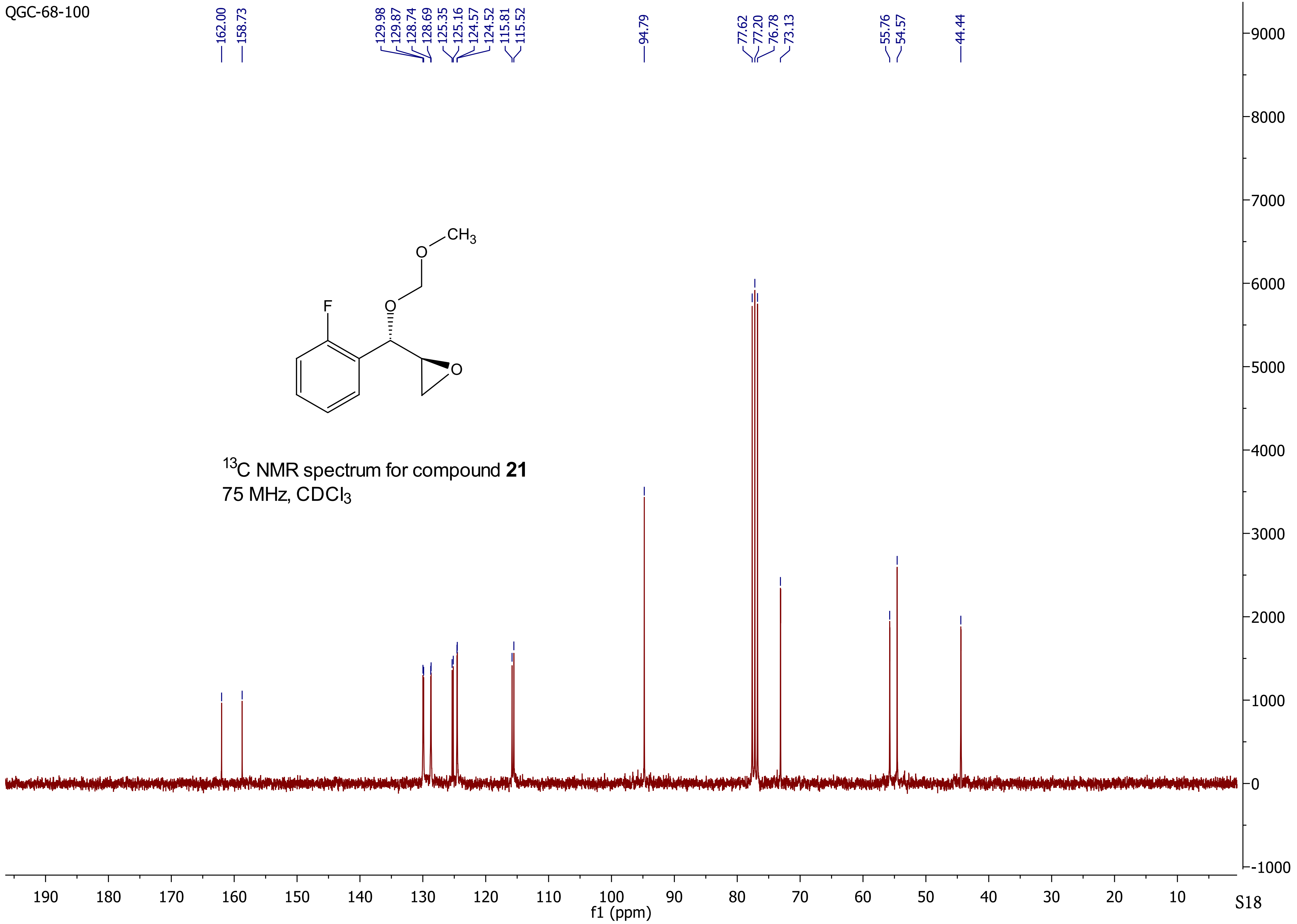

^{13}C NMR spectrum for compound **21**
75 MHz, $CDCl_3$

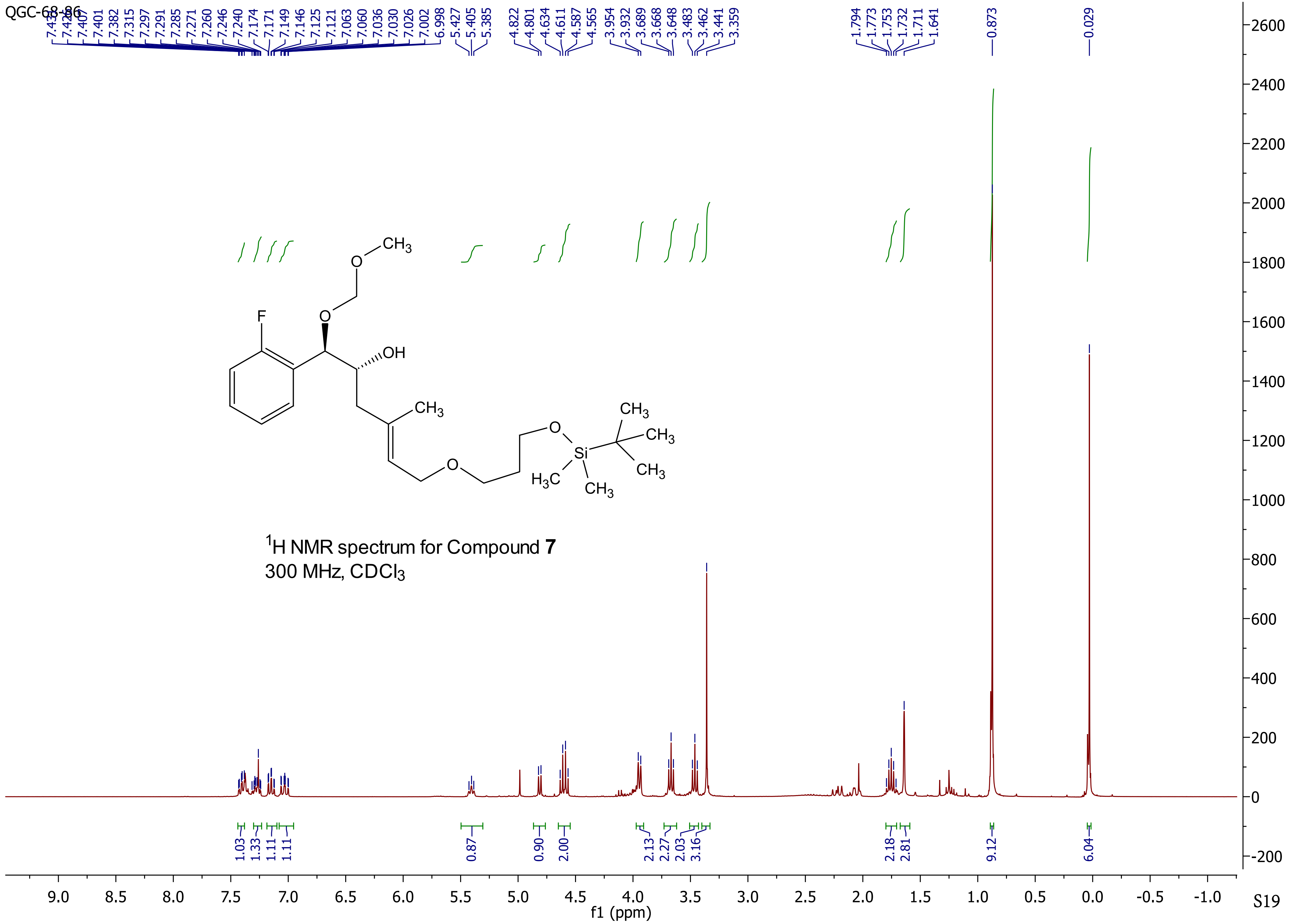

^{1}H NMR spectrum for Compound **7**

300 MHz, $CDCl_3$

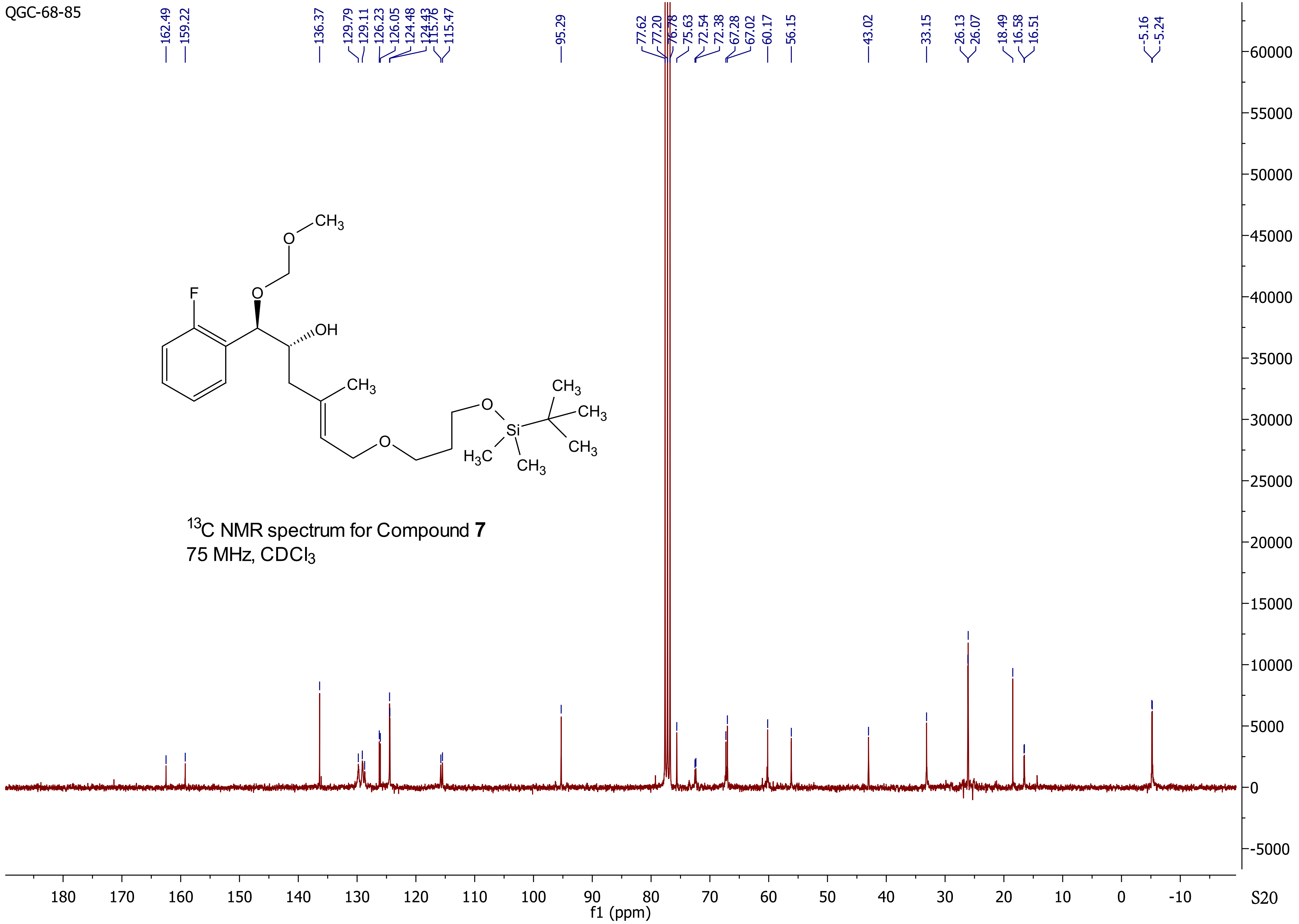

^{13}C NMR spectrum for Compound **7**
75 MHz, $CDCl_3$

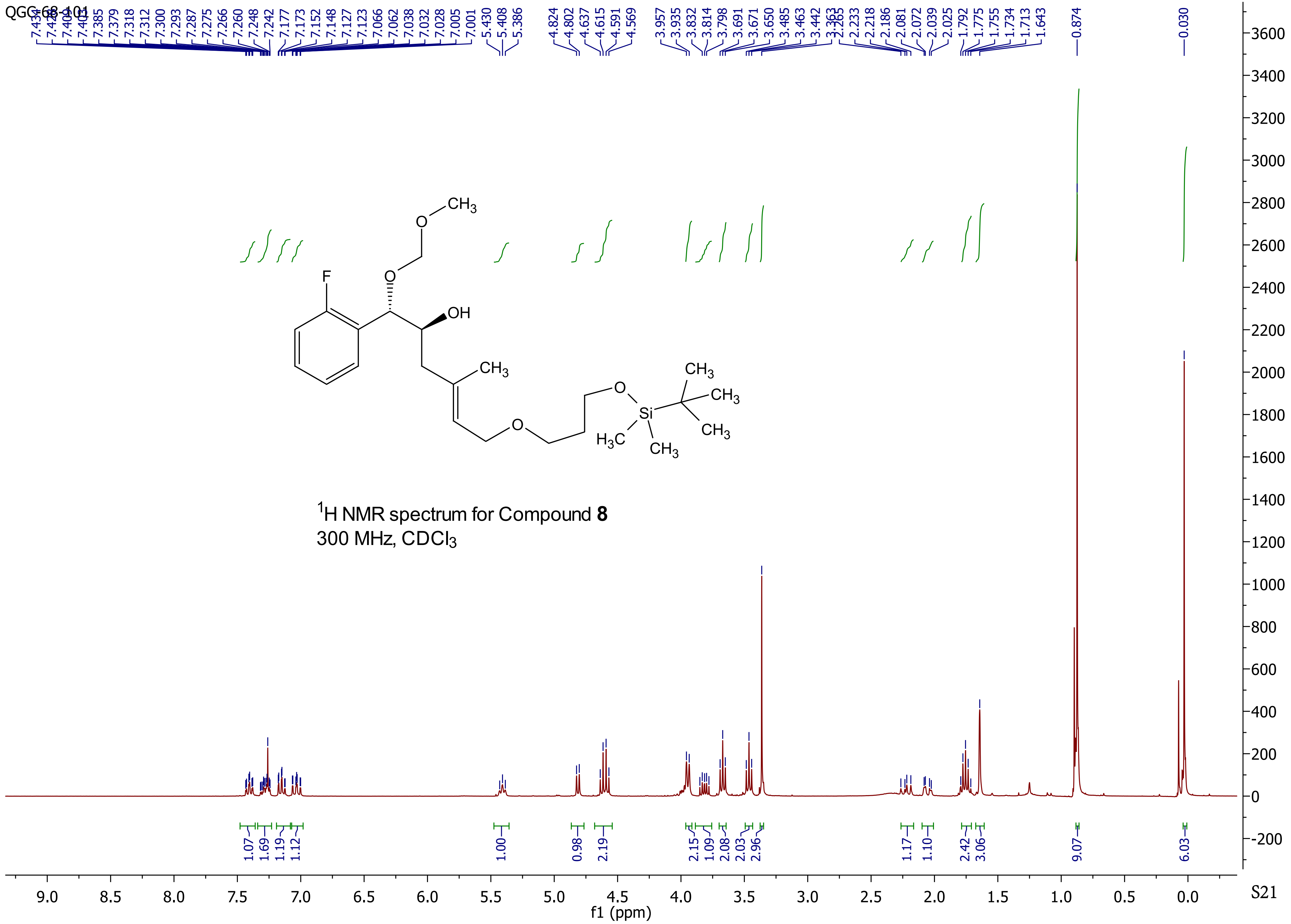

^{1}H NMR spectrum for Compound **8**
300 MHz, $CDCl_3$

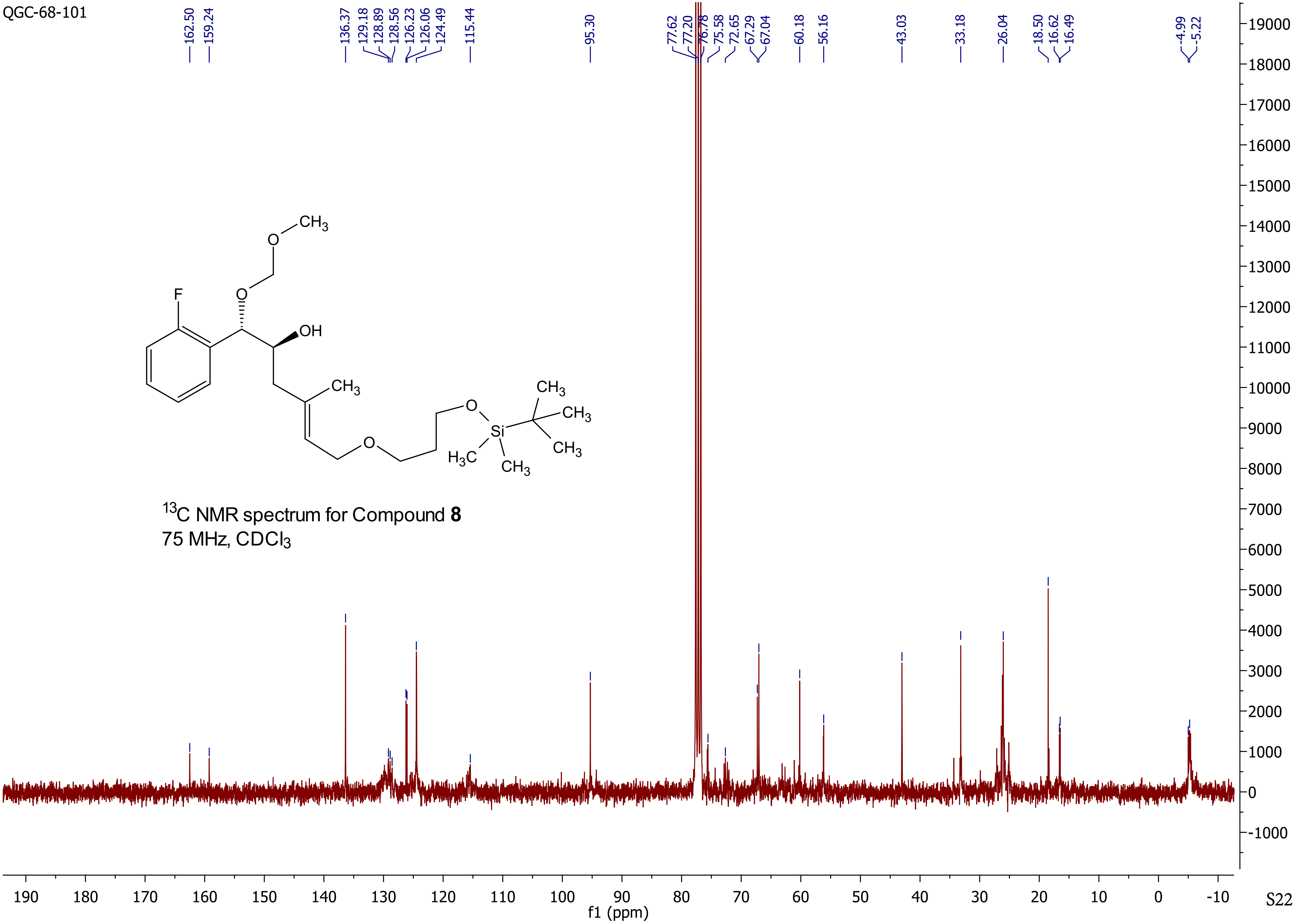

^{13}C NMR spectrum for Compound **8**
75 MHz, $CDCl_3$

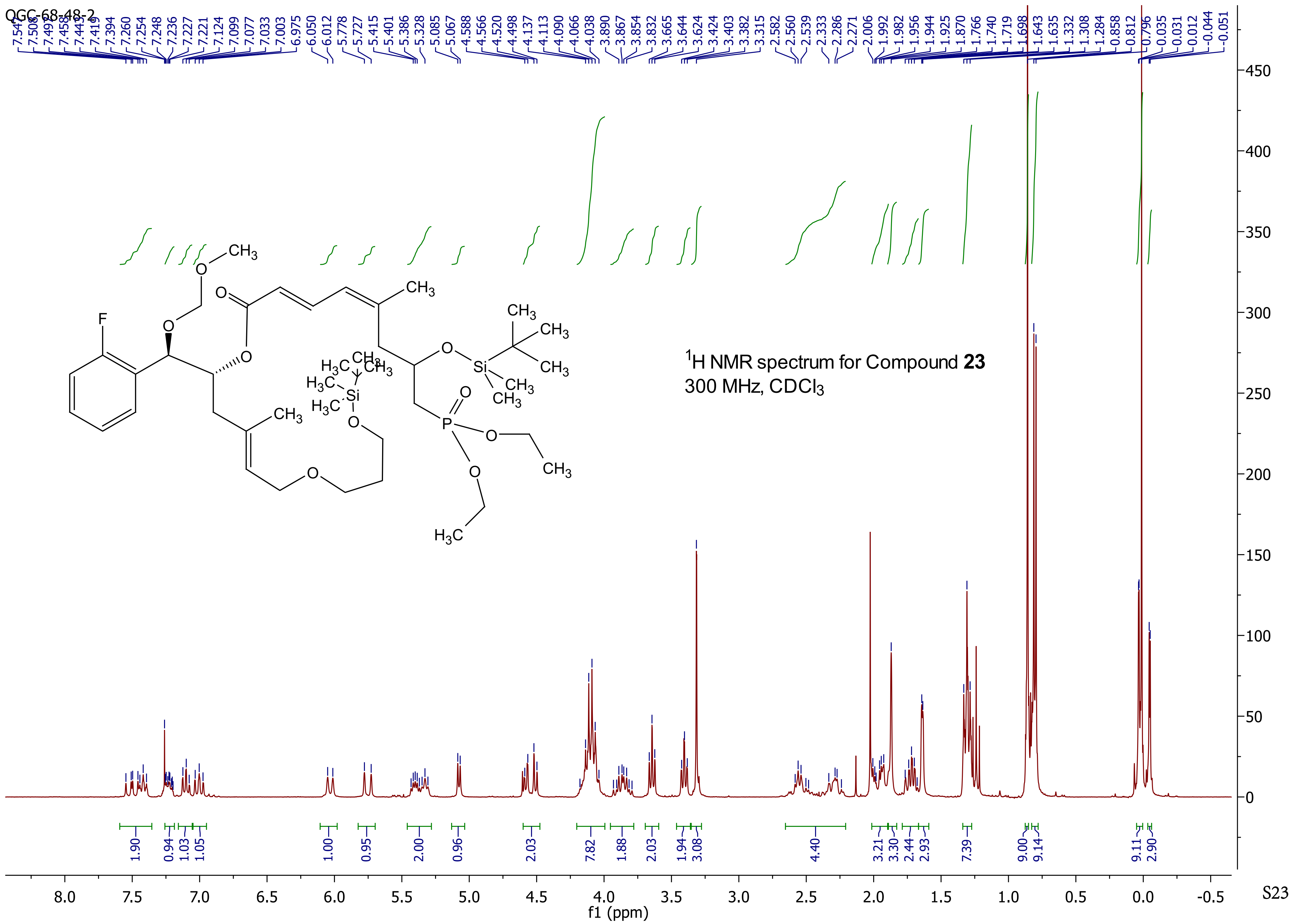
QGC-68-48-2
^{1}H NMR spectrum for Compound **23**
300 MHz, $CDCl_3$
f1 (ppm)

S23

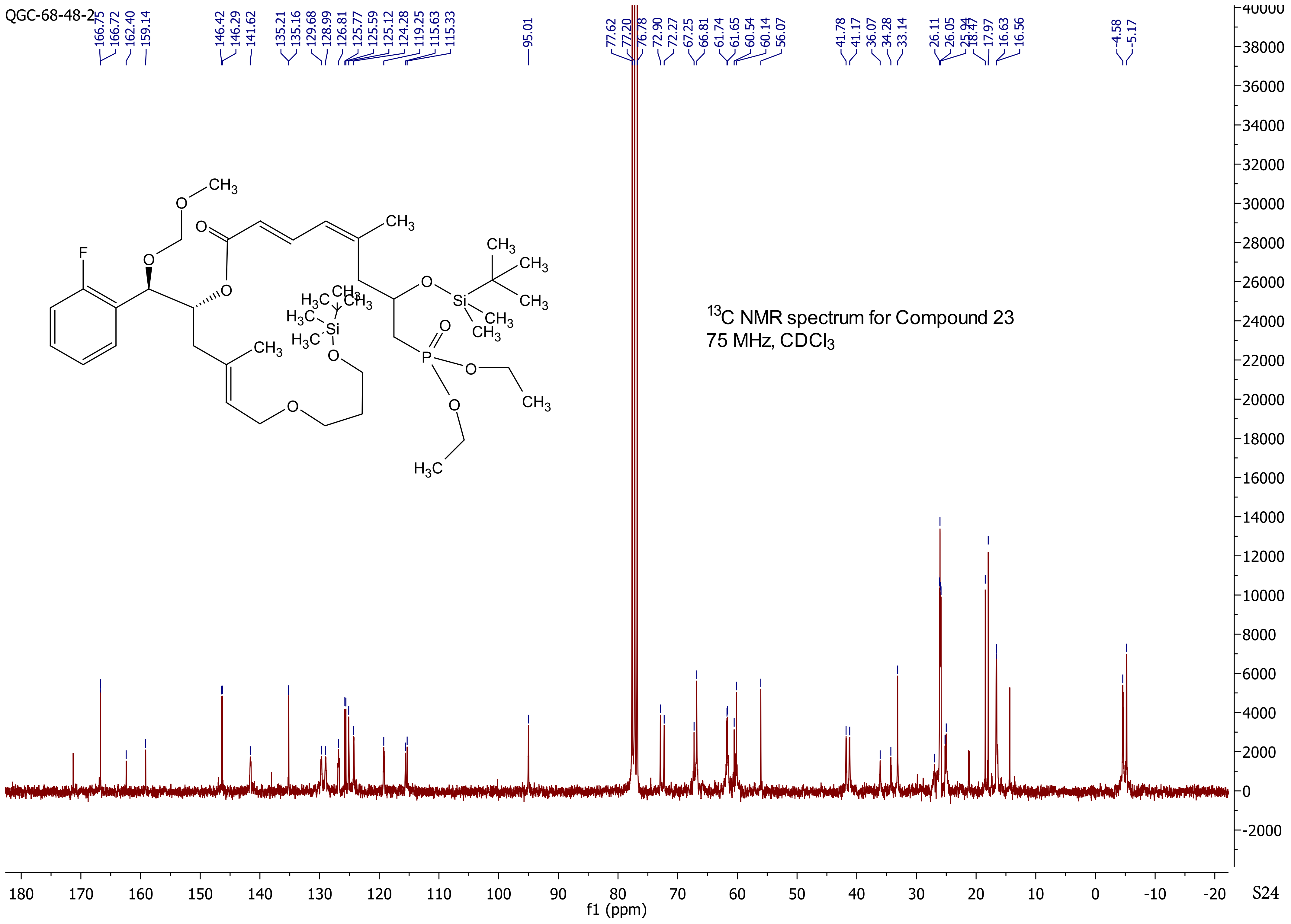
QGC-68-48-2
166.75
166.72
162.40
159.14
146.42
146.29
141.62
135.21
135.16
129.68
128.99
126.81
125.77
125.59
125.12
124.28
119.25
115.63
115.33
95.01
77.62
77.20
76.78
72.90
72.27
67.25
66.81
61.74
61.65
60.54
60.14
56.07
41.78
41.17
36.07
34.28
33.14
26.11
26.05
25.94
18.47
17.97
16.63
16.56
-4.58
-5.17
^{13}C NMR spectrum for Compound 23
75 MHz, $CDCl_3$
f1 (ppm)

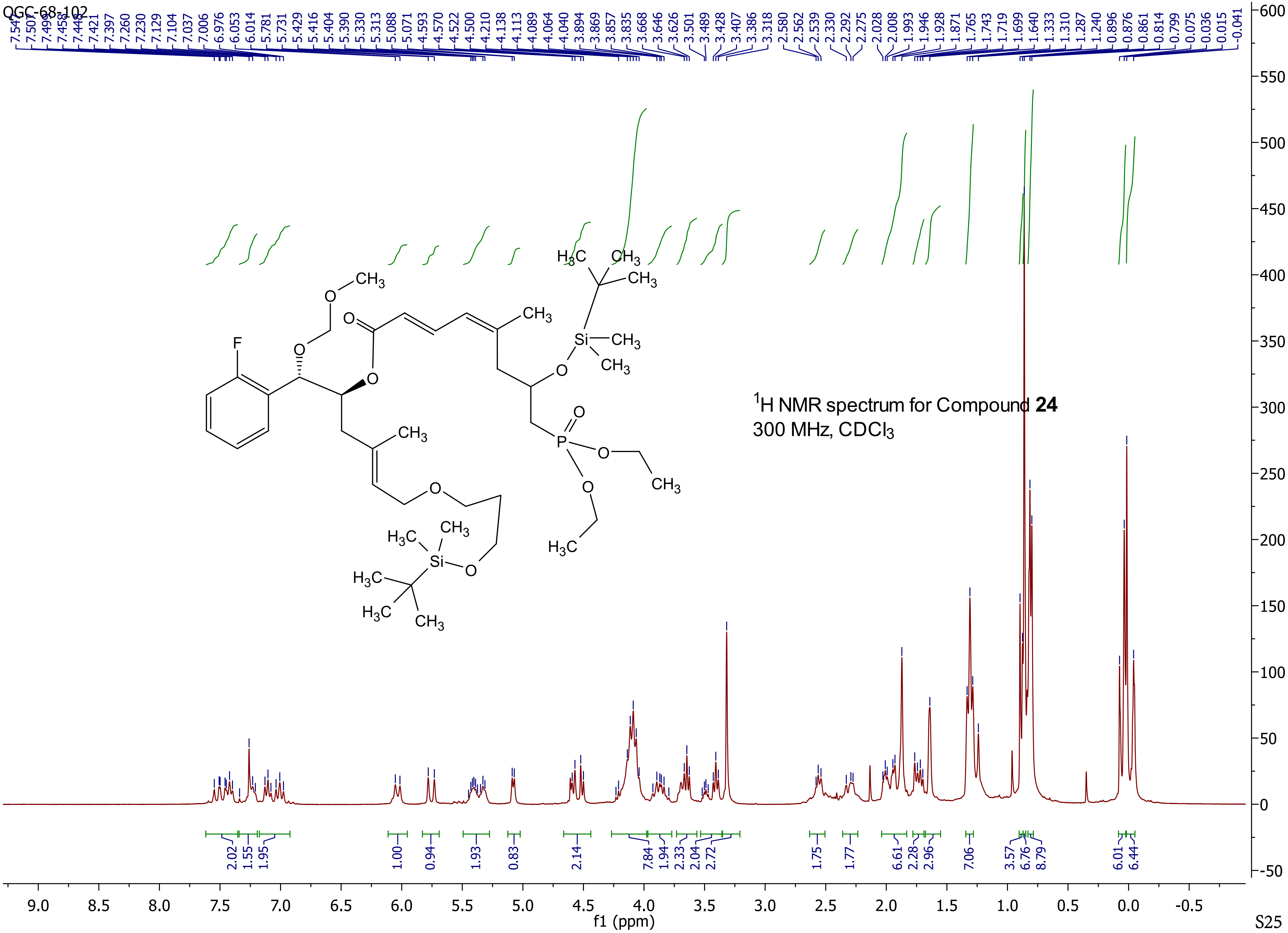
QGC-68-102
1H NMR spectrum for Compound 24
300 MHz, CDCl3
f1 (ppm)

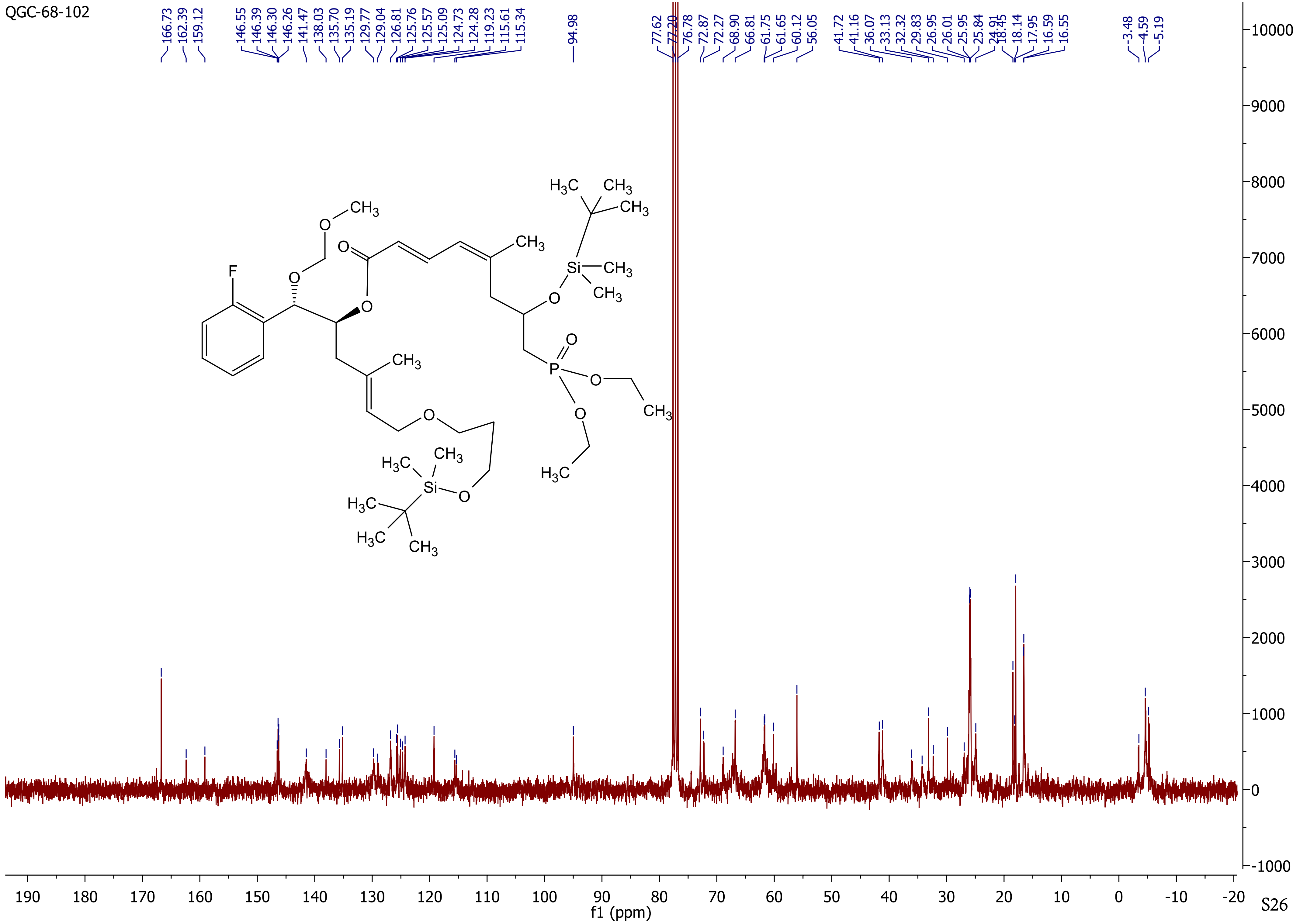
QGC-68-102
166.73
162.39
159.12
146.55
146.39
146.30
146.26
141.47
138.03
135.70
135.19
129.77
129.04
126.81
125.76
125.57
125.09
124.73
124.28
119.23
115.61
115.34
94.98
77.62
77.20
76.78
72.87
72.27
68.90
66.81
61.75
61.65
60.12
56.05
41.72
41.16
36.07
33.13
32.32
29.83
26.95
26.01
25.95
25.84
24.91
18.45
18.14
17.95
16.59
16.55
-3.48
-4.59
-5.19
f1 (ppm)

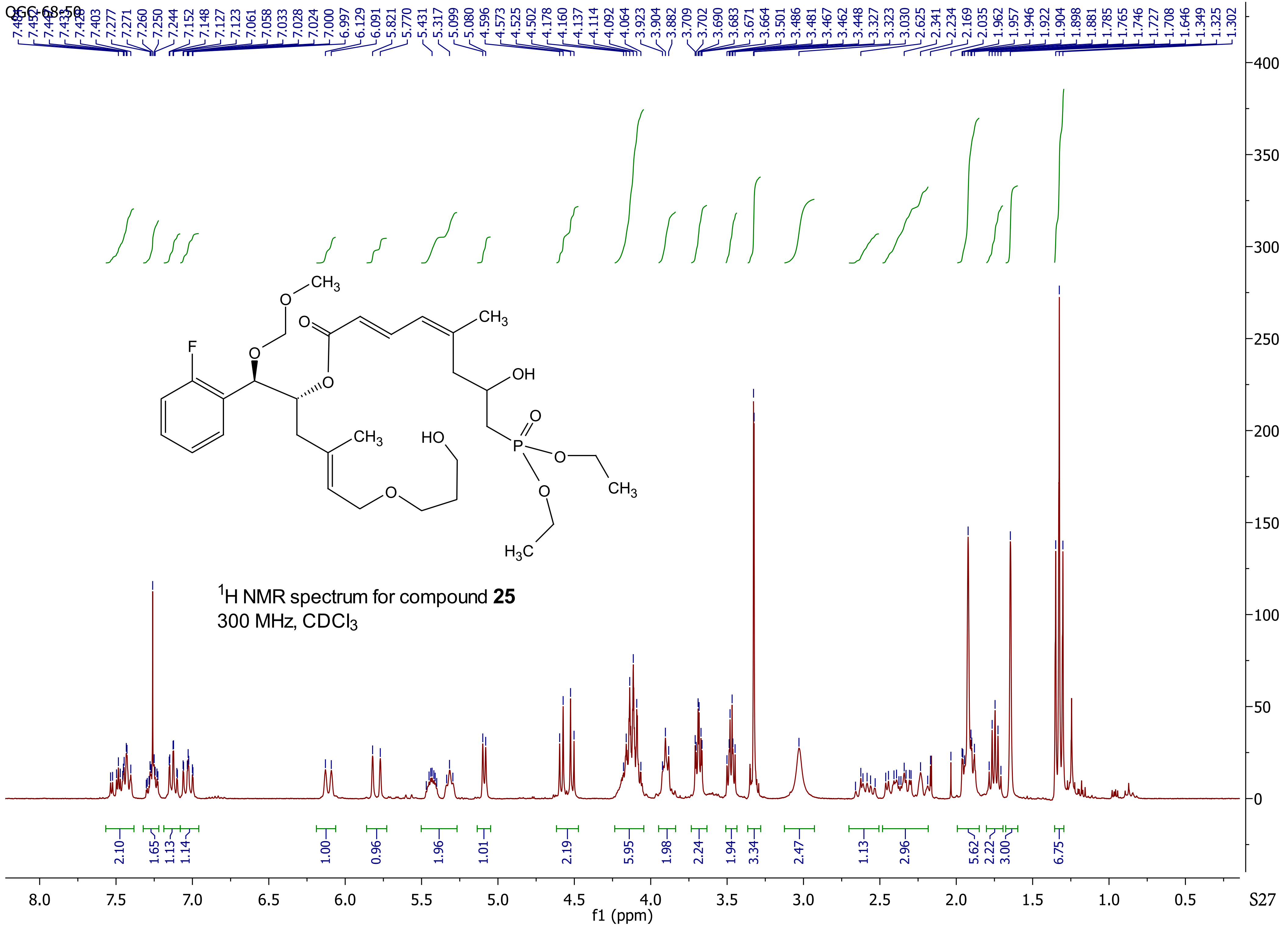
QCC-68-50
F
O
O
CH3
O
O
CH3
OH
O
P
O
CH3
O
H3C
CH3
HO
O
1H NMR spectrum for compound 25
300 MHz, CDCl3
2.10
1.65
1.13
1.14
1.00
0.96
1.96
1.01
2.19
5.95
1.98
2.24
1.94
3.34
2.47
1.13
2.96
5.62
2.22
3.00
6.75
f1 (ppm)

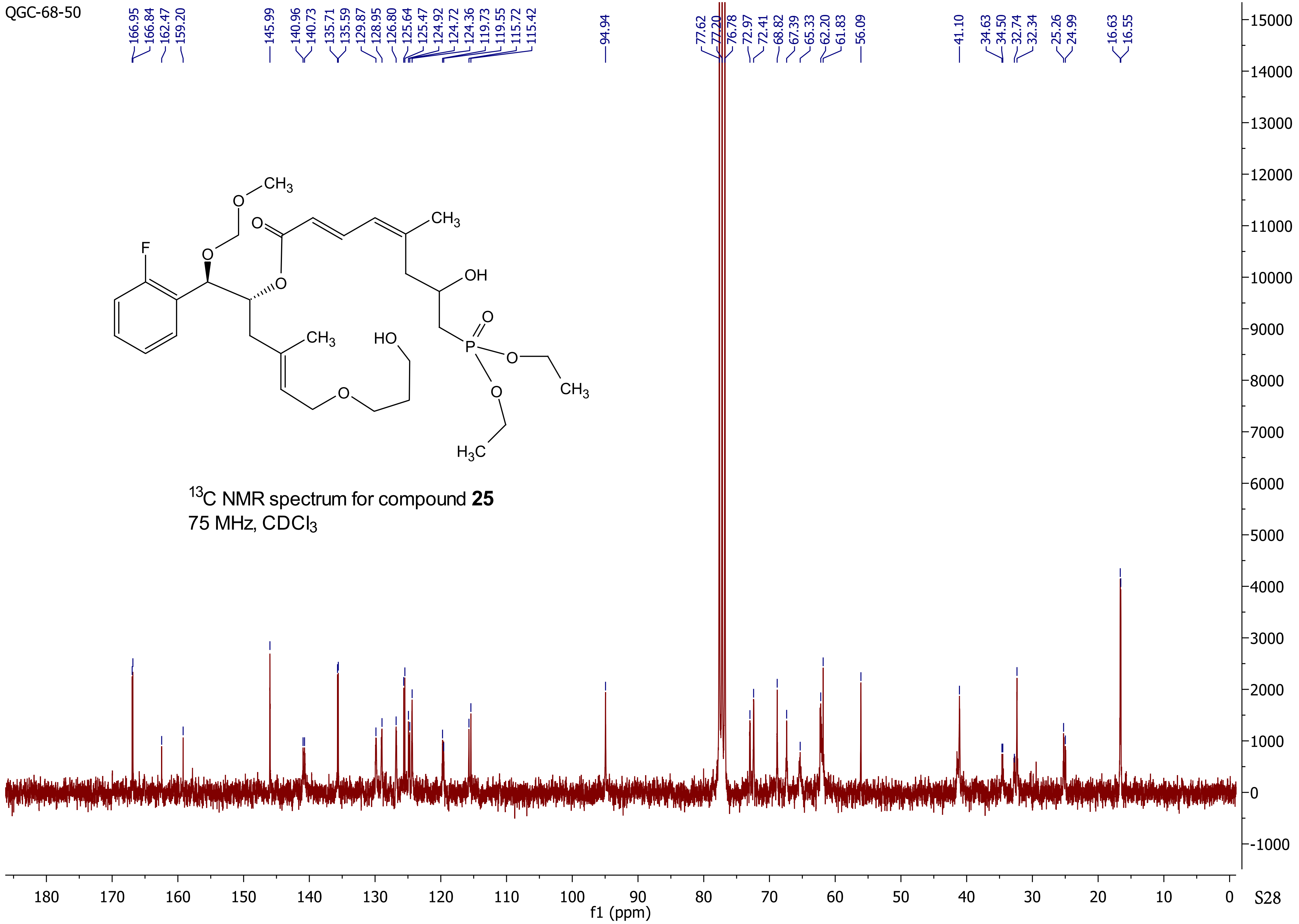

^{13}C NMR spectrum for compound **25**

75 MHz, $CDCl_3$

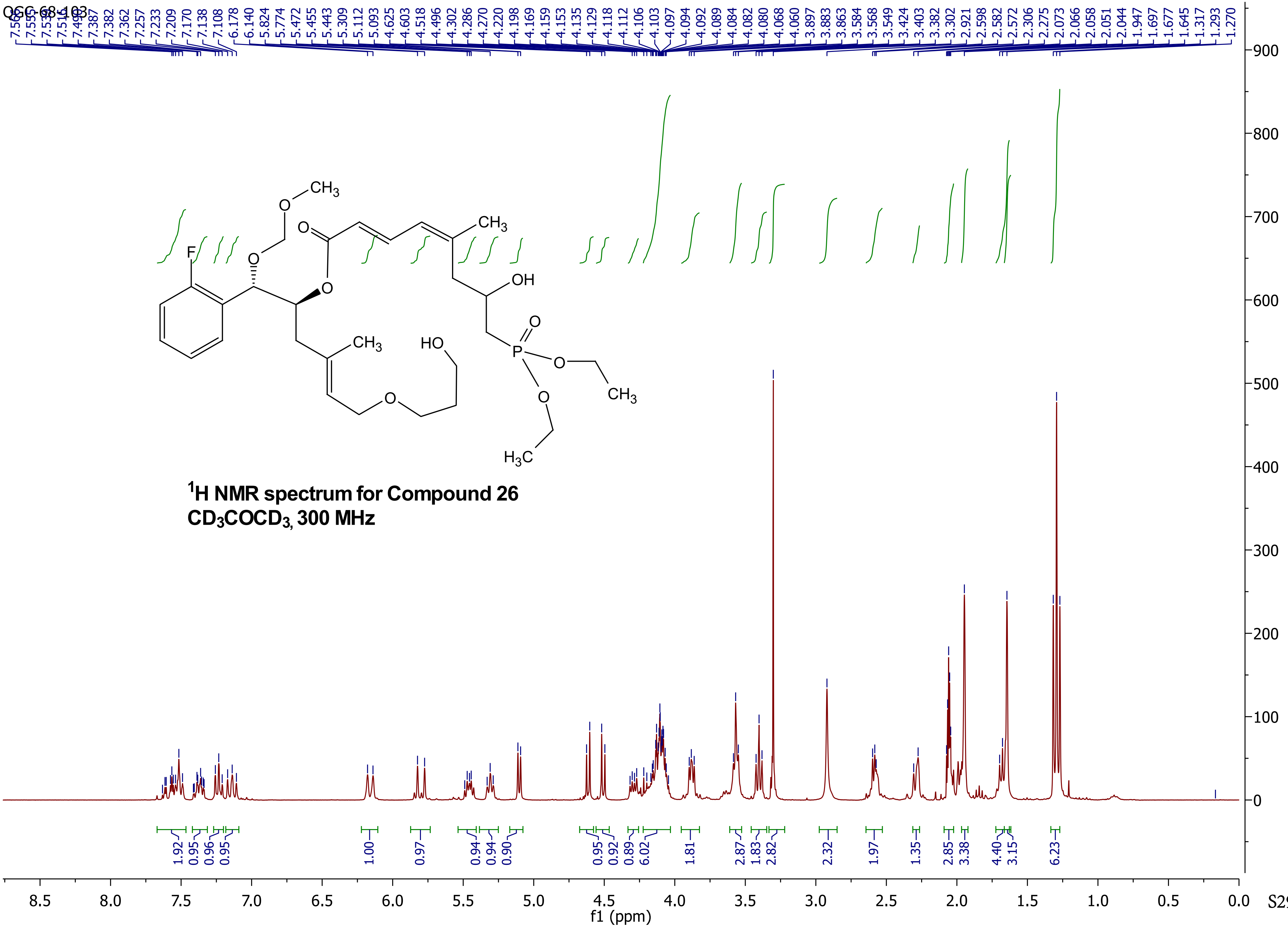
QCC-68-103
1H NMR spectrum for Compound 26
CD3COCD3, 300 MHz
f1 (ppm)

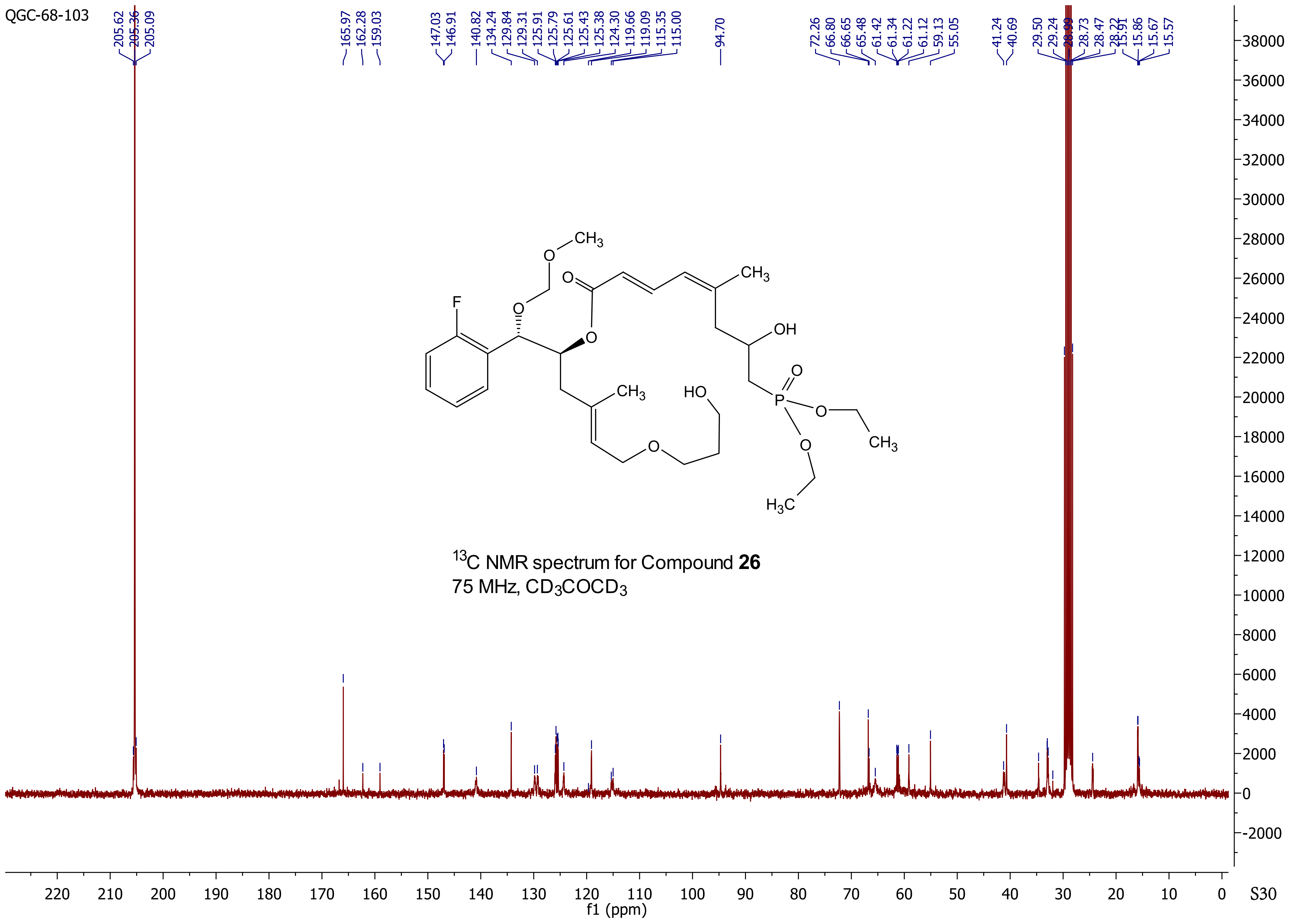
QGC-68-103
205.62
205.36
205.09
165.97
162.28
159.03
147.03
146.91
140.82
134.24
129.84
129.31
125.91
125.79
125.61
125.43
125.38
124.30
119.66
119.09
115.35
115.00
94.70
72.26
66.80
66.65
65.48
61.42
61.34
61.22
61.12
59.13
55.05
41.24
40.69
29.50
29.24
28.99
28.73
28.47
28.22
15.91
15.86
15.67
15.57
^{13}C NMR spectrum for Compound **26**
75 MHz, CD_3COCD_3
f1 (ppm)

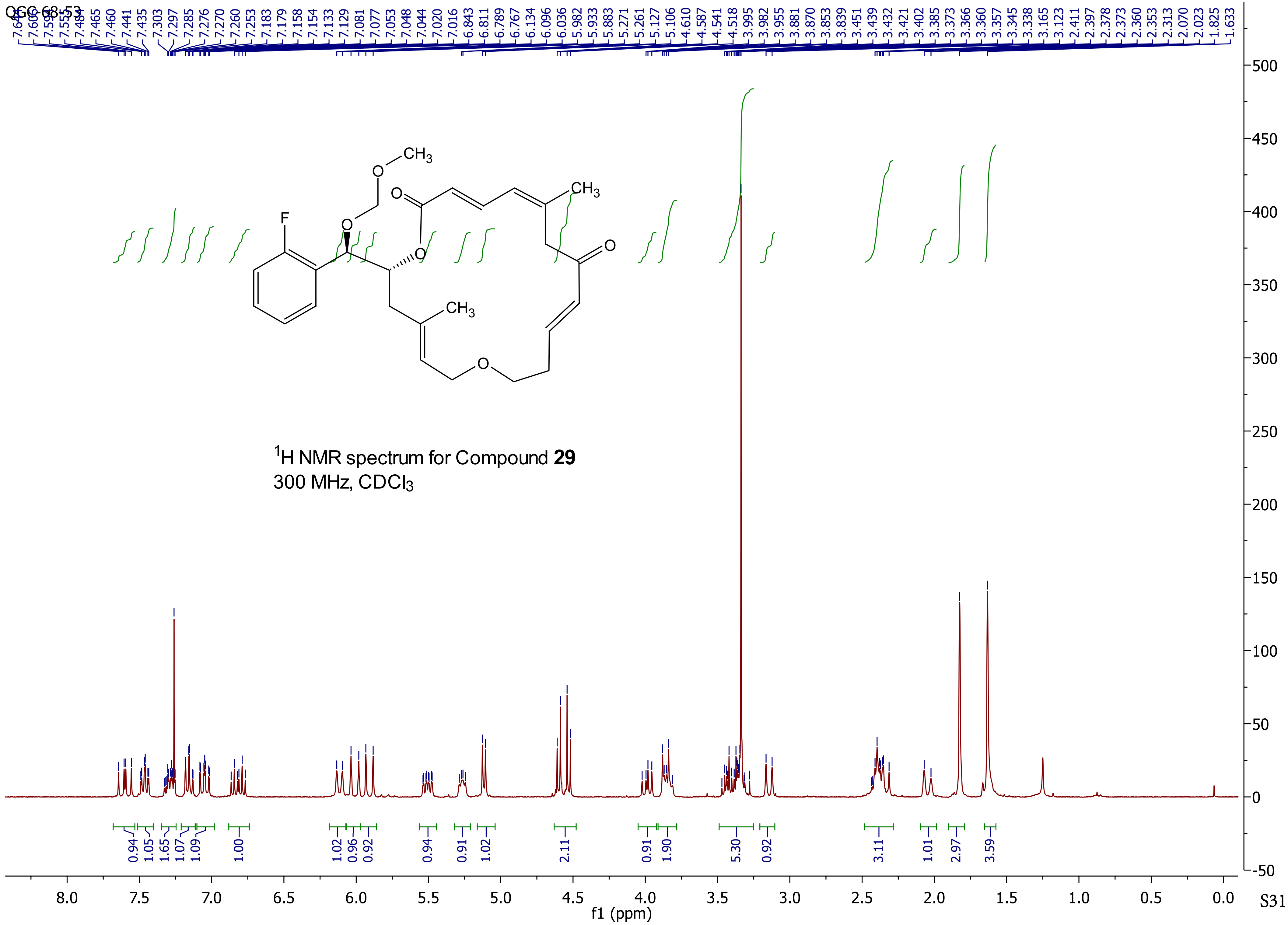

1H NMR spectrum for Compound **29**
300 MHz, $CDCl_3$

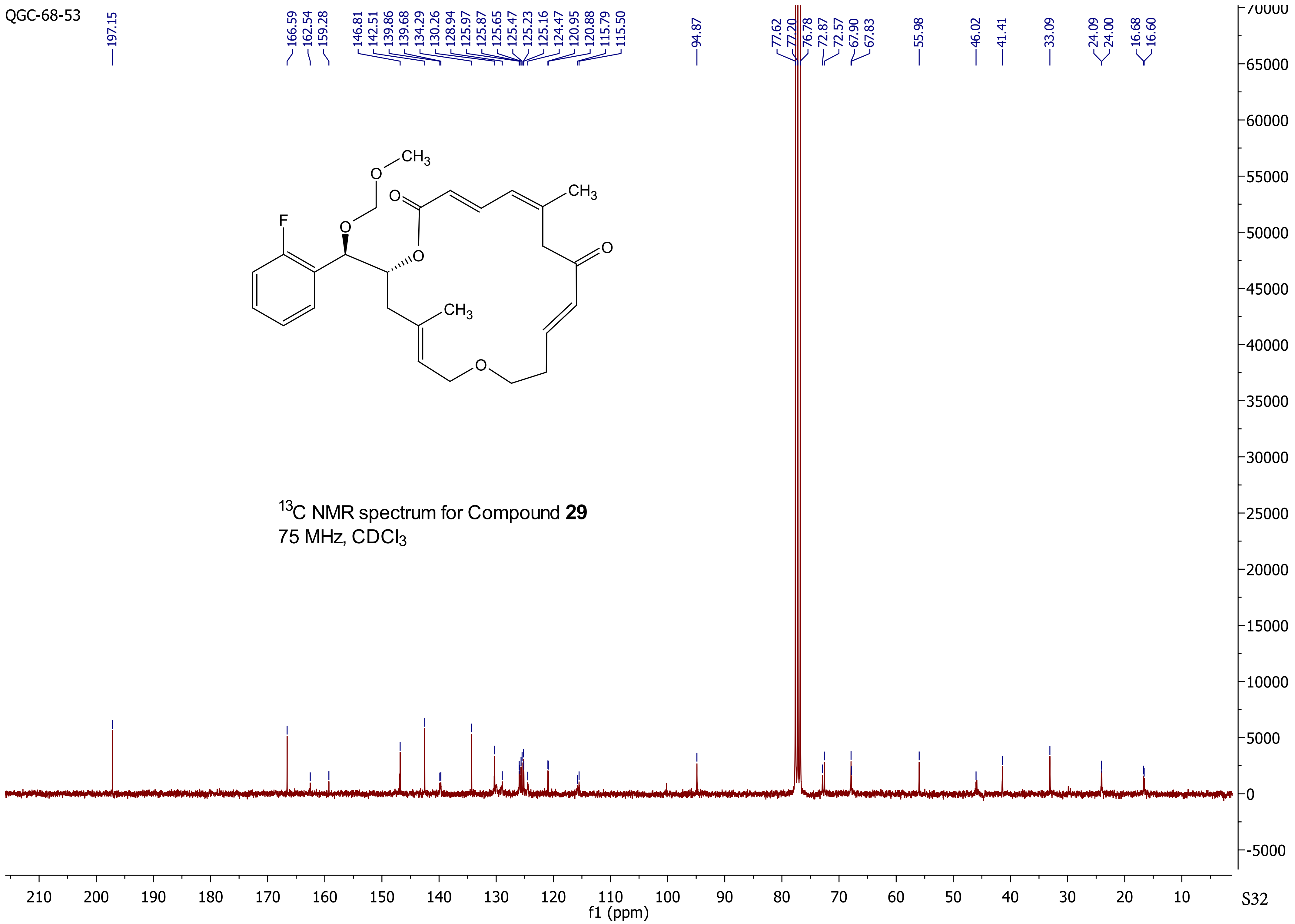

^{13}C NMR spectrum for Compound **29**
75 MHz, $CDCl_3$

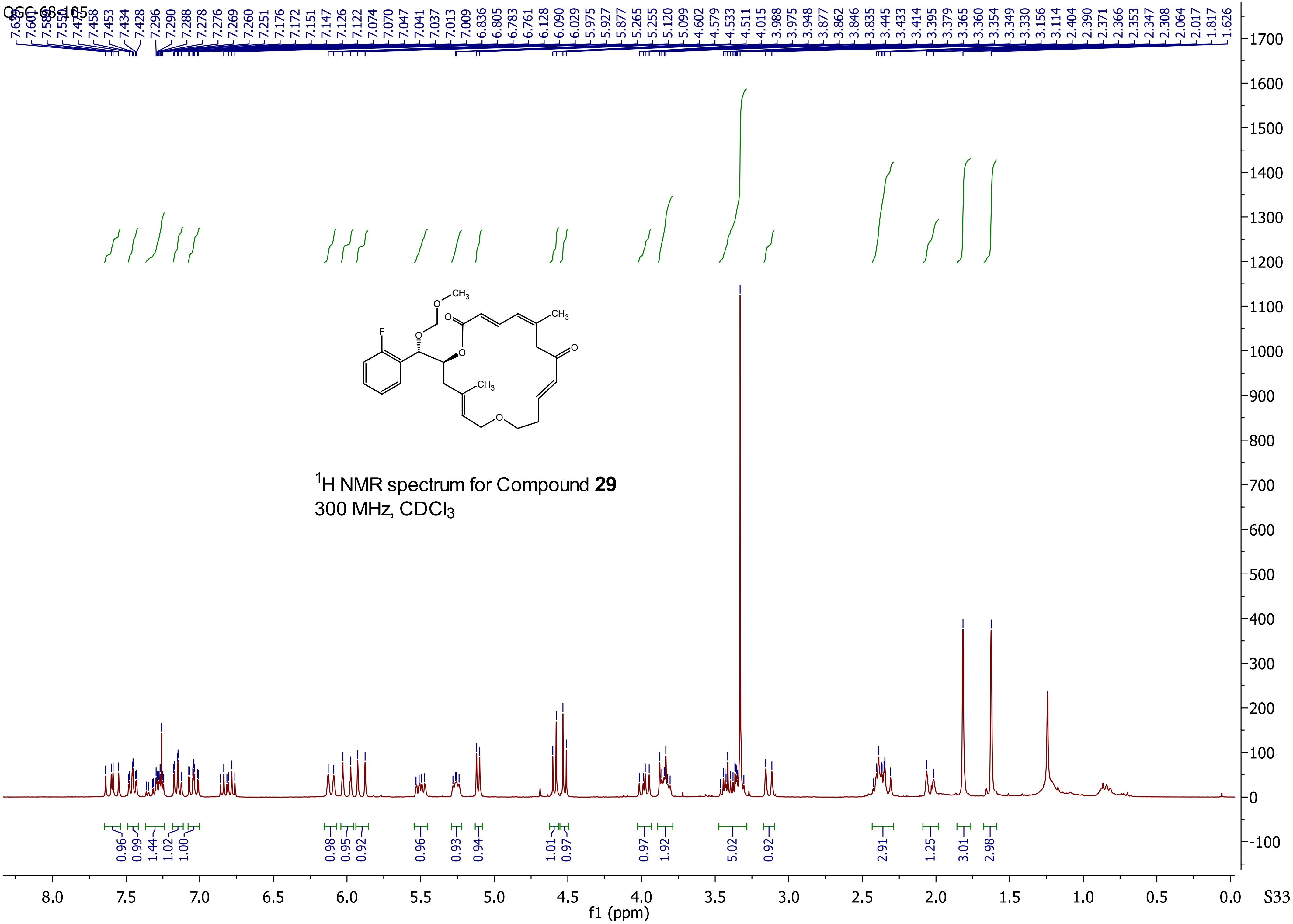
QCC-68-105
1H NMR spectrum for Compound 29
300 MHz, CDCl3
f1 (ppm)

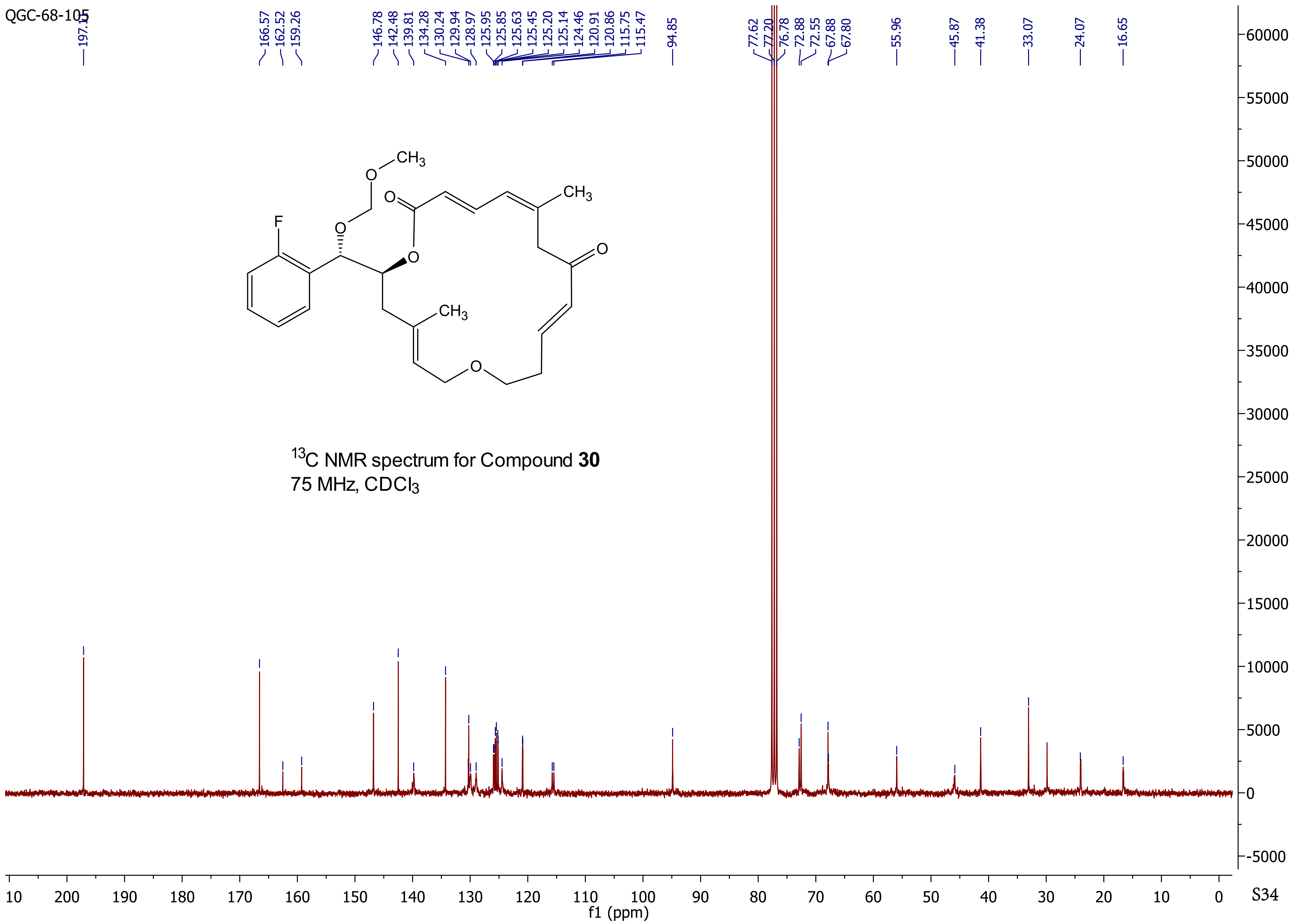

^{13}C NMR spectrum for Compound **30**
75 MHz, $CDCl_3$

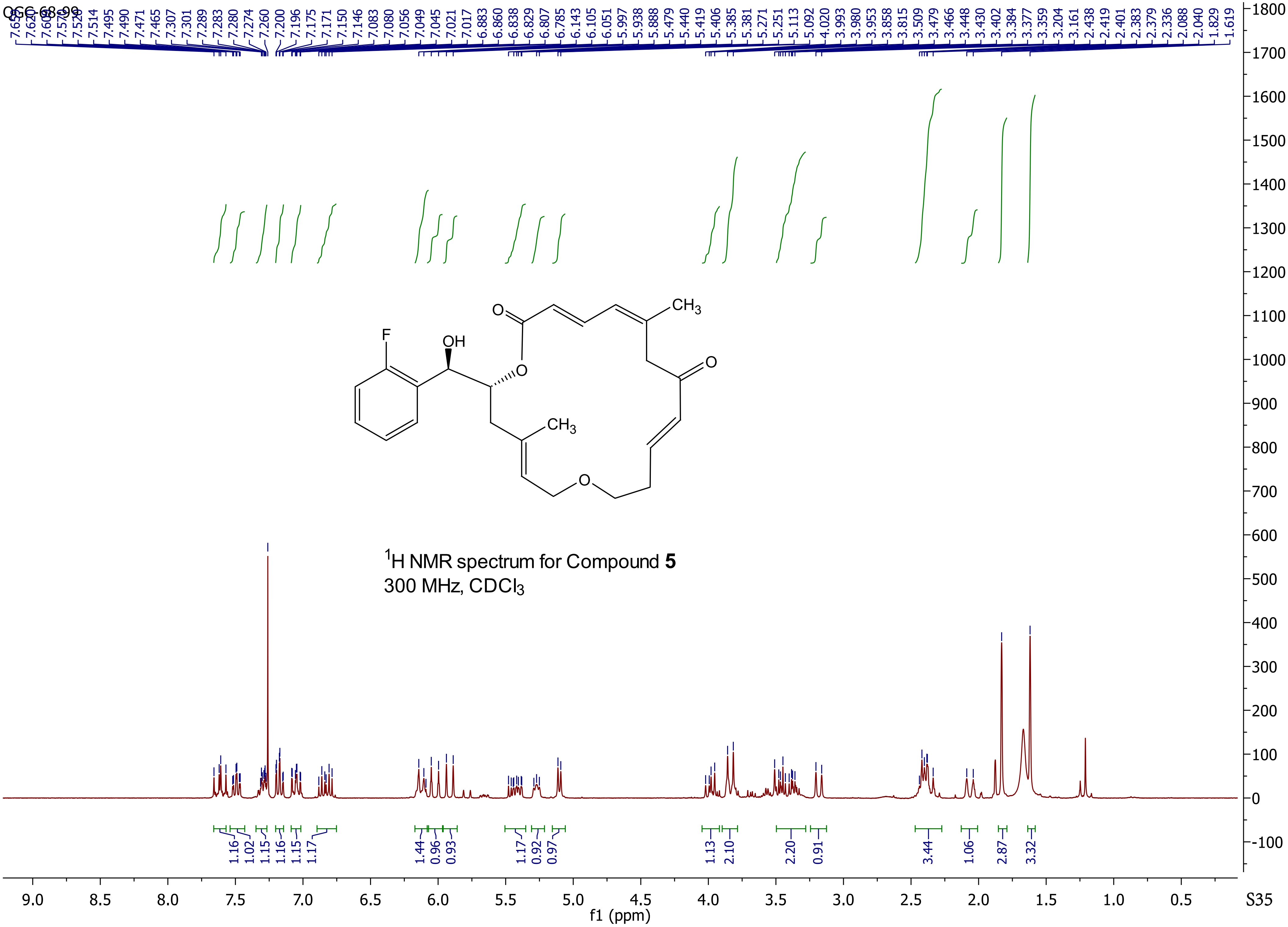
QCC-68-99
F
OH
O
O
CH3
O
CH3
O
O
1H NMR spectrum for Compound 5
300 MHz, CDCl3
f1 (ppm)

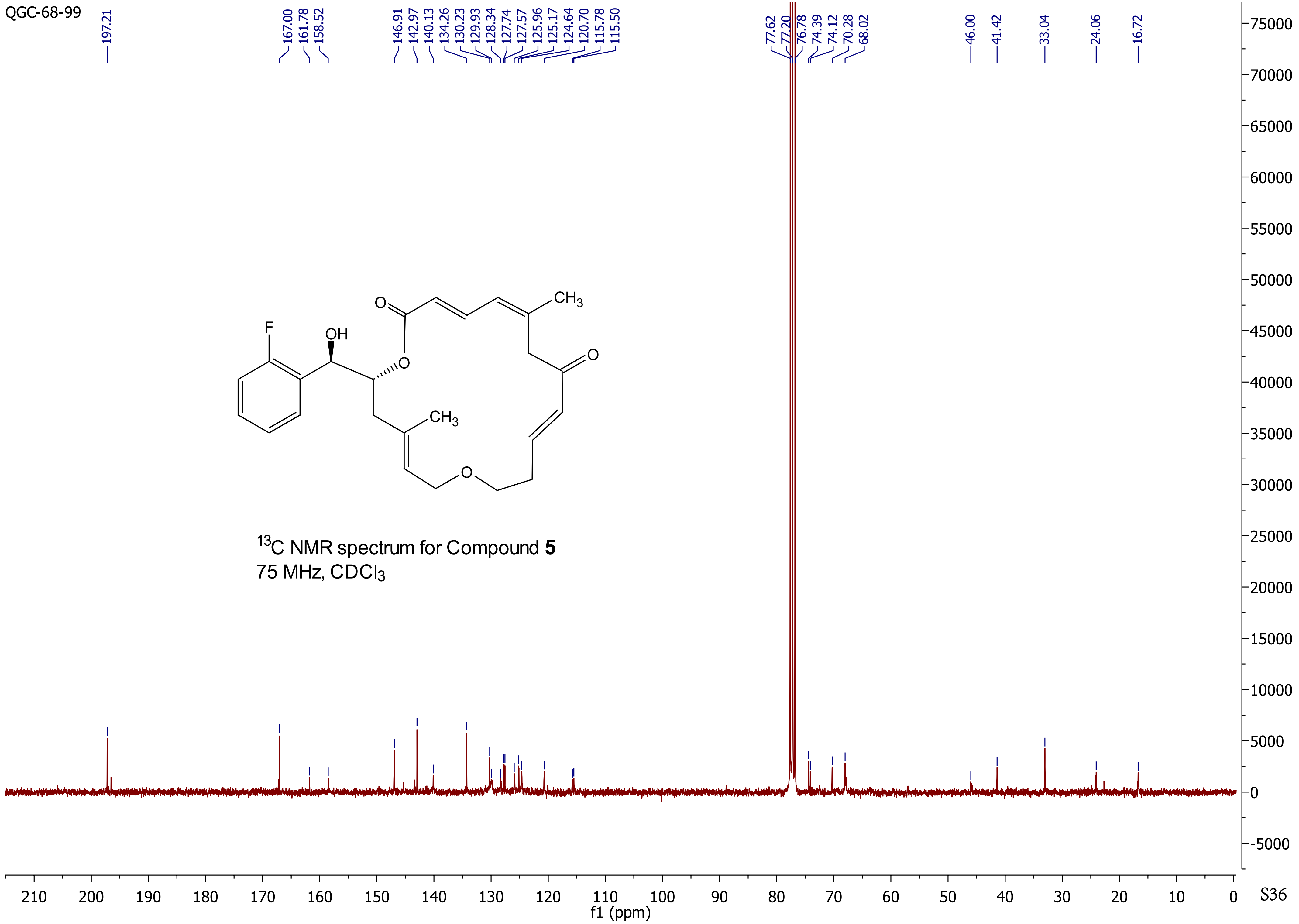
QGC-68-99
197.21
167.00
161.78
158.52
146.91
142.97
140.13
134.26
130.23
129.93
128.34
127.74
127.57
125.96
125.17
124.64
120.70
115.78
115.50
77.62
77.20
76.78
74.39
74.12
70.28
68.02
46.00
41.42
33.04
24.06
16.72
F
OH
O
O
O
CH3
CH3
O
O
^{13}C NMR spectrum for Compound **5**
75 MHz, $CDCl_3$
f1 (ppm)

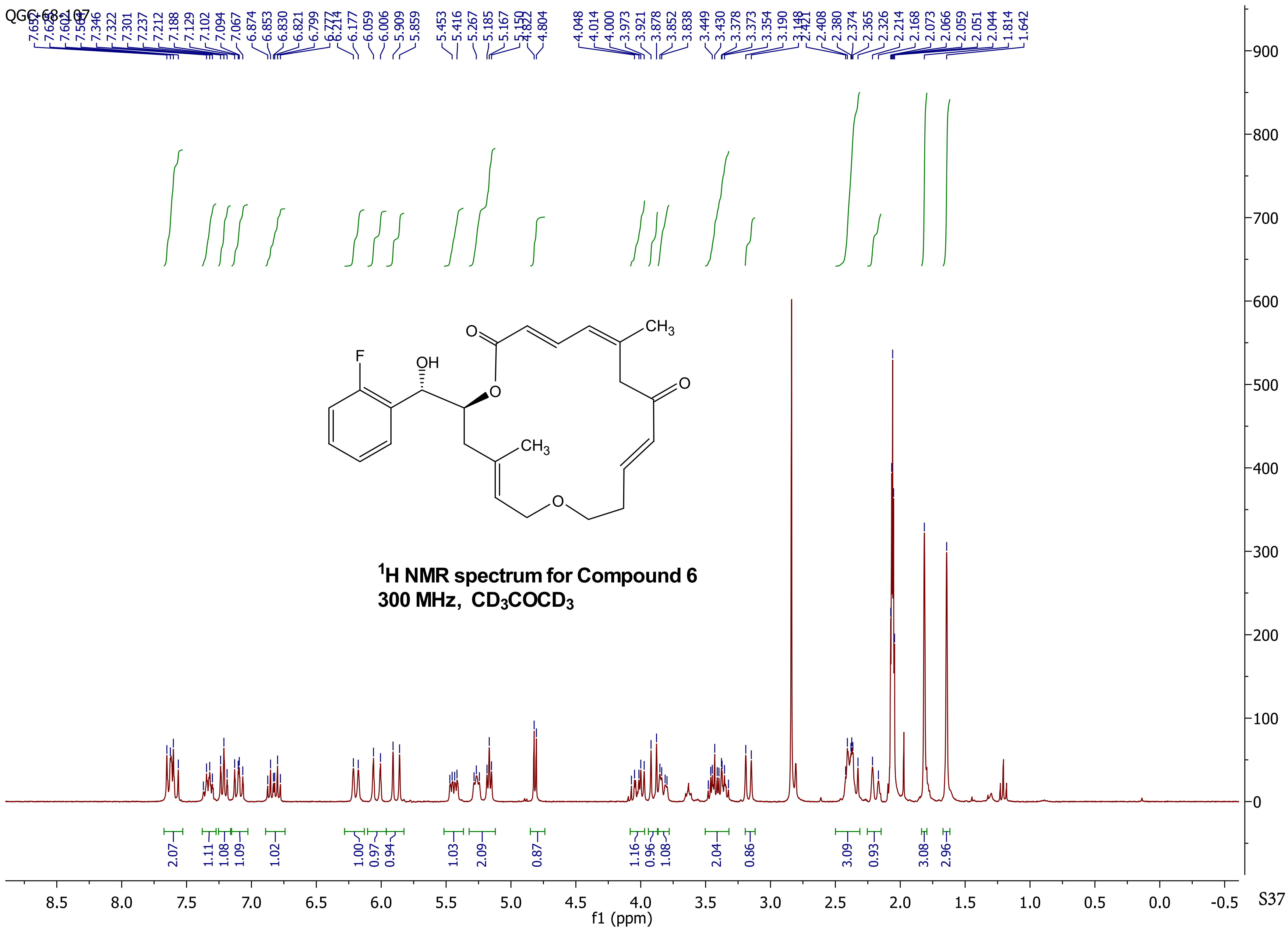
QGC-68-107
^{1}H NMR spectrum for Compound 6
300 MHz, CD_3COCD_3
f1 (ppm)

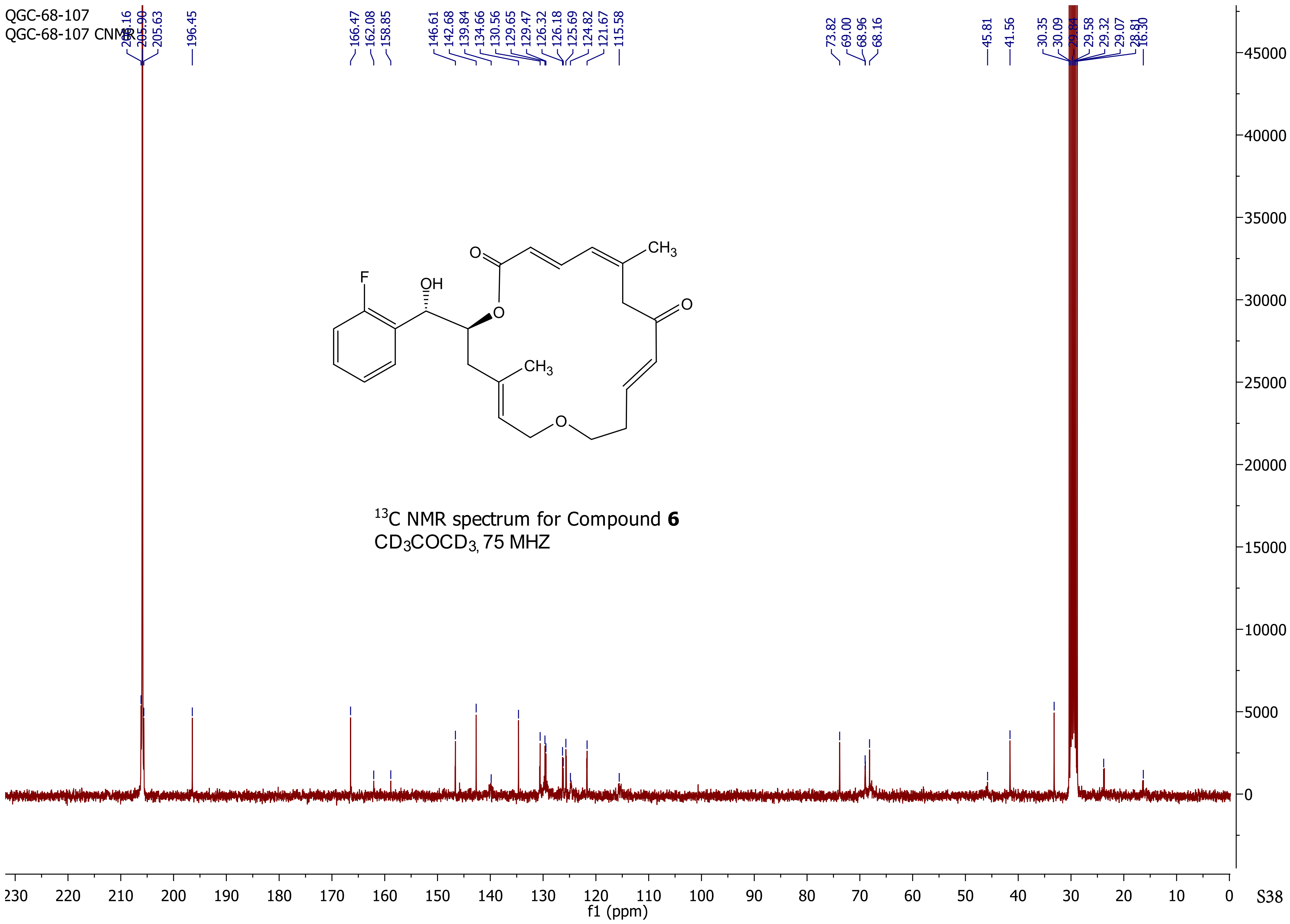
QGC-68-107
QGC-68-107 CNMR
205.63
196.45
166.47
162.08
158.85
146.61
142.68
139.84
134.66
130.56
129.65
129.47
126.32
126.18
125.69
124.82
121.67
115.58
73.82
69.00
68.96
68.16
45.81
41.56
30.35
30.09
29.84
29.58
29.32
29.07
16.30
F
OH
O
O
CH3
O
CH3
O
13C NMR spectrum for Compound 6
CD3COCD3, 75 MHZ
45000
40000
35000
30000
25000
20000
15000
10000
5000
0
230
220
210
200
190
180
170
160
150
140
130
120
110
100
90
80
70
60
50
40
30
20
10
0
f1 (ppm)
